# Chemical investigations into the biosynthesis of the gymnastatin and dankastatin alkaloids†

**DOI:** 10.1039/d1sc02613e

**Published:** 2021-05-31

**Authors:** Bingqi Tong, Bridget P. Belcher, Daniel K. Nomura, Thomas J. Maimone

**Affiliations:** Department of Chemistry, University of California–Berkeley Berkeley CA 94720 USA maimone@berkeley.edu; Novartis-Berkeley Center for Proteomics and Chemistry Technologies, University of California–Berkeley Berkeley CA 94720 USA; Departments of Nutritional Science and Toxicology, Cell and Molecular Biology, The Innovative Genomics Institute, University of California–Berkeley Berkeley CA 94720 USA

## Abstract

Electrophilic natural products have provided fertile ground for understanding how nature inhibits protein function using covalent bond formation. The fungal strain *Gymnascella dankaliensis* has provided an especially interesting collection of halogenated cytotoxic agents derived from tyrosine which feature an array of reactive functional groups. Herein we explore chemical and potentially biosynthetic relationships between architecturally complex gymnastatin and dankastatin members, finding conditions that favor formation of a given scaffold from a common intermediate. Additionally, we find that multiple natural products can also be formed from aranorosin, a non-halogenated natural product also produced by *Gymnascella* sp. fungi, using simple chloride salts thus offering an alternative hypothesis for the origins of these compounds in nature. Finally, growth inhibitory activity of multiple members against human triple negative breast cancer cells is reported.

## Abstract 2

Total synthesis sheds light on biosynthetic relationships among the chlorinated gymnastatin and dankastatin alkaloids.

## Graphical abstract

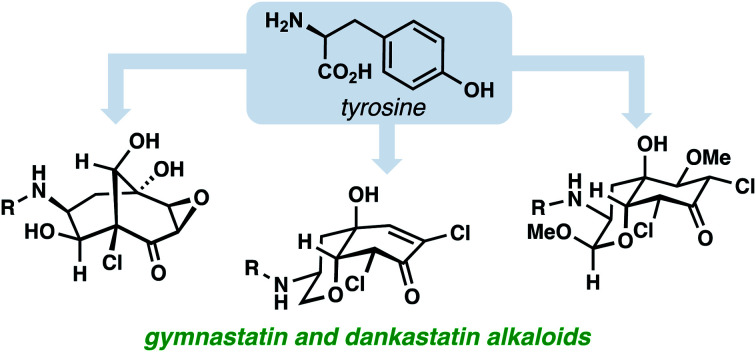

## Introduction

Natural products have long been a rich source of medicinal agents and sources of inspiration for the design of numerous clinical candidates and FDA-approved drugs.^1^ Among these, members that interact with protein targets through covalent bond formation have the potential to open up new areas of druggable space, provide sustained target engagement, and confer unique selectivity as a result of architectural complementarity to many fully synthetic small molecules.^2^ Moreover, natural products featuring more than one covalent warhead offer the prospect of engaging several nucleophilic protein residues and potentially multiple protein partners.^3^

**Fig. 1:**
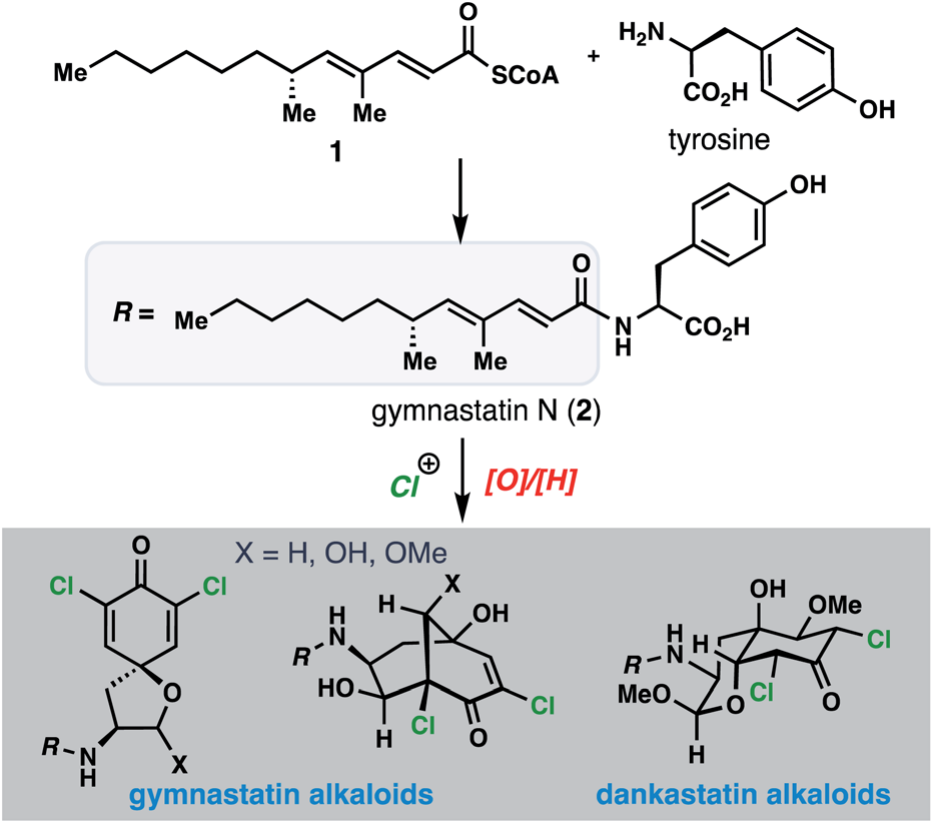
Tyrosine-derived alkaloids from *Gymnascella* sp. fungi.

Against this backdrop, we became interested in the fascinating array of chlorinated gymnastatin and dankastatin alkaloids first disclosed in 1997 from the fungal strain *Gymnascella dankaliensis* isolated from the sponge *Halichondria japonica* (Fig. 1).^4^ Presumably produced through the merger of tyrosine and a 14-carbon polyketide fragment (see **1**) to first generate gymnastatin N (**2**), electrophilic halogenation and various oxidative cyclization reactions create a small library of architecturally complex natural products from a common and simple precursor. Gymnastatin and dankastatin alkaloids possess a veritable treasure trove of distinctive electrophilic functional groups, including chloroenone, α-chloroketone, epoxyketone, lactol, and α,β,γ,δ-unsaturated amide moieties; indeed, some members contain as many as five potential electrophilic sites.^5^ While detailed target identification studies are lacking, many of these tyrosine-derived alkaloids are reported to possess significant anti-cancer activity.^4^

**Fig. 2:**
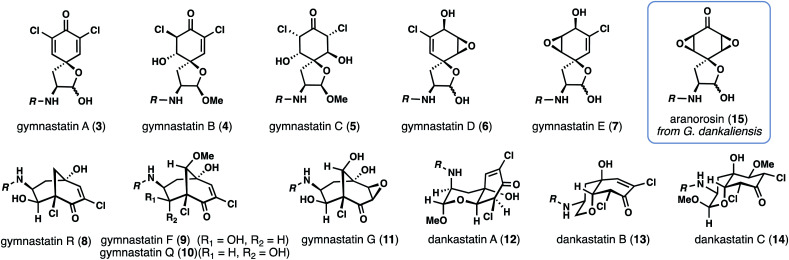
Selected chlorinated gymnastatin and dankastatin members and related natural product aranorosin.

With over 20 members isolated possessing varying degrees of oxygenation, halogenation, and cyclization, it is not unreasonable to suspect that gymnastatin A (**3**) plays a central role in the biosynthesis of other tyrosine-derived alkaloids (see **4–14**), possibly through chemistry which can be replicated without enzymes (Fig. 2). Indeed, biosynthetic logic has guided synthetic routes to various gymnastatin members and related alkaloids.^6,7^ Despite this, detailed chemical insight regarding the formation, stereochemistry, and interconversion of various members is lacking. Given our interest in bicyclo[3.3.1]nonane-containing natural products and covalently binding natural products, especially those containing similar lipophilic amide side chains,^3^ we sought to develop a unified synthetic platform to these alkaloids as a gateway into studying their biological targets.^8,9^ Herein we report simple synthetic solutions to multiple chlorinated gymnastatin and dankastatin metabolites, uncovering very subtle factors which favor the formation of a given skeletal type. We also provide an unappreciated link between this natural product family and the well-known fungal natural product aranorosin (**15**) which has also been isolated from a terrestrial variant of *G. dankaliensis*. Finally, we report growth inhibitory activity of six members spanning all three scaffold types (spirocyclic dienone, bicyclo[3.1.1]nonane, and oxo-decalin) against human triple negative breast cancer cells.

## Results and discussion

**Fig. 3:**
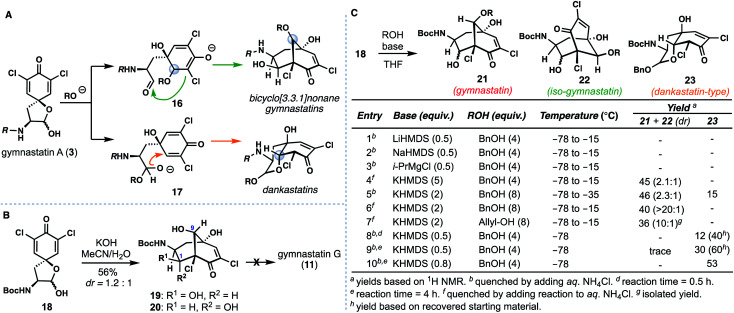
Understanding ring formation in the biosynthesis of gymnastatin and dankastatin alkaloids. (A) Chemical and stereochemical possibilities. (B) Stereochemical problems encountered when employing water as an oxygen nucleophile (C) optimization studies.

Bicyclo[3.3.1]nonane-containing gymnastatins and the oxo-decalin-containing dankastatins are proposed to arise from **3***via* aldol (see **16**) and oxa-Michael (see **17**) pathways respectively (Fig. 3A).^4^ The presence of a C-9 methyl ether in gymnastatins F (**9**) and Q (**10**) relative to a secondary hydroxyl group in gymnastatins G (**11**) raises questions regarding the identity of the “OR” group that can trigger this process (*i.e.* H_2_O *vs.* MeOH), in addition to stereochemical concerns arising from inter- *vs.* intramolecular delivery of the oxygen nucleophile. Additionally, dankastatins exists as two different sets of oxo-*cis*-decalin diastereomers (compare **12***vs.***13**/**14**); how (or if) nature controls the formation of a given isomer is an intriguing question.

We initially targeted gymnastatin G (**11**) owing to its potent reported activity against the P388 lymphocytic leukemia cell line, and reactive epoxyketone functionality.^4*a*^ Inspired by the work of Nishiyama on ether-containing gymnastatins F/Q, we had hoped that simply substituting methanol with water would forge the bicyclo[3.3.1]nonane core of **11** in a biomimetic cascade (Fig. 3B).^6*d*^ Known compound **18**, derived from (l)-tyrosine,^6^ was treated with aqueous KOH in MeCN yielding two bicyclo[3.3.1]nonane-containing products, **19** and **20** in a 1.2 : 1 ratio. Surprisingly, however, the C-9 stereocenter was incorrectly set during this process.^10^ Isomer **20** could be converted to **19** by treatment with catalytic amounts of base, suggesting that diastereomers at C-1 are formed in a reversible aldol reaction step, and that, the oxa-Michael addition step, albeit producing an undesired outcome, was stereoselective. Presumably this outcome arises from fast intramolecular oxa-Michael addition, wherein a hydrated aldehyde intermediate (see **17**, R = H) serves to deliver the oxygen nucleophile internally forming the *cis*-6,6-fused (dankastatin-type) bicyclic lactol. Subsequent lactol ring-opening then generates an aldehyde which participates in the aldol process. This observed reactivity questions the strategy nature employs in setting the correct C-9 stereocenter if water is used as a nucleophile. From a synthetic standpoint, we were also not successful in advancing **19**/**20** into gymnastatin G (**11**) (*vide infra*).^11^

Given these results, we examined alternative alcohol-based nucleophiles in order to prevent the proposed reaction pathway that leads to undesired C-9 stereochemistry; the resulting alkyl ethers formed could in principle be deprotected and ultimately processed to **11** which we desired for biological testing (Fig. 3C). Dienone **18** was reacted with various quantities of either allyl or benzyl alcohol using a variety of bases and subsequently quenched at various temperatures. Employing sub stoichiometric quantities of Li-, Mg-, and Na-based bases was ineffective at low temperatures (entries 1–3), but potassium bases employed in excess afforded substantial amounts of the desired bicyclo [3.3.1]nonane **21** and isomeric counterpart **22** (entries 4–7). The gymnastatin-type scaffold was favored under these conditions, and optimal ratios of **21** were obtained using two equivalents of base (entries 6 and 7).^12^ Of note, in entry 5, wherein the reaction was kept colder, we observed formation of small amounts of the dankastatin scaffold (see **23**) in addition to **21**/**22**. Finally, maintaining a −78 °C reaction temperature (entries 8–10) led to substantial quantities of **23** showing that under carefully controlled conditions either scaffold can be generated from **18**.

**Scheme 1:**
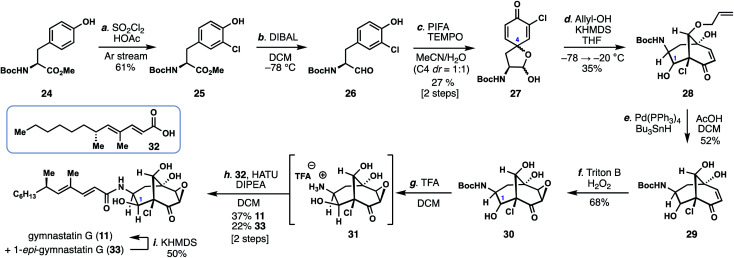
Total synthesis of gymnastatin G.

With conditions identified for construction of the key bicyclo[3.3.1]nonane core with the correct C-9 stereocenter, we reinvestigated the synthesis of gymnastatin G (**11**) (Scheme 1). While the epoxide found in **11** could be envisioned to arise from the chloroenone motif of gymnastatin F/Q, we had been unable to realize this process using previously prepared isomer **19**/**20**.^11^ Given these observations, we proceeded to investigate a monochlorinated tyrosine derivative as a means to synthesize **11**.

Carefully controlled mono-chlorination of Boc-tyrosine methyl ester (**24**) was achieved using sulfuryl chloride under a stream of argon, by which the produced HCl could be removed thus preventing undesired removal of the Boc group under acidic conditions. The resulting ester (**25**) was then reduced with DIBAL providing aldehyde **26**, which was subsequently dearomatized with PIFA in the presence of TEMPO to provide spirolactol **27** as a mixture of four diastereomers, two of which are inconsequential. Using conditions discovered previously (*vide supra*), treatment of **27** with allyl alcohol and KHMDS led to the bicyclo[3.3.1]nonane-containing product **28** in 35% yield as a mixture of diastereomers. Of note, the C-1 hydroxyl group, formed in the aldol step, was α-disposed in the major product. Moreover, no isomeric bicyclo[3.3.1]nonane-containing products possessing an α-chloroenone motif were formed. The carefully-chosen allyl protecting group was removed under reductive palladium-catalysis (Pd(PPh_3_)_4_, Bu_3_SnH) providing triol **29**. Diastereoselective epoxidation of **29** with H_2_O_2_ and Triton B afforded epoxyketone **30**; notably the basic conditions employed also partially epimerized the C-1 stereocenter (presumably *via* a retro-aldol/aldol reaction) to now favor β-stereochemistry as found in **11**. The mixture of epoxides were then exposed to TFA, removing the Boc group, and the free amine (**31**) coupled to known acid **32** (HATU, DIPEA) thus delivering gymnastatin G (**11**) and its C1-epimer (**33**) in 60% combined yield.^13^ Epimer **33** could also be converted into **11** in 50% yield by treatment with KHMDS.

**Fig. 4:**
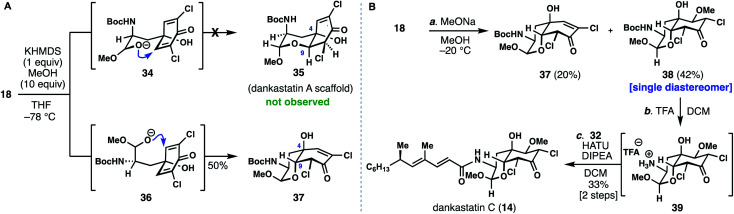
Studies towards the dankastatins. (A) Challenges in forming dankastatin A. (B) Total synthesis of dankastatin C.

With access to the most complex gymnastatin member secured for biological testing, we turned our attention toward the dankastatin family given that our initial screening of cyclization conditions turned up conditions to favor this scaffold. Chlorinated dankastatin members (see **12–14**), however, are produced with two different isomeric *cis*-decalin frameworks. Notably, in dankastatin A (**12**) the tertiary alcohol and neighboring proton (see C-4 and C-9) are on opposite faces as compared to dankastatins B (**13**) and C (**14**). In analogy to work in Fig. 3C, treating **18** with KHMDS/MeOH generated compound **37** and not the dankastatin A-type *cis*-fused skeleton **35** (Fig. 4A). We presume that in the cyclization of **18**, an axial configuration of the amide side chain (see **34***vs.***36**), disfavors formation of **35**.^14^ Again, this raises the question as to how the dankastatin A-type skeleton is prepared in nature. Fortunately, isomer **37** does however, bear resemblance to dankastatins B and C, thus offering a potential pathway to these targets (Fig. 4B).

Dankastatin C, a more recently isolated member of the dankastatin family,^4*c*^ possesses a structure suggestive of a hydration event on a biosynthetic intermediate akin to chloroenone **37**. In order to synthesize this structure, subtle adjustments were made to the conditions for the intramolecular oxa-Michael addition. Sodium methoxide was utilized as base with MeOH as solvent and the reaction mixture maintained at −20 °C for a prolonged period—long enough for the double MeOH adduct (**38**) to be the major product but without significant bicyclo[3.3.1]nonane formation. If KHMDS was used as base, bicyclo[3.3.1]nonane-containing products predominated. Through this process, **38** was formed as a single diastereomer in 42% yield along with 20% of **37**. Finally, Boc deprotection of **38** with TFA, followed by coupling with side chain **32** (HATU, DIPEA) forged dankastatin C (**14**).

**Fig. 5:**
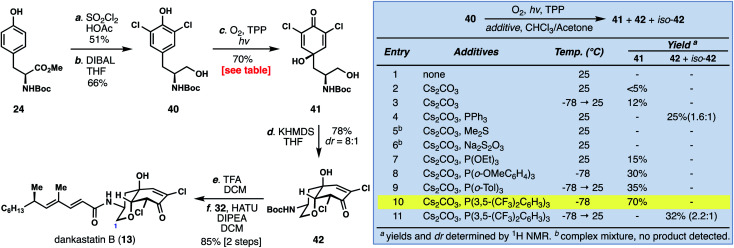
Total synthesis of dankastatin B.

Unlike dankastatin C, and in fact the majority of other tyrosine-derived alkaloids from *Gymnascella*, dankastatin B (**14**) features an alcohol, rather than aldehyde, oxidation state at C-1. To access this natural product, Boc-tyrosine methyl ester (**24**) was dichlorinated (SOCl_2_, HOAc) and reduced with DIBAL to yield **40** (Fig. 5). With **40** in hand, we sought to find suitable oxidative dearomatization conditions that were compatible with the free hydroxy group. After some exploration, success was realized using singlet oxygen-based conditions (O_2_, TPP, *hν*) in the presence of cesium carbonate (see inset). The yields of this process were initially quite low (entries 1–3), but in the presence of PPh_3_ the dankastatin core (see **42**) could be formed directly, albeit in low yield (entry 4). Interestingly, in addition to **42**, we observed a minor isomer (iso-**42**) which corresponds to the dankastatin A scaffold (dr ∼ 1.6 : 1). Through reductant and temperature optimization (entries 5–11), we found that high yields of dienone **41** (70%) could be obtained using an electron-deficient phosphine (P(3,5-(CF_3_)_2_C_6_H_3_)_3_) at low temperature. Isolated **41** could then be converted to **42** under basic conditions (KHMDS) in 78% yield. While this sequence requires two steps, the yield (78%) and diastereoselectivity (dr = 8 : 1) were substantially higher than the one-pot transformation. Deprotection of **42** (TFA) and coupling with acid **32** yield dankastatin B (**13**).

The successful application of proposed biomimetic strategies in the synthesis of dankastatin and gymnastatin alkaloids sheds some light on how nature might make these natural products and the challenges it faces and/or solves in doing so. Yet problems we encountered in our pursuit of gymnastatin G and dankastatin A led us to consider alternative hypotheses for the origins of these chlorinated alkaloids derived from tyrosine. Specifically, we were drawn to the bis-epoxyketone-containing natural product aranorosin (**15**), which is not halogenated, but bears clear structural, and likely biosynthetic, similarities to **3–14**.^4*d*^ Notably, the α-chloroenone in gymnastatin A and the epoxyketone in aranorosin are of the same oxidation level and we wondered if nature might use nucleophilic, chloride-mediated chemistry and not electrophilic chloronium-induced reactions in the construction of this alkaloid family.^15,16^

**Fig. 6:**
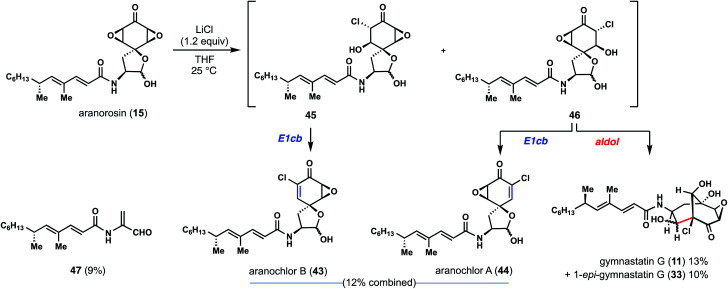
Aranorosin as a possible biosynthetic precursor to chlorinated alkaloids from *Gymnascella* sp.

Commercially available aranorosin reacted with LiCl (1.5 equiv.) at room temperature in THF, forming a variety of chlorinated products under very mild conditions (Fig. 6).^17^ Notably, gymnastatin G (**11**) and 1-*epi*-gymnastatin G (**33**) were isolated from the reaction mixture in 23% combined yield, presumably through an aldol reaction of intermediate **46**. In addition, two more natural products, namely aranochlor A (**44**) and aranochlor B (**43**), which are oxidized variants of gymnastatins D (**6**) and E (**7**) respectively, were also formed in the reaction (in 12%) and can be viewed as links between gymnastatin A and aranorosin.^18^ Notably, the two diastereomeric natural products (presumably generated *via* E1cB reactions of **45** and **46**) were generated in nearly a 1 : 1 ratio—an apparent result of nonselective epoxide opening; this observation echoes back to the two diastereomeric skeletons of dankastatins found in nature. Also detected was a small amount of unsaturated aldehyde **47**, a structure reminiscent of prior C–C cleavage products.^12^ While we are unaware of **47** being a real natural product, it is interesting to consider whether *Gymnascella dankaliensis* might employ oxidized tyrosines as precursors to electrophilic small molecules containing dehydroalanine-like motifs. In any event, investigations into the enzymology surrounding gymnastatin and dankastatin alkaloid biosynthesis is appealing.

**Fig. 7:**
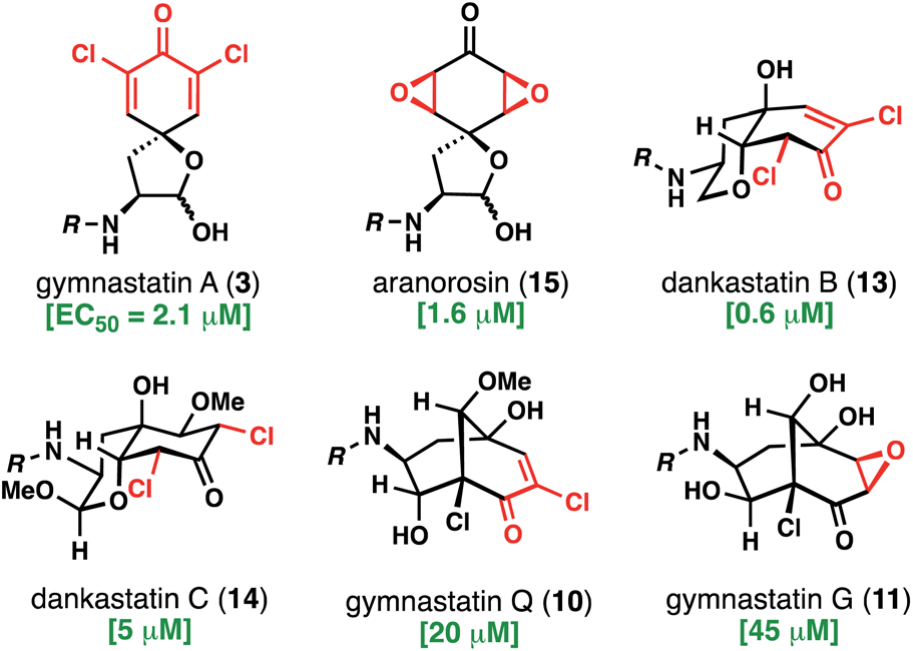
Anti-triple negative breast cancer (231 MFP) activity of select electrophilic alkaloids from *Gymnascella* sp.

With access to these natural products, which contain all of the relevant structural types found in this family, we evaluated their cytotoxicity against aggressive human triple negative breast cancer cells (231MFP cell line) (Fig. 7).^19^ As noted, many chlorinated tyrosine-derived alkaloids have shown strong anticancer activity, although many of these studies have been conducted in murine tumor cell lines.^4^ Dankastatin B exhibited the highest potency (EC_50_ = 0.6 μM) followed closely by aranorosin (EC_50_ = 1.6 μM), gymnastatin A (EC_50_ = 2.1 μM), and finally dankastatin C (EC_50_ = 5 μM). Interestingly, bicyclo[3.3.1]nonane-containing gymnastatins Q and G, which notably also contain only a single electrophilic site in the oxidized tyrosine core, were far less active in this cellular context. Whether these natural products have the same (or related) biological target profiles remains to be determined – work to address these questions is currently underway and will be reported in due course.

## Conclusion

In summary, we have completed the first total syntheses of gymnastatin G and dankastatins B and C, and in the process, explored stereochemical and structural questions surrounding the origins of chlorinated, tyrosine-derived alkaloids. During our studies, we discovered that very small and subtle changes to abiotic reaction conditions could be leveraged to promote the formation of either oxo-decalin or bicyclo[3.1.1]nonane motifs; how nature modulates these product ratios remains an open and interesting question. Additionally, an alternative biosynthetic hypothesis for the origins of chlorinated gymnastatin alkaloids from the well-known fungal metabolite aranorosin was also presented; notably this pathway can circumvent certain stereochemical problems associated with the abiotic Michael/aldol cascade approach. Finally, as a result of these investigations, dankastatin B has emerged as a potent, and easily synthesized, small molecule hit against triple negative breast cancer.

## Author contributions

T. J. M. and B. T. initiated the project and B. T. conducted all of the synthetic experiments. B. P. B. and D. K. N. performed biological assays. T. J. M. wrote the paper with the assistance of B. T. All authors provided feedback and contributed to editing the manuscript.

## Conflicts of interest

This study was funded in part by the Novartis Institutes for BioMedical Research and the Novartis-Berkeley Center for Proteomics and Chemistry Technologies. Daniel K. Nomura is a co-founder, shareholder, and adviser for Frontier Medicines.

## Supplementary Material

SC-012-D1SC02613E-s001
