# Supplementary material for: Chemical investigations into the biosynthesis of the gymnastatin and dankastatin alkaloids
Source: Chem Sci. 2021 May 31;12(25):8884–91. doi: 10.1039/d1sc02613e (PMC8246081; doi:10.1039/d1sc02613e)
Supplement: SC-012-D1SC02613E-s001 [file SC-012-D1SC02613E-s001.pdf]

Electronic Supplementary Material (ESI) for Chemical Science.
This journal is © The Royal Society of Chemistry 2021

Chemical Investigations Into the Biosynthesis of the Gymnastatin and Dankastatin Alkaloids

Bingqi Tong, Bridget P. Belcher, Daniel K. Nomura, Thomas J. Maimone

Supplementary Information

Table of Contents

General Procedures……………………………..……………..……………………SI-3

Triol **19** and **20** ……………………………………………………………………...SI-4

General procedure for the screening of oxa-Michael addition/aldol reaction conditions……...SI-4

Bicycle **SI-1**……………………………………………………………….…….SI-4

Bicycle **SI-2**……………………………………………………………….….SI-5

Bicycle **SI-3**……………………………………………………………………..SI-5

Bicycle **SI-4**……………………………………………………………………..SI-5

Acetal **23**……………………………………………………………………….....SI-6

3'-Chloro-*N*-Boc-L-tyrosine methyl ester (**25**)……………………………………...SI-6

Aldehyde **26**…………………………………………………………………………SI-6

Lactol **27**………………………………………………………………………………SI-7

Diol **28**…………………………………………………………………………….…SI-7

Triol **29**…………………………………………………………………………………SI-8

Epoxide **30**……………………………………………………………………….......SI-9

Gymnastatin G (**11**)…………………………………………………………….……SI-9

1-*epi*-gymnastatin G (**33**)…………………………...SI-10

Gymnastatin G diacetate (**SI-5**)……………………………………………………....SI-10

Acetal **37**………………………………………………………………………...…SI-11

Acetal **38**……………………………………………………………………………...SI-11

Dankastatin C (**14**)……………………………………………………………………SI-12

3'5'-Dichloro-N-Boc-L-tyrosine methyl ester (**SI-6**)……………………………….…..SI-12

Alcohol **40**………………………………………………………………………………SI-13

Diol **41**……………………………………………………………………………...…SI-13

Oxa-decalin **42** and *iso*-**42**………………………………………………........SI-14

Dankastatin B (**13**)……………………………………………………………………SI-14

Aranochlor A (**44**)……………………………………………………………...…...…SI-15

Aranochlor B (**43**)……………………………………………………………...……..SI-15

Spectra Comparison………………...…… ………………………………………………..SI-16

Gymnastatin G diacetate (**SI-5**)…………………………………………………..SI-16

Dankastatin B (**13**)………………………………………………………………..SI-17

Dankastatin C (**14**)………………………………………………………………..SI-18

Aranochlor A (**44**)……………………………………………………………..….SI-19

Aranochlor B (**43**)……………………………………………………………..….SI-20

Determination of optical purity using Mosher's ester/amide…………………………………SI-21

References…………………………………………………………………………...…..SI-28

^{1}H and ^{13}C NMR spectra……………………………………………………………...SI-29

2D-NOESY spectra for selected compounds…………………………………………………SI-89

General Procedures:

Unless otherwise noted, all reactions were performed in flame-dried glassware under positive pressure of nitrogen or argon. Air- and moisture-sensitive liquids were transferred via syringe. Dry tetrahydrofuran, dichloromethane, *N*,*N*-Dimethylformamide, toluene, acetonitrile, triethylamine and diethyl ether were obtained by passing these previously degassed solvents through activated alumina columns. Aranorosin was purchased from Cayman Chemicals. All reagents were used as received from commercial sources, unless stated otherwise. Reactions were monitored by thin layer chromatography (TLC) on TLC silica gel 60 F_{254} glass plates (EMD Millipore) and visualized by UV irradiation and staining with *p*-anisaldehyde, phosphomolybdic acid, or Ninhydrin. Volatile solvents were removed under reduced pressure using a rotary evaporator. Flash column chromatography was performed using Silicycle F60 silica gel (60Å, 230-400 mesh, 40-63 μm). Proton nuclear magnetic resonance (^{1}H NMR) and carbon nuclear magnetic resonance (^{13}C NMR) spectra were recorded on Bruker AV-600 and AV-700 spectrometers operating at 600 and 700 MHz for ^{1}H NMR, and 151 and 176 MHz for ^{13}C NMR. Chemical shifts are reported in parts per million (ppm) with respect to the residual solvent signal $CDCl_3$ (^{1}H NMR: δ 7.26; ^{13}C NMR: δ 77.16), $(CD_3)_2CO$ (^{1}H NMR: δ 2.05; ^{13}C NMR: δ 29.84). Peak multiplicities are reported as follows: *s* = singlet, *d* = doublet, *t* = triplet, *dd* = doublet of doublets, *tt* = triplet of triplets, *m* = multiplet, *br* = broad signal. IR spectra were recorded on a Bruker Vertex 80 FTIR spectrometer. High-resolution mass spectra (HRMS) were obtained by the QB3/chemistry mass spectrometry facility at the University of California, Berkeley using a Thermo LTQ-FT mass spectrometer with electrospray ionization (ESI) technique. X-ray crystallographic analysis was performed by the X-ray crystallography facility at the University of California, Berkeley on a Bruker APEX-II diffractometer with Mo-Kα radiation (λ = 0.71073 Å) or a MicroStar-H X8 APEX-II diffractometer with Cu-Kα radiation (λ = 1.54178 Å). Optical rotations were obtained using Perkin-Elmer 241 Polarimeter.

Cell proliferation assays were performed using Hoechst 33342 dye (Invitrogen) as previously described.[1] 231MFP cells were seeded at 20,000 cells/well in 150 μL of serum-containing L15 media (HyClone) in 96-well plates and allowed to adhere overnight. The cells were then treated with 50 μL of media containing DMSO vehicle or a 1:250 dilution of 1,000x alkaloid natural product stock. The cells were incubated for 24 or 48 h at 37°C and 0% CO_2 before being fixed and stained with 100 μL fixing solution with 10% formalin and Hoechst 33342 dye. The cells were incubated for 15 min in the dark at room temperature, and the fixing solution was removed and the fixed cells were washed with PBS prior to imaging. Fluorescence was read with a Tecan Spark plate reader (λex = 350 nm, λem = 461 nm).

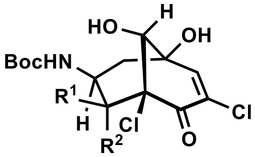

19: R[1] = OH, R[2] = H
20: R[1] = H, R[2] = OH

Triol **20** and **19**: To a 30 mL reaction tube was added compound **18** (56.5 mg, 0.107 mmol, 1.0 equiv) and MeCN (2.4 mL). The resulting solution was cooled to 0 °C and aqueous KOH (6.0 mg in 0.6 mL H_2O, 0.107 mmol, 1.0 equiv) was added. The reaction mixture was stirred at 0 °C for 30 min. The reaction was then quenched with saturated *aq*. NH_4Cl, extracted with DCM (2×2 mL) and EtOAc (2×2 mL), dried over Na_2SO_4 and concentrated *in vacuo*. The residue was purified by column chromatography (DCM/acetone = 3:1) to afford compound **19** (15.2 mg, 26% yield) and compound **20** (17.9 mg, 30% yield), both as a white solid. **19**: ^{1}H NMR (600 MHz, Acetone) δ 7.37 (s, 1H), 6.02 (d, *J* = 8.6 Hz, 1H), 5.93 (d, *J* = 7.2 Hz, 1H), 5.48 (d, *J* = 8.1 Hz, 1H), 5.15 (s, 1H), 4.07 (d, *J* = 7.4 Hz, 1H), 3.74 (d, *J* = 8.3 Hz, 1H), 3.49 (td, *J* = 8.3, 4.2 Hz, 1H), 2.30 (t, *J* = 12.7 Hz, 1H), 1.83 (dd, *J* = 13.1, 4.9 Hz, 1H), 1.37 (s, 9H). ^{13}C NMR (151 MHz, Acetone) δ 186.1, 155.7, 153.0, 130.7, 79.8, 79.4, 77.8, 75.0, 74.4, 49.3, 29.5, 28.5. IR (thin film) ν_{max} (cm^{-1}) 3398, 2955, 2923, 2851, 1709, 1518, 1460, 1369, 1293, 1249, 1164, 1084. HRMS (ESI) *calcd.* for $[C_{14}H_{19}O_6NCl_2Na]^+$ ($[M+Na]^+$): *m/z* 390.0482, found: 390.0482. **20**: ^{1}H NMR (600 MHz, Acetone) δ 7.29 (s, 1H), 6.16 (brs, 1H), 5.64 (d, *J* = 4.9 Hz, 1H), 4.98 (s, 1H), 4.50 (d, *J* = 5.0 Hz, 1H), 4.17 (dd, *J* = 10.5, 5.1 Hz, 1H), 4.04 (d, *J* = 4.6 Hz, 1H), 3.44 (m, 1H), 2.37 (t, *J* = 12.4 Hz, 1H), 1.98 (dd, *J* = 12.7, 5.4 Hz, 1H), 1.38 (s, 9H). ^{13}C NMR (151 MHz, Acetone) δ 184.0, 156.6, 151.5, 131.8, 83.5, 79.2, 79.1, 74.9, 74.5, 51.6, 35.2, 28.6. IR (thin film) ν_{max} (cm^{-1}) 3365, 2957, 2921, 2850, 1701, 1685, 1655, 1522, 1457, 1368, 1297, 1249, 1163, 1101, 946. HRMS (ESI) *calcd.* for $[C_{14}H_{19}O_6NCl_2Na]^+$ ($[M+Na]^+$): *m/z* 390.0482, found: 390.0482.

General procedure for the screening of *oxa*-Michael addition/aldol reaction conditions (Figure 3C):

To a 10 mL reaction tube was added compound **18**, benzyl alcohol or allyl alcohol, and THF. The resulting solution was cooled to –78 °C and base was added dropwise. The reaction was allowed to stir at –78 °C or warm up slowly to the specified temperature over 30 – 40 min. Upon reaching the designated temperature, the reaction was quenched by either adding saturated *aq.* NH_4Cl to the reaction mixture, or adding the reaction to a stirring saturated *aq.* NH_4Cl using pipette. The layers were then separated, and the aqueous phase was extracted with Et_2O (2×1 mL) and DCM (2×1 mL). The combined organic layer was dried over Na_2SO_4 and concentrated *in vacuo*. The yields were determined by ^{1}H NMR using 1,3,5-trimethoxybenzene as internal standard.

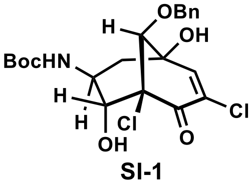

SI-1

Bicycle **SI-1**: ^{1}H NMR (700 MHz, Acetone) δ 7.36 – 7.33 (m, 2H), 7.32 – 7.28 (m, 2H), 7.28 – 7.24 (m, 1H), 7.06 (d, *J* = 2.5 Hz, 1H), 6.21 (s, 1H), 5.42 (d, *J* = 1.2 Hz, 1H), 5.05 (d, *J* = 4.9 Hz, 1H), 4.95 (d, *J* = 10.9 Hz, 1H), 4.89 (d, *J* = 10.9 Hz, 1H), 4.12 (dd, *J* = 10.3, 5.0 Hz, 1H), 4.04 (d, *J* = 2.5 Hz, 1H), 3.58 (dddd, *J* = 12.3, 10.3, 8.2, 5.5 Hz, 1H), 2.23 (dd, *J* = 13.2, 5.5 Hz, 1H), 2.10 (t, *J* = 13.3 Hz, 1H), 1.39 (s, 9H). ^{13}C NMR (151 MHz, Acetone) δ 182.7, 156.6, 148.8, 139.2, 131.4, 128.9, 128.7, 128.4, 91.1, 83.9, 79.3, 78.7, 76.9, 75.1, 51.8, 38.5, 28.6. IR (thin film) ν_{max} (cm^{-1}) 3383, 2922, 2852, 1709, 1516, 1454, 1368, 1311, 1294,

1253, 1164, 1098, 1060. 967, 748, 700. HRMS (ESI) *calcd.* for $[C_{21}H_{25}O_6NCl_2Na]^+$ ($[M+Na]^+$): *m/z* 480.0951, found: 480.0957.

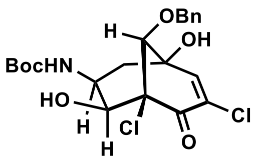

SI-2

Bicycle **SI-2**: ^{1}H NMR (600 MHz, Acetone) δ 7.37 – 7.33 (m, 2H), 7.33 – 7.28 (m, 2H), 7.28 – 7.23 (m, 1H), 7.11 (d, *J* = 2.4 Hz, 1H), 5.99 (d, *J* = 8.6 Hz, 1H), 5.65 (d, *J* = 5.0 Hz, 1H), 5.37 (s, 1H), 4.92 (d, *J* = 10.9 Hz, 1H), 4.83 (d, *J* = 10.9 Hz, 1H), 4.29 (d, *J* = 2.5 Hz, 1H), 3.91 (ddd, *J* = 4.7, 3.0, 1.3 Hz, 1H), 3.62 (m, 1H), 2.26 (t, *J* = 12.6 Hz, 1H), 1.97 (dd, *J* = 12.6, 3.9 Hz, 1H), 1.37 (s, 9H). ^{13}C NMR (151 MHz, Acetone) δ 185.5, 155.7, 150.1, 139.3, 130.5, 128.9, 128.6, 128.4, 87.8, 82.7, 79.4, 77.0, 75.6, 74.3, 48.8, 35.5, 28.5. IR (thin film) v_{max} (cm^{-1}) 3394, 2955, 2923, 2852, 1709, 1509, 1456, 1392, 1368, 1310, 1295, 1251, 1164, 1095, 968, 738, 701. HRMS (ESI) *calcd.* for $[C_{21}H_{25}O_6NCl_2Na]^+$ ($[M+Na]^+$): *m/z* 480.0951, found: 480.0955.

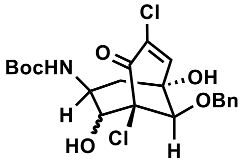

SI-3

Bicycle **SI-3**: ^{1}H NMR (600 MHz, Acetone) major diastereomer δ 7.35 (td, *J* = 8.3, 1.4 Hz, 2H), 7.30 (td, *J* = 7.4, 1.5 Hz, 2H), 7.28 – 7.23 (m, 1H), 7.10 (d, *J* = 2.4 Hz, 1H), 5.53 (d, *J* = 5.0 Hz, 1H), 5.28 (s, 1H), 4.92 (d, *J* = 10.9 Hz, 1H), 4.85 (d, *J* = 10.9 Hz, 1H), 4.39 (d, *J* = 2.4 Hz, 1H), 4.36 – 4.31 (m, 1H), 3.96 (dt, *J* = 6.1, 2.0 Hz, 1H), 2.53 (dd, *J* = 14.6, 6.5 Hz, 1H), 2.35 (dt, *J* = 14.6, 1.5 Hz, 1H), 1.34 (s, 9H); Minor diastereomer δ 7.35 (td, *J* = 8.3, 1.4 Hz, 2H), 7.30 (td, *J* = 7.4, 1.5 Hz, 2H), 7.28 – 7.23 (m, 1H), 6.99 (d, *J* = 2.5 Hz, 1H), 5.45 (d, *J* = 4.7 Hz, 1H), 5.28 (s, 1H), 4.95 (d, *J* = 10.9 Hz, 1H), 4.89 (d, *J* = 10.9 Hz, 1H), 4.52 (dd, *J* = 6.0, 4.7 Hz, 1H), 4.21 (tdd, *J* = 5.9, 4.0, 2.6 Hz, 1H), 4.14 (d, *J* = 2.5 Hz, 1H), 2.86 (d, *J* = 14.4 Hz, 1H), 2.22 (dd, *J* = 14.7, 5.6 Hz, 1H), 1.38 (s, 9H). ^{13}C NMR (151 MHz, Acetone) (all peaks listed) δ 185.1, 183.4, 156.5, 156.0, 150.5, 149.4, 139.4, 139.2, 131.6, 131.0, 128.94, 128.93, 128.66, 128.60, 128.40, 128.33, 90.8, 88.0, 82.8, 82.6, 79.7, 79.6, 76.95, 76.94, 76.81, 76.1, 75.7, 74.8, 53.0, 50.5, 37.0, 36.2, 28.52, 28.48. IR (thin film) v_{max} (cm^{-1}) 3374, 2957, 2922, 2851, 1707, 1495, 1455, 1368, 1258, 1163, 1091, 1026, 968, 732, 700. HRMS (ESI) *calcd.* for [C21H25O6NCl$_2$Na]$^+$ ($[M+Na]^+$): *m/z* 480.0951, found: 480.0954.

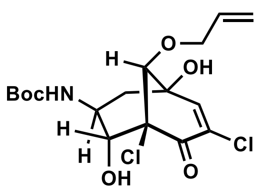

SI-4

Bicycle **SI-4**: ^{1}H NMR (600 MHz, Acetone) δ 7.03 (d, *J* = 2.5 Hz, 1H), 6.16 (s, 1H), 5.93 (dddd, *J* = 16.7, 9.7, 6.2, 5.3 Hz, 1H), 5.28 (s, 1H), 5.20 (d, *J* = 17.4 Hz, 1H), 5.08 (d, *J* = 10.4 Hz, 1H), 4.98 (d, *J* = 4.9 Hz, 1H), 4.40 (dd, *J* = 12.4, 5.9 Hz, 1H), 4.33 (dd, *J* = 12.0, 5.4 Hz, 1H), 4.07 (dd, *J* = 10.4, 4.7 Hz, 1H), 3.87 (d, *J* = 2.5 Hz, 1H), 3.55 (dddd, *J* = 12.9, 10.3, 8.1, 5.5 Hz, 1H), 2.20 (dd, *J* = 13.2, 5.5 Hz, 1H), 2.02 (t, *J* = 12.8 Hz, 1H), 1.38 (s, 9H). ^{13}C NMR (151 MHz, Acetone) δ 182.6, 156.5, 148.9, 135.8, 131.3, 117.2, 90.7, 83.7, 79.3, 78.7, 76.0, 74.8, 51.8, 38.3, 28.6. IR (thin film) v_{max} (cm^{-1}) 3387, 2956, 2925, 2854, 1709, 1520, 1456, 1393, 1386, 1311, 1294, 1252, 1164, 1095, 1061, 777, 754. HRMS (ESI) *calcd.* for $[C_{17}H_{23}O_6NCl_2Na]^+$ ($[M+Na]^+$): *m/z* 430.0795, found: 430.080. Other minor diastereomers were assigned based on the similarity of the ^{1}H-NMR compared with Bn analogues.

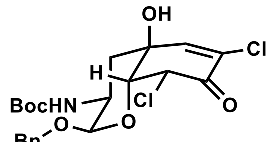
23

Acetal **23**: ^{1}H NMR (600 MHz, Acetone) δ 7.41 (d, *J* = 7.0 Hz, 2H), 7.37 (t, *J* = 7.6 Hz, 2H), 7.31 (t, *J* = 7.2 Hz, 1H), 7.10 (d, *J* = 2.3 Hz, 1H), 5.99 (d, *J* = 8.5 Hz, 1H), 5.57 (d, *J* = 2.4 Hz, 1H), 5.42 (s, 1H), 4.91 (d, *J* = 3.4 Hz, 1H), 4.83 (d, *J* = 11.9 Hz, 1H), 4.59 (d, *J* = 11.9 Hz, 1H), 4.35 (t, *J* = 2.4 Hz, 1H), 3.66 (tt, *J* = 8.7, 4.1 Hz, 1H), 2.25 – 2.17 (m, 2H), 1.38 (s, 9H). ^{13}C NMR (151 MHz, Acetone) δ 183.7, 155.9, 144.9, 138.4, 132.9, 129.3, 128.8, 128.6, 96.1, 79.3, 76.9, 70.3, 69.6, 62.3, 48.7, 38.4, 28.6. IR (thin film) v_{max} (cm^{-1}) 3373, 2957, 2921, 2851, 1691, 1506, 1457, 1368, 1259, 1163, 1095, 1022, 798, 738, 701. HRMS (ESI) *calcd.* for $[C_{21}H_{25}O_6NCl_2Na]^+$ ($[M+Na]^+$): *m/z* 480.0951, found: 480.0954.

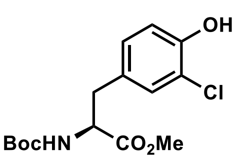
25

3'-Chloro-*N*-Boc-L-tyrosine methyl ester (**25**): To a 25 mL round-bottom flask was added *L*-Boc-Tyr-OMe (**24**) (500 mg, 1.69 mmol, 1.0 equiv) and acetic acid (13 mL). A stream of argon was gently bubbled through the solution and to the solution was added dropwise SO_2Cl_2 (0.21 mL, 2.54 mmol, 1.5 equiv). After stirring for 30 minutes, another portion of SO_2Cl_2 (0.21 mL, 2.54 mmol, 1.5 equiv) was added and the reaction was continued for another 30 minutes. The reaction was then poured into a mixture of Et_2O (50 mL) and saturated *aq.* $NaHCO_3$ (50 mL) with vigorous stirring. The layers were separated, and the aqueous phase was extracted with Et_2O (3×30 mL). The combined organic layer was concentrated *in vacuo*, redissolved in DCM (50 mL) and washed with saturated *aq.* $NaHCO_3$ (50 mL). The organic layer was then dried over Na_2SO_4 and concentrated *in vacuo*. The residue was purified by column chromatography (hexanes/EtOAc = 3:1) to afford ester **25** (342 mg, 61% yield) as a yellow oil. $[\alpha]_D^{20}$ +49.9 (*c* 1.0, $CHCl_3$). ^{1}H NMR (600 MHz, $CDCl_3$) δ 7.08 (s, 1H), 6.92 (m, 2H), 5.64 (s, 1H), 5.01 (d, *J* = 8.2 Hz, 1H), 4.59 – 4.47 (m, 1H), 3.72 (s, 3H), 3.04 (dd, *J* = 13.8, 6.0 Hz, 1H), 2.95 (dd, *J* = 14.1, 6.3 Hz, 1H), 1.42 (s, 9H). ^{13}C NMR (151 MHz, $CDCl_3$) δ 172.3, 155.2, 150.6, 130.6, 129.8, 129.4, 119.9, 116.4, 80.3, 54.6, 52.4, 37.5, 28.4. IR (thin film) v_{max} (cm^{-1}) 3365, 2978, 2955, 2928, 1739, 1691, 1505, 1439, 1367, 1293, 1254, 1221, 1165, 1057, 1020. HRMS (ESI) *calcd.* for $[C_{15}H_{20}O_5NClNa]^+$ ($[M+Na]^+$): *m/z* 352.0922, found: 352.0923.

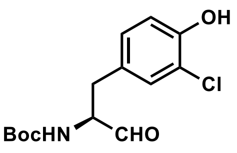
26

Aldehyde **26**: To a 100 mL round-bottom flask was added ester **25** (685 mg, 2.08 mmol, 1.0 equiv) and DCM (30 mL). The reaction was cooled to –78 °C and DIBAL (1.0 M in hexanes, 6.2 mL, 6.2 mmol. 3.0 equiv) was added very slowly. The reaction was allowed to stir at –78 °C for 1 h, after which it was quenched by the addition of MeOH (1 mL) at –78 °C followed by saturated *aq.* potassium sodium tartrate (20 mL). The reaction mixture was warmed to room temperature and stirred for another 2 h. The layers were then separated, and the aqueous phase was extracted with DCM (3×30 mL). The combined organic layer was dried over Na_2SO_4 and concentrated *in vacuo*. The resulting yellow oil was further dried under high vacuum for 3 h to provide a foam-like solid. Hexanes (5 mL) was added to the solid, stirred for 10 min, and carefully removed with pipette. This process was repeated for two more times. The resulting solid was dried in vacuo to afford crude aldehyde **26** (520 mg, 83%) which was used for next step without further purification. Pure aldehyde **26** can be obtained by column chromatography

(hexanes/EtOAc = 2:1) but partial racemization may occur during the process. $[\alpha]_D^{20}$ +11.2 (*c* 0.42, $CHCl_3$). ^{1}H NMR (600 MHz, $CDCl_3$) δ 9.63 (s, 1H), 7.14 (d, *J* = 1.9 Hz, 1H), 6.98 (dd, *J* = 8.3, 2.0 Hz, 1H), 6.95 (d, *J* = 8.3 Hz, 1H), 5.48 (s, 1H), 5.04 (brs, 1H), 4.37 (m, 1H), 3.08 (dd, *J* = 14.3, 6.5 Hz, 1H), 3.02 (dd, *J* = 14.4, 6.5 Hz, 1H), 1.44 (s, 9H). ^{13}C NMR (151 MHz, $CDCl_3$) δ 199.1, 155.5, 150.6, 129.9, 129.5, 129.1, 120.1, 116.6, 80.6, 60.9, 34.4, 28.4. IR (thin film) ν_{max} (cm^{-1}) 3361, 2954, 2922, 2851, 1687, 1504, 1457, 1369, 1292, 1255, 1165, 822. HRMS (ESI) *calcd.* for $[C_{14}H_{17}O_4NCl]^-$ ($[M-H]^-$): *m/z* 298.0852, found: 298.0857.

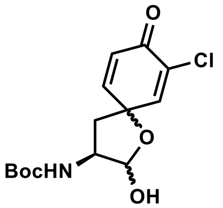

27

Lactol **27**: A 100 mL round-bottom flask was charged with aldehyde **26** (520 mg, 1.73 mmol, 1.0 equiv), TEMPO (136 mg, 0.867 mmol, 0.5 equiv) and MeCN/H_2O (5:1, 30 mL). The solution was cooled to 0 °C and PIFA (709 mg, 1.65 mmol, 0.95 equiv) was added in one portion. After stirring at 0 °C for 10 min, H_2O (10 mL) and DCM (30 mL) was added to the reaction. The layers were separated, and the aqueous phase was extracted with DCM (3×30 mL). The combined organic layer was dried over Na_2SO_4 and concentrated *in vacuo*. The residue was purified by column chromatography (hexanes/EtOAc = 2:1) to afford lactol **27** (182 mg, 33%, mixture of 4 diastereomers) as a pale-yellow foam. $[\alpha]_D^{20}$ –8.3 (*c* 0.24, $CHCl_3$). One of the two diastereomers at C-4: ^{1}H NMR (700 MHz, $CDCl_3$) δ 7.17 (d, *J* = 2.8 Hz, 0.36H), 7.08 (d, *J* = 2.8 Hz, 0.64H), 6.82 (dd, *J* = 9.8, 2.8 Hz, 0.36H), 6.79 (dd, *J* = 9.8, 2.8 Hz, 0.64H), 6.25 (d, *J* = 9.8 Hz, 0.36H), 6.22 (d, *J* = 9.8 Hz, 0.64H), 5.57 (brs, 0.36H), 5.50 (brs, 0.64H), 5.02 (brs, 0.64 H), 4.65 (brs, 0.36H), 4.45 (brs, 0.64H), 4.29 (brs, 0.36H), 3.22 (d, *J* = 16.1 Hz, 0.64H), 3.10 (d, *J* = 19.6 Hz, 0.36H), 2.72 (dd, *J* = 14.7, 7.0 Hz, 0.36H), 2.47 (m, 0.64H), 2.17 (dd, *J* = 14.7, 9.8 Hz, 0.64H), 2.11 (t, *J* = 14.7 Hz, 0.36H), 1.46 (s, 9H). The other diastereomer at C-4: ^{1}H NMR (700 MHz, $CDCl_3$) δ 7.00 (m, 0.36H), 6.97 (d, *J* = 2.8 Hz, 0.36H), 6.96 (d, *J* = 2.8 Hz, 0.64H), 6.90 (dd, *J* = 9.8, 2.8 Hz, 0.64H), 6.24 (m, 1H), 5.57 (brs, 0.36H), 5.50 (brs, 0.64H), 5.02 (brs, 0.64 H), 4.65 (brs, 0.36H), 4.45 (brs, 0.64H), 4.29 (brs, 0.36H), 2.69 (dd, *J* = 14.7, 7.0 Hz, 0.36H), 2.47 (m, 0.64H), 2.17 (dd, *J* = 14.7, 9.8 Hz, 0.64H), 2.11 (t, *J* = 14.7 Hz, 0.36H), 1.46 (s, 9H). ^{13}C NMR (151 MHz, $CDCl_3$) (peaks for all the diastereomers are listed) δ 178.40, 178.38, 155.36, 155.25, 152.0, 151.3, 149.4, 148.6, 147.6, 146.9, 145.1, 144.3, 132.0, 131.8, 131.4, 126.6, 126.4, 126.1, 103.53, 103.48, 96.42, 96.36, 80.54, 80.50, 78.93, 78.86, 53.8, 38.5, 28.5. IR (thin film) ν_{max} (cm^{-1}) 3374, 2977, 2926, 2853, 1684, 1507, 1455, 1393, 1368, 1291, 1252, 1165, 1051, 1019, 863, 822, 777, 737. HRMS (ESI) *calcd.* for $[C_{14}H_{18}O_5NClNa]^+$ ($[M+Na]^+$): *m/z* 338.0766, found: 338.0768.

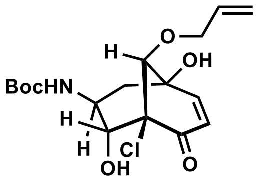

28a

Diol **28**: To a 50 mL reaction tube was added compound **27** (50.0 mg, 0.158 mmol, 1.0 equiv), THF (10 mL) and allyl alcohol (42 μL, 0.62 mmol, 4.0 equiv). The resulting solution was cooled to –78°C and KHMDS (0.5M in toluene, 1.6 mL, 0.79 mmol, 5.0 equiv) was added dropwise. The reaction was allowed to warm up slowly to –15°C over 40 min. The reaction was quenched by adding the reaction mixture to a stirring saturated *aq.* NH_4Cl using pipette. The layers were separated, and the aqueous phase was extracted with DCM (3×10 mL). The combined organic layer was dried over Na_2SO_4 and concentrated *in vacuo*. The residue was purified by column chromatography (45% EtOAc/hexanes) to afford diol **28a** along with a small amount of other diastereomers (**28b** and **28c**) as a pale-yellow

foam (20.5 mg, 35%). $[\alpha]_D^{20}$ +7.2 (*c* 0.25, acetone). ^{1}H NMR (600 MHz, Acetone) δ 6.76 (dd, *J* = 10.2, 2.6 Hz, 1H), 6.14 (d, *J* = 10.2 Hz, 1H), 6.10 (brs, 1H), 5.92 (ddt, *J* = 17.3, 10.4, 5.7 Hz, 1H), 5.19 (dq, *J* = 17.3, 1.7 Hz, 1H), 5.07 (dq, *J* = 10.5, 1.5 Hz, 1H), 4.97 (s, 1H), 4.64 (d, *J* = 4.7 Hz, 1H), 4.38 (ddt, *J* = 12.5, 5.7, 1.5 Hz, 1H), 4.32 (ddt, *J* = 12.6, 5.4, 1.5 Hz, 1H), 3.99 (dd, *J* = 10.3, 4.8 Hz, 1H), 3.79 (d, *J* = 2.6 Hz, 1H), 3.64 – 3.53 (m, 1H), 2.09 (dd, *J* = 13.1, 5.5 Hz, 1H), 1.96 (t, *J* = 13.2 Hz, 1H), 1.38 (s, 9H). ^{13}C NMR (151 MHz, Acetone) δ 188.8, 156.6, 152.5, 136.1, 129.3, 116.8, 91.2, 83.9, 79.1, 78.8, 75.8, 74.6, 51.9, 38.3, 28.6. IR (thin film) ν_{max} (cm^{-1}) 3380, 2957, 2925, 2854, 1688, 1524, 1456, 1368, 1297, 1251, 1164, 1086, 1060, 926, 806, 761. HRMS (ESI) *calcd.* for $[C_{17}H_{24}O_6NClNa]^+$ ($[M+Na]^+$): *m/z* 396.1184, found: 396.1183.

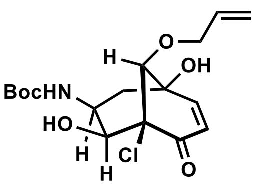

28b

28b: ^{1}H NMR (600 MHz, Acetone) δ 6.83 (dd, *J* = 10.1, 2.6 Hz, 1H), 6.16 (d, *J* = 10.1 Hz, 1H), 5.92 (ddt, *J* = 17.3, 10.4, 5.6 Hz, 1H), 5.87 (d, *J* = 8.6 Hz, 1H), 5.38 (d, *J* = 4.7 Hz, 1H), 5.19 (dt, *J* = 17.3, 1.8 Hz, 1H), 5.06 (dq, *J* = 10.4, 1.5 Hz, 1H), 4.97 (s, 1H), 4.34 (ddt, *J* = 12.5, 5.7, 1.5 Hz, 1H), 4.26 (ddt, *J* = 12.5, 5.6, 1.6 Hz, 1H), 4.11 (d, *J* = 2.6 Hz, 1H), 3.83 – 3.77 (m, 1H), 3.66 (td, *J* = 8.0, 3.9 Hz, 1H), 2.15 (t, *J* = 12.6 Hz, 1H), 1.82 (dd, *J* = 12.8, 4.9 Hz, 1H), 1.37 (s, 9H). ^{13}C NMR (151 MHz, Acetone) δ 191.6, 155.7, 153.7, 136.3, 128.5, 116.6, 87.8, 82.7, 79.2, 75.8, 75.0, 74.4, 48.8, 35.3, 28.5. IR (thin film) ν_{max} (cm^{-1}) 3390, 2956, 2922, 2851, 1692, 1498, 1458, 1369, 1298, 1258, 1163, 1085, 1043, 804, 700. HRMS (ESI) *calcd.* for $[C_{17}H_{24}O_6NClNa]^+$ ($[M+Na]^+$): *m/z* 396.1184, found: 396.1187.

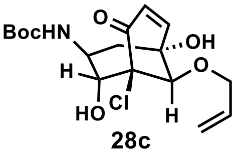

28c

28c: ^{1}H NMR (600 MHz, Acetone) δ 6.96 (dd, *J* = 10.1, 2.5 Hz, 1H), 6.08 (d, *J* = 10.1 Hz, 1H), 5.93 (ddt, *J* = 17.3, 10.5, 5.6 Hz, 1H), 5.35 (br s, 1H), 5.25 (d, *J* = 4.7 Hz, 1H), 5.19 (dq, *J* = 17.3, 1.8 Hz, 1H), 5.06 (dp, *J* = 10.4, 1.6 Hz, 1H), 4.90 (s, 1H), 4.34 (ddt, *J* = 12.5, 5.7, 1.5 Hz, 1H), 4.28 (ddt, *J* = 12.5, 5.5, 1.6 Hz, 1H), 4.20 (d, *J* = 2.5 Hz, 1H), 4.08 (dt, *J* = 4.2, 1.8 Hz, 1H), 4.02 – 3.97 (m, 1H), 2.46 (dd, *J* = 14.6, 6.9 Hz, 1H), 2.15 (dt, *J* = 14.6, 1.5 Hz, 1H), 1.33 (s, 9H). ^{13}C NMR (151 MHz, Acetone) δ 191.5, 155.6, 155.1, 136.3, 129.7, 116.5, 87.9, 82.7, 79.5, 76.4, 75.8, 75.4, 52.7, 36.5, 28.5. IR (thin film) ν_{max} (cm^{-1}) 3398, 2955, 2922, 2851, 1692, 1494, 1455, 1369, 1281, 1252, 1165, 1093, 1045, 1019, 794, 699. HRMS (ESI) *calcd.* for $[C_{17}H_{24}O_6NClNa]^+$ ($[M+Na]^+$): *m/z* 396.1184, found: 396.1188.

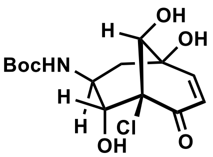

29

Triol **29**: To a 30 mL reaction tube was added diol **28** (64.4 mg, 0.172 mmol, 1.0 equiv), $Pd(PPh_3)_4$ (39.8 mg, 0.0345 mmol, 0.2 equiv) and DCM (5 mL). The reaction was cooled to 0 °C and AcOH (30 µL, 0.52 mmol, 3.0 equiv) was added, followed by dropwise addition of Bu_3SnH (55.6 µL, 0.21 mmol, 1.2 equiv). After stirring at 0 °C for 1 h, the reaction was quenched with saturated *aq.* KF (3 mL) and stirred vigorously for 2 h. The layers were then separated, and the aqueous phase was extracted with DCM (2×5 mL) and EtOAc (2×5 mL). The combined organic layer was dried over Na_2SO_4 and concentrated *in vacuo*. The residue was purified by column chromatography (5% MeOH/DCM) to afford triol **29** (30.1 mg, 52%) as a pale-yellow oil. $[\alpha]_D^{20}$ –3.3 (*c* 0.090, acetone). ^{1}H NMR (600 MHz, Acetone) δ 6.73 (dd, *J* = 10.2, 2.6 Hz, 1H), 6.15 (d, *J* = 10.2 Hz, 1H), 6.10 (brs, 1H), 5.10 (d, *J* = 4.8 Hz, 1H), 4.81 (s, 1H), 4.58 (d, *J* = 4.7 Hz, 1H),

3.99 – 3.94 (m, 2H), 3.63 (m, 1H), 2.11 (dd, *J* = 13.2, 5.4 Hz, 1H), 1.90 (t, *J* = 12.7 Hz, 1H), 1.38 (s, 9H). ^{13}C NMR (151 MHz, Acetone) δ 189.2, 156.6, 152.2, 129.4, 84.5, 83.2, 79.1, 78.7, 73.8, 52.0, 38.0, 28.6. IR (thin film) v_{max} (cm^{-1}) 3368, 2956, 2922, 2852, 1688, 1532,1460, 1370, 1298, 1251, 1165, 1085, 1046, 803. HRMS (ESI) *calcd.* for $[C_{14}H_{20}O_6NClNa]^+$ ($[M+Na]^+$): *m/z* 356.0871, found: 356.0872.

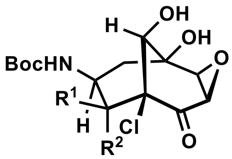

30a: R[1] = OH, R[2] = H
30b: R[1] = H, R[2] = OH

Epoxide **30**: To a 20 mL reaction tube was added triol **29** (40.2 mg, 0.121 mmol, 1.0 equiv) and THF (2 mL). The solution was cooled to 0 °C and Triton B (40%w/w, 14 µL, 0.036 mmol, 0.3 equiv) was added, followed by H_2O_2 (50% w/w, 136 µL, 2.4 mmol, 20 equiv). The reaction was stirred at 0 °C for 2 h before being quenched with saturated *aq.* $NaHSO_3$ (2 mL). The layers were then separated, and the aqueous phase was extracted with DCM (2×2 mL) and EtOAc (2×2 mL). The combined organic layer was dried over Na_2SO_4 and concentrated *in vacuo*. The residue was purified by column chromatography (5% MeOH/DCM) to afford epoxide **30a** and **30b** (28.4 mg, 68% combined) as a pale-yellow foam. **30b**: $[\alpha]_D^{20}$ –2.4 (*c* 0.17, acetone). ^{1}H NMR (600 MHz, Acetone) δ 5.94 (d, *J* = 8.3 Hz, 1H), 5.64 (d, *J* = 5.2 Hz, 1H), 5.13 (s, 1H), 4.12 (dd, *J* = 10.8, 2.3 Hz, 1H), 3.85 (d, *J* = 3.9 Hz, 1H), 3.82 (m, 1H), 3.80 (dd, *J* = 4.0, 2.3 Hz, 1H), 3.72 – 3.67 (m, 1H), 3.65 (d, *J* = 10.7 Hz, 1H), 2.14 (dd, *J* = 12.9, 4.5 Hz, 1H), 2.05 (1H, under solvent residue peak), 1.38 (s, 9H). ^{13}C NMR (151 MHz, Acetone) δ 199.2, 155.6, 80.9, 79.4, 76.4, 75.5, 70.6, 61.2, 56.0, 48.9, 36.5, 28.5. IR (thin film) v_{max} (cm^{-1}) 3369, 2957, 2922, 2851, 1731, 1690, 1520, 1458, 1393, 1368,1304, 1248, 1164, 1102, 1055, 926, 898, 869, 793. HRMS (ESI) *calcd.* for $[C_{14}H_{20}O_7NClNa]^+$ ($[M+Na]^+$): *m/z* 372.0821, found: 372.0821. **30a**: $[\alpha]_D^{20}$ +3.6 (*c* 0.14, acetone). ^{1}H NMR (600 MHz, Acetone) δ 6.13 (s, 1H), 5.07 (s, 1H), 4.97 (d, *J* = 4.9 Hz, 1H), 3.97 – 3.89 (m, 2H), 3.79 (dd, *J* = 9.0, 1.8 Hz, 1H), 3.78 – 3.73 (m, 1H), 3.73 – 3.70 (m, 2H), 2.39 (dd, *J* = 13.3, 5.5 Hz, 1H), 1.88 (t, *J* = 13.1 Hz, 1H), 1.40 (s, 9H). ^{13}C NMR (151 MHz, Acetone) δ 194.0, 156.5, 83.2, 80.1, 79.45, 79.34, 70.7, 61.0, 54.5, 52.0, 39.2, 28.6. IR (thin film) v_{max} (cm^{-1}) 3381, 2955, 2923, 2851, 1717, 1688, 1517, 1459, 1369, 1304, 1249, 1164, 1102, 1055, 1007, 870, 795. HRMS (ESI) *calcd.* for $[C_{14}H_{20}O_7NClNa]^+$ ($[M+Na]^+$): *m/z* 372.0821, found: 372.0821.

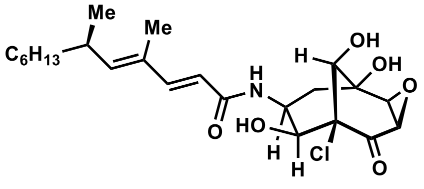

gymnastatin G (**11**)

Gymnastatin G (**11**) *i.* To a 10 mL reaction tube was added a mixture of epoxide **30** (18.9 mg, 0.0542 mmol, 1.0 equiv) and DCM (0.8 mL). Trifluoroacetic acid (0.16 mL) was added slowly at 0 °C. The reaction mixture was warmed up to room temperature and stirred for 3 hours. After completion of the reaction, the mixture was concentrated by rotatory evaporation. The residue was dried under high vacuum for 3 hours to provide crude primary amine TFA salt as a yellow oil, which was used directly in the next step without further purification.

ii. To a 10 mL reaction tube was added acid **32** (12.2 mg, 0.054 mmol, 1.0 equiv), HATU (20.6 mg, 0.054 mmol, 1.0 equiv), DIPEA (19 µL, 0.11 mmol, 2.0 equiv) and DCM (0.5 mL). After stirring at room temperature for 4 h, the reaction was quenched with saturated *aq.* NH_4Cl and extracted with DCM (3 × 1 mL). The combined organic layer was dried over Na_2SO_4 and concentrated *in vacuo*. The resulting activated ester was then transferred into another 10 mL

reaction tube containing the crude primary amine TFA salt prepared above. DCM (0.5 mL) and DIPEA (28 μL, 0.16 mmol, 3.0 equiv) was added and the reaction mixture was stirred at room temperature for 12 hours before quenching with saturated aq. NH_4Cl. The layers were separated, and the aqueous phase was extracted with DCM (3×1 mL). The combined organic layer was dried over Na_2SO_4 and concentrated *in vacuo*. The residue was purified by preparative TLC (DCM/acetone = 4:1) to afford gymnastatin G (**11**) (9.1 mg, 37% yield, 2 steps) and 1-*epi*-gymnastatin G (**33**) (5.5 mg, 22% yield, 2 steps), both as a colorless oil which slowly solidifies. Gymnastatin G (**11**): $[\alpha]_D^{20}$ –32.1 (*c* 0.48, acetone). ^{1}H NMR (600 MHz, Acetone) δ 7.25 (d, *J* = 8.1 Hz, 1H), 7.14 (d, *J* = 15.4 Hz, 1H), 6.04 (d, *J* = 15.4 Hz, 1H), 5.67 (d, *J* = 4.8 Hz, 1H), 5.62 (d, *J* = 9.8 Hz, 1H), 5.18 (s, 1H), 4.18 – 4.08 (m, 2H), 3.87 (d, *J* = 3.9 Hz, 1H), 3.85 – 3.79 (m, 2H), 3.67 (d, *J* = 10.8 Hz, 1H), 2.62 – 2.51 (m, 1H), 2.16 (dd, *J* = 13.0, 5.4 Hz, 1H), 2.06 (t, *J* = 13.0 Hz, 1H), 1.76 (s, 3H), 1.42 – 1.35 (m, 1H), 1.34 – 1.22 (m, 9H), 0.97 (d, *J* = 6.6 Hz, 3H), 0.86 (t, *J* = 6.8 Hz, 3H). ^{13}C NMR (151 MHz, Acetone) δ 199.1, 165.9, 146.9, 146.0, 132.2, 119.8, 80.9, 76.5, 75.6, 70.7, 61.2, 56.0, 47.2, 38.1, 36.5, 33.8, 32.6, 30.1, 28.2, 23.3, 20.9, 14.3, 12.7. IR (thin film) ν_{max} (cm^{-1}) 3318, 2957, 2925, 2853, 1732, 1651, 1610, 1542, 1462, 1276, 1262, 1096, 844, 764, 751. HRMS (ESI) *calcd.* for $[C_{23}H_{34}O_6NClNa]^+$ ($[M+Na]^+$): *m/z* 478.1967, found: 478.1960.

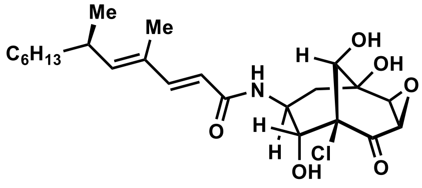

1-*epi*-gymnastatin G (**33**)

1-*epi*-gymnastatin G (**33**): $[\alpha]_D^{20}$ –50.0 (*c* 0.55, acetone). ^{1}H NMR (600 MHz, Acetone) δ 7.38 (d, *J* = 7.3 Hz, 1H), 7.17 (dd, *J* = 15.4, 0.8 Hz, 1H), 5.94 (d, *J* = 15.4 Hz, 1H), 5.65 (d, *J* = 9.6 Hz, 1H), 5.24 (d, *J* = 4.3 Hz, 1H), 5.11 (s, 1H), 4.16 (dddd, *J* = 12.8, 10.5, 7.3, 5.5 Hz, 1H), 3.98 (dd, *J* = 10.5, 4.4 Hz, 1H), 3.94 (d, *J* = 8.8 Hz, 1H), 3.84 (dd, *J* = 8.9, 1.5 Hz, 1H), 3.74 – 3.72 (m, 2H), 2.57 (m, 1H), 2.45 (dd, *J* = 13.3, 5.5 Hz, 1H), 1.93 (t, *J* = 13.0 Hz, 1H), 1.77 (d, *J* = 1.3 Hz, 3H), 1.42 – 1.36 (m, 1H), 1.33 – 1.22 (m, 9H), 0.98 (d, *J* = 6.6 Hz, 3H), 0.87 (t, *J* = 7.0 Hz, 3H). ^{13}C NMR (151 MHz, Acetone) δ 194.0, 167.6, 147.4, 146.4, 132.2, 119.5, 83.2, 80.7, 79.4, 70.7, 61.0, 54.5, 51.2, 38.6, 38.0, 33.8, 32.6, 30.1, 28.2, 23.3, 20.9, 14.3, 12.7. IR (thin film) ν_{max} (cm^{-1}) 3342, 2956, 2924, 2853, 1733, 1651, 1611, 1544, 1459, 1377, 1293, 1080, 980, 845. HRMS (ESI) *calcd.* for $[C_{23}H_{34}O_6NClNa]^+$ ($[M+Na]^+$): *m/z* 478.1967, found: 478.1960.

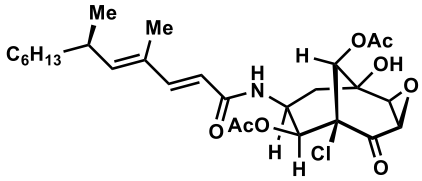

gymnastatin G diacetate (**SI-5**)

Gymnastatin G diacetate (**SI-5**): To a 10 mL reaction tube was added subsequently gymnastatin G (2.4 mg, 0.0053 mmol, 1.0 equiv), DMAP (trace, ~ 0.1 mg), DCM (0.3 mL), Et_3N (11 μL, 0.079 mmol, 15 equiv) and Ac_2O (2.5 μL, 0.026 mmol, 5 equiv). The reaction was stirred for 6 h before being quenched with saturated aq. NH_4Cl. The layers were separated, and the aqueous phase was extracted with DCM (3×1 mL). The combined organic layer was dried over Na_2SO_4 and concentrated *in vacuo*. The residue was purified by preparative TLC (hexanes/acetone = 2:1) to afford gymnastatin G diacetate (**1.67**) (1.0 mg, 35% yield) as a colorless oil. $[\alpha]_D^{20}$ –31.0 (*c* 0.10, $CHCl_3$). ^{1}H NMR (600 MHz, $CDCl_3$) δ 7.20 (d, *J* = 15.2 Hz, 1H), 5.66 (d, *J* = 9.9 Hz, 1H), 5.64 (d, *J* = 2.1 Hz, 1H), 5.61 (d, *J* = 15.2 Hz, 1H), 5.40 (d, *J* = 8.1 Hz, 1H), 5.33 (dd, *J* = 3.2, 1.2 Hz, 1H), 4.45 – 4.37 (m, 1H), 3.78 (d, *J* = 3.6 Hz, 1H), 3.59 (dd, *J* = 3.7, 2.0 Hz, 1H), 3.02 (s, 1H), 2.54 – 2.47 (m, 1H), 2.44 (ddd, *J* = 13.2, 5.1, 1.3 Hz, 1H), 2.26 (s, 3H), 2.18 (s, 3H), 2.05 (t, *J* = 13.1 Hz, 1H), 1.74 (d, *J* = 1.3 Hz, 3H), 1.38 – 1.31

(m, 1H), 1.31 – 1.18 (m, 9H), 0.97 (d, *J* = 6.6 Hz, 3H), 0.87 (t, *J* = 7.0 Hz, 3H). ^{13}C NMR (151 MHz, $CDCl_3$) δ 194.6, 171.7, 169.6, 166.0, 149.1, 148.4, 130.9, 116.2, 74.5, 74.1, 73.8, 71.1, 58.2, 53.9, 45.5, 37.4, 37.2, 33.4, 32.0, 29.5, 27.6, 22.8, 20.99, 20.97, 20.6, 14.2, 12.7. IR (thin film) v_{max} (cm^{-1}) 3361, 2957, 2924, 2852, 1737, 1653, 1614, 1533, 1459, 1375, 1218, 1087, 1060, 1017, 896, 799. HRMS (ESI) *calcd.* for $[C_{27}H_{38}O_8NClNa]^+$ ($[M+Na]^+$): *m/z* 562.2178, found: 562.2184.

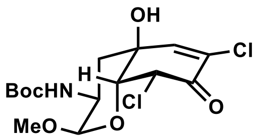

37

Acetal **37**: To a 10 mL reaction tube was added compound **18** (1.6 mg, 0.0046 mmol, 1.0 equiv), THF (0.3 mL) and MeOH (1.8 µL, 0.046 mmol, 10 equiv). The resulting solution was cooled to –78 °C and KHMDS (0.5 M in toluene, 9.1 µL, 0.0046 mmol, 1.0 equiv) was added. The reaction was stirred at –78 °C for 3 h before being quenched with saturated *aq.* NH_4Cl. The layers were separated, and the aqueous phase was extracted with Et_2O (3×1 mL). The combined organic layer was dried over Na_2SO_4 and concentrated *in vacuo*. The yield was determined by ^{1}H-NMR of the crude mixture with 1,3,5-trimethoxybenzene as the internal standard. The crude mixture was purified by preparative TLC (hexanes/EtOAc = 2:1) to afford pure compound **37** as a colorless oil (50%). ^{1}H NMR (600 MHz, $CDCl_3$) δ 6.79 (s, 1H), 5.25 (d, *J* = 2.3 Hz, 1H), 4.77 (d, *J* = 9.4 Hz, 1H), 4.64 (d, *J* = 3.5 Hz, 1H), 4.22 (t, *J* = 2.5 Hz, 1H), 3.80 – 3.70 (m, 1H), 3.46 (s, 3H), 2.24 (s, 1H), 2.21 (dd, *J* = 12.3, 4.5 Hz, 1H), 1.96 (t, *J* = 12.4 Hz, 1H), 1.44 (s, 9H). ^{13}C NMR (151 MHz, $CDCl_3$) δ 183.0, 155.1, 141.7, 134.2, 97.2, 80.5, 75.4, 69.8, 60.7, 55.4, 47.6, 39.4, 28.5. IR (thin film) v_{max} (cm^{-1}) 3362, 2958, 2923, 2850, 1723, 1689, 1611, 1511, 1454, 1392, 1369, 1290, 1253, 1165, 1098, 1040, 1002, 749. HRMS (ESI) *calcd.* for $[C_{15}H_{21}O_6NCl_3]^-$ ($[M+Cl]^-$): *m/z* 416.0440, found: 416.0447.

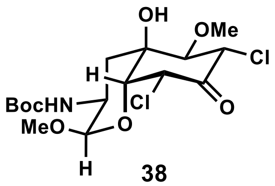

38

Acetal **38**: To a 10 mL reaction tube was added lactol **18** (3.8 mg, 0.011 mmol, 1.0 equiv) and MeOH (0.3 mL). The resulting solution was cooled to –20°C and NaOMe (0.7 mg, 0.013 mmol, 1.2 equiv, dissolved in MeOH) was added. The reaction mixture was stirred at –20°C for 2 hours before being quenched with saturated *aq.* NH_4Cl. The layers were separated, and the aqueous phase was extracted with DCM (3×1 mL). The combined organic layer was dried over Na_2SO_4 and concentrated *in vacuo*. The residue was purified by preparative TLC (Et_2O/hexanes = 3:2) to afford acetal **38** (1.9 mg, 42% yield) as a colorless oil, together with **37** (0.9 mg, 20% yield). $[\alpha]_D^{20}$ –43.2 (*c* 0.19, $CHCl_3$). ^{1}H NMR (600 MHz, $CDCl_3$) δ 5.09 (dd, *J* = 3.6, 1.1 Hz, 1H), 4.79 (d, *J* = 5.2 Hz, 1H), 4.78 (d, *J* = 9.6 Hz, H), 4.67 (d, *J* = 3.9 Hz, 1H), 4.27 (d, *J* = 3.6 Hz, 1H), 3.92 (ddt, *J* = 13.0, 8.9, 4.7 Hz, 1H), 3.83 (s, 3H), 3.69 (d, *J* = 10.0 Hz, 1H), 3.45 (s, 3H), 2.79 (s, 1H), 2.35 (dd, *J* = 12.6, 5.2 Hz, 1H), 1.75 (t, *J* = 12.6 Hz, 1H), 1.44 (s, 9H). ^{13}C NMR (151 MHz, $CDCl_3$) δ 190.2, 155.0, 97.8, 83.7, 80.2, 73.7, 72.7, 66.3, 63.4, 61.1, 55.5, 47.7, 35.6, 28.5. IR (thin film) v_{max} (cm^{-1}) 2957, 2921, 2851, 1721, 1695, 1461, 1377, 1261, 1050, 800, 764, 751. HRMS (ESI) *calcd.* for $[C_{16}H_{25}O_7NCl_2Na]^+$ ($[M+Na]^+$): *m/z* 436.0900, found: 436.0905.

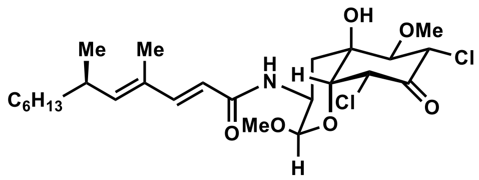

dankastatin C (**14**)

Dankastatin C (**14**): *i*. To a 10 mL reaction tube was added acetal **38** (3.0 mg, 0.0073 mmol, 1.0 equiv) and DCM (0.3 mL). Trifluoroacetic acid (0.06 mL) was added and the reaction mixture was stirred for 1 hour. After completion of the reaction, the mixture was concentrated by rotatory evaporation. The residue was dried under high vacuum for 3 hours to provide crude primary amine TFA salt as a yellow oil, which was used directly in the next step without further purification.
ii. To a 10 mL reaction tube was added acid **32** (2.0 mg, 0.0087 mmol, 1.2 equiv), HATU (3.6 mg, 0.0094 mmol, 1.3 equiv), DIPEA (6.3 μL, 0.036 mmol, 5.0 equiv) and DCM (0.3 mL). After stirring at room temperature for 4 h, the reaction was quenched with saturated *aq.* NH_4Cl and extracted with DCM (3 × 1 mL). The combined organic layer was dried over Na_2SO_4 and concentrated *in vacuo*. The resulting activated ester was then transferred into another 10 mL reaction tube containing the crude primary amine TFA salt prepared above. DMAP (~0.1 mg, catalytic amount), DCM (0.3 mL) and DIPEA (6.3 μL, 0.036 mmol, 5.0 equiv) was added and the reaction mixture was stirred at room temperature for 1 h before quenching with saturated aq. NH_4Cl. (Note: longer reaction time will result in the E1cb elimination of the methoxy group). The layers were separated, and the aqueous phase was extracted with DCM (3×1 mL). The combined organic layer was dried over Na_2SO_4 and concentrated *in vacuo*. The residue was purified by preparative TLC (hexane/EtOAc = 2:1) to afford dankastatin C (**14**) as a colorless oil (2.3 mg, 61% yield over 2 steps, yield includes the diastereomer formed from *ent*-**38**). $[\alpha]_D^{20}$ –72.9 (*c* 0.07, $CHCl_3$). ^1H NMR (600 MHz, $CDCl_3$) δ 7.25 (d, 1H), 5.72 (d, *J* = 15.6 Hz, 1H), 5.71 (d, *J* = 8.4 Hz, 1H), 5.65 (d, *J* = 9.8 Hz, 1H), 5.10 (d, *J* = 3.0 Hz, 1H), 4.79 (dd, *J* = 9.9, 0.9 Hz, 1H), 4.69 (d, *J* = 3.9 Hz, 1H), 4.41 – 4.34 (m, 1H), 4.30 (d, *J* = 3.6 Hz, 1H), 3.87 (s, 3H), 3.76 (d, *J* = 10.0 Hz, 1H), 3.48 (s, 3H), 2.81 (s, 1H), 2.55 – 2.46 (m, 1H), 2.40 (dd, *J* = 12.7, 5.1 Hz, 1H), 1.77 (d, *J* = 1.2 Hz, 3H), 1.76 (t, *J* = 12.6 Hz, 1H), 1.40 – 1.12 (m, 10H), 0.97 (d, *J* = 6.7 Hz, 3H), 0.87 (t, *J* = 7.1 Hz, 3H). ^{13}C NMR (151 MHz, $CDCl_3$) δ 190.1, 166.3, 148.6, 147.5, 130.9, 117.0, 97.7, 83.6, 73.8, 72.6, 66.3, 63.4, 61.1, 55.5, 46.3, 37.4, 35.2, 33.4, 32.0, 29.5, 27.6, 22.8, 20.7, 14.2, 12.7. IR (thin film) v_{max} (cm^{-1}) 2956, 2923, 2852, 1725, 1648, 1612, 1530, 1462, 1378, 1033, 982, 846. HRMS (ESI) *calcd.* for $[C_{25}H_{39}O_6NCl_2Na]^+$ ($[M+Na]^+$): *m/z* 542.2048, found: 542.2050.

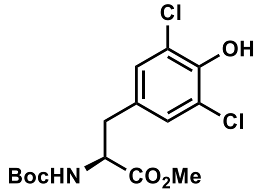

SI-6

3'5'-Dichloro-*N*-Boc-L-tyrosine methyl ester (**SI-6**): To a 25 mL round-bottom flask was added *L*-Boc-Tyr-OMe (**24**) (500 mg, 1.69 mmol, 1.0 equiv) and acetic acid (13 mL). A stream of argon was gently bubbled through the solution and to the solution was added dropwise SO_2Cl_2 (0.41 mL, 5.1 mmol, 3.0 equiv). After stirring for 30 minutes, another portion of SO_2Cl_2 (0.41 mL, 5.1 mmol, 3.0 equiv) was added and the reaction was continued for another 30 minutes. The reaction was then poured into a mixture of Et_2O (50 mL) and saturated *aq.* $NaHCO_3$ (50 mL) with vigorous stirring. The layers were separated, and the aqueous phase was extracted with Et_2O (3×30 mL). The combined organic layer was concentrated *in vacuo*, redissolved in DCM (50 mL) and washed with saturated *aq.* $NaHCO_3$ (50 mL). The organic layer was then dried over Na_2SO_4 and concentrated *in vacuo*. The residue was purified by column chromatography (hexanes/EtOAc = 4:1) to afford ester **SI-6** (312 mg, 51% yield) as a yellow oil. ^1H NMR (600 MHz, $CDCl_3$) δ 7.03

(s, 2H), 5.79 (brs, 1H), 5.03 (brs, 1H), 4.51 (m, 1H), 3.74 (s, 3H), 3.08 – 3.01 (m, 1H), 2.96 – 2.89 (m, 1H), 1.44 (s, 9H). The ^{1}H NMR matches that reported in literature.[2]

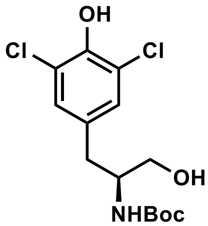

40

Alcohol **40**: To a 100 mL round-bottom flask was added ester **SI-6** (390 mg, 1.07 mmol, 1.0 equiv) and THF (16 mL). The reaction was cooled to –78 °C and DIBAL (1.0 M in hexanes, 6.4 mL, 6.4 mmol. 6.0 equiv) was added dropwise. After stirring at –78 °C for 30 min, the reaction mixture was moved to an ice-water bath and then quenched with saturated *aq.* potassium sodium tartrate (20 mL). The reaction mixture was stirred at room temperature for another 10 h. The layers were then separated, and the aqueous phase was extracted with Et_2O (3×30 mL). The combined organic layer was dried over Na_2SO_4 and concentrated *in vacuo*. The residue was purified by column chromatography (hexanes/EtOAc = 2:1) to afford alcohol **40** (237 mg, 66% yield) as a pale-yellow foam. $[\alpha]_D^{20}$ –53.0 (*c* 0.20, $CHCl_3$). ^{1}H NMR (600 MHz, $CDCl_3$) δ 7.13 (s, 2H), 5.87 (brs, 1H), 4.77 (d, *J* = 8.3 Hz, 1H), 3.77 (m, 1H), 3.66 (dd, *J* = 11.2, 3.8 Hz, 1H), 3.56 (dd, *J* = 11.0, 4.9 Hz, 1H), 2.75 (d, *J* = 7.3 Hz, 2H), 2.24 (brs, 1H), 1.42 (s, 9H). ^{13}C NMR (151 MHz, $CDCl_3$) δ 156.1, 146.6, 131.6, 129.1, 121.1, 80.1, 64.0, 53.7, 36.2, 28.5. IR (thin film) v_{max} (cm^{-1}) 3372, 2965, 2925, 2852, 1688, 1488, 1413, 1368, 1168, 1019, 797. HRMS (ESI) *calcd.* for $[C_{14}H_{19}O_4NCl_2Na]^+$ ($[M+Na]^+$): *m/z* 358.0583, found: 358.0585.

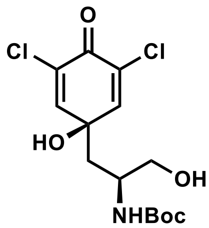

41

Diol **41**: To a 30 mL reaction tube was added alcohol **40** (28.5 mg, 0.0848, 1.0 equiv), tris[3,5-bis(trifluoromethyl)phenyl]phosphine (114 mg, 0.170 mmol, 2.0 equiv) and Cs_2CO_3 (30.4 mg, 0.0932, 1.1 equiv). The reaction tube was evacuated and then backfilled with O_2 using a balloon. Acetone (1.4 mL) was added, and the reaction tube was placed in a dry ice-acetone bath. The solvent level inside the reaction tube was kept ~1 cm below the cooling bath level. A solution of TPP (0.0020M in $CHCl_3$, 1.4 mL) was added at –78 °C, and the resulting mixture was irradiated with a fluorescent lamp for 24 h (the time required for the completion of the reaction varies depending on the scale of the reaction and TLC should be used to monitor the progress of the reaction). Upon completion of the reaction, saturated *aq*. NH_4Cl (2 mL) was added at –78 °C and the resulting mixture was allowed to warm to room temperature. The layers were separated, and the aqueous phase was extracted with DCM (3×5 mL). The combined organic layer was dried over Na_2SO_4 and concentrated *in vacuo*. The residue was purified by column chromatography (DCM/acetone = 4:1 to 3:1) to afford compound **41** (21.1 mg, 70% yield) as a colorless oil. $[\alpha]_D^{20}$ +29.0 (*c* 0.20, $CHCl_3$). ^{1}H NMR (600 MHz, $CDCl_3$) δ 7.12 (d, *J* = 2.8 Hz, 1H), 7.11 (d, *J* = 2.8 Hz, 1H), 5.07 (brs, 1H), 4.26 (s, 1H), 3.93 (brs, 1H), 3.75 – 3.62 (m, 2H), 2.06 (dd, *J* = 8.4, 3.6 Hz, 1H), 2.05 (brs, 1H), 1.98 (dd, *J* = 14.6, 8.7 Hz, 1H), 1.46 (s, 9H). ^{13}C NMR (151 MHz, $CDCl_3$) δ 172.7, 156.7, 147.3, 146.9, 131.05, 130.96, 81.3, 71.2, 65.9, 48.6, 43.9, 28.5. IR (thin film) v_{max} (cm^{-1}) 3368, 2954, 2925, 2852, 1685, 1520, 1457, 1394, 1368, 1250, 1166, 1108, 978, 748. HRMS (ESI) *calcd.* for $[C_{14}H_{19}O_5NCl_2Na]^+$ ($[M+Na]^+$): *m/z* 374.0532, found: 374.0533.

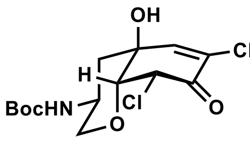
42

Oxa-decalin **42**: To a 10 mL reaction tube was added compound **41** (7.3 mg, 0.021 mmol, 1.0 equiv) and THF (0.6 mL). The solution was cooled to –78 °C and KHMDS (0.5 M in toluene, 8.3 μL, 0.0042 mmol, 0.2 equiv) was added. After that, the reaction mixture was warmed up slowly to –15 °C over a period of 40 min, before being quenched with saturated *aq*. NH_4Cl (1 mL). The layers were separated, and the aqueous phase was extracted with DCM (3×1 mL). The combined organic layer was dried over Na_2SO_4 and concentrated *in vacuo*. The residue was purified by column chromatography (DCM/acetone = 10:1) to afford compound **42** and *iso*-**42** (5.7 mg, 78% yield, dr = 10:1) as a colorless oil. **42** and *iso*-**42** can be separated by preparative TLC (7% MeOH/$CHCl_3$). Major diastereomer **42**: $[\alpha]_D^{20}$ –10.3 (*c* 0.36, $CHCl_3$). ^1H NMR (700 MHz, $CDCl_3$) δ 6.75 (brs, 1H), 5.26 (d, *J* = 2.3 Hz, 1H), 4.34 (brs, 1H), 4.11 (d, *J* = 10.0 Hz, 1H), 3.90 (t, *J* = 2.4 Hz, 1H), 3.70 (brs, 1H), 3.13 (t, *J* = 11.0 Hz, 1H), 2.49 (s, 1H), 2.45 (d, *J* = 12.0 Hz, 1H), 1.66 (t, *J* = 12.4 Hz, 1H), 1.43 (s, 9H). ^{13}C NMR (151 MHz, $CDCl_3$) δ 182.8, 155.0, 142.0, 134.1, 83.7, 80.6, 70.5, 70.0, 61.3, 45.0, 44.6, 28.4. IR (thin film) v_{max} (cm^{-1}) 3379, 2952, 2924, 2850, 1723, 1690, 1525, 1461, 1394. 1369, 1250, 1166, 1106, 1011, 748. HRMS (ESI) *calcd.* for $[C_{14}H_{19}O_5NCl_2Na]^+$ ($[M+Na]^+$): *m/z* 374.0532, found: 374.0533.

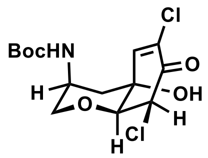
iso-**42**

Minor diastereomer *iso*-**42**: $[\alpha]_D^{20}$ +46.1 (*c* 0.23, $CHCl_3$). ^1H NMR (600 MHz, $CDCl_3$) δ 6.67 (d, *J* = 2.4 Hz, 1H), 5.29 (d, *J* = 2.4 Hz, 1H), 4.74 (d, *J* = 6.4 Hz, 1H), 3.99 (t, *J* = 2.4 Hz, 1H), 3.93 (dt, *J* = 12.3, 2.0 Hz, 1H), 3.87 – 3.82 (m, 1H), 3.70 (dd, *J* = 12.3, 2.1 Hz, 1H), 2.70 (d, *J* = 13.6 Hz, 1H), 2.21 (s, 1H), 1.87 (dd, *J* = 13.6, 3.4 Hz, 1H), 1.46 (s, 9H). ^{13}C NMR (151 MHz, $CDCl_3$) δ 182.8, 155.1, 143.6, 131.6, 84.6, 80.3, 71.1, 68.3, 61.2, 46.3, 41.8, 28.5. IR (thin film) v_{max} (cm^{-1}) 3371, 2957, 2920, 2852, 1678, 1458, 1397, 1369, 1251, 1168, 1041, 978, 747. HRMS (ESI) *calcd.* for $[C_{14}H_{19}O_5NCl_2Na]^+$ ($[M+Na]^+$): *m/z* 374.0532, found: 374.0534.

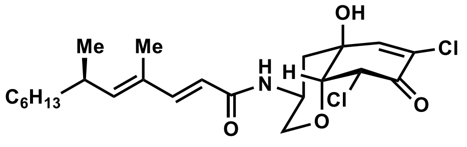
dankastatin B (**13**)

Dankastatin B (**13**): *i.* To a 10 mL reaction tube was added compound **42** (3.8 mg, 0.011 mmol, 1.0 equiv) and DCM (0.4 mL). Trifluoroacetic acid (0.08 mL) was added and the reaction mixture was stirred for 2 hours. After completion of the reaction, the mixture was concentrated by rotatory evaporation. The residue was dried under high vacuum for 3 hours to provide crude primary amine TFA salt as a yellow oil, which was used directly in the next step without further purification.

ii. To a 10 mL reaction tube was added acid **32** (2.4 mg, 0.011 mmol, 1.0 equiv), HATU (4.1 mg, 0.011 mmol, 1.0 equiv), DIPEA (5.7 μL, 0.032 mmol, 3.0 equiv) and DCM (0.5 mL). After stirring at room temperature for 4 h, the reaction was quenched with saturated *aq.* NH_4Cl and extracted with DCM (3 × 1 mL). The combined organic layer was dried over Na_2SO_4 and concentrated *in vacuo*. The resulting activated ester was then transferred into another 10 mL reaction tube containing the crude primary amine TFA salt prepared above. DCM (0.4 mL) and DIPEA (5.7 μL, 0.032 mmol, 3.0 equiv) was added and the reaction mixture was stirred at room temperature for 12 hours before quenching with saturated aq. NH_4Cl. The layers were separated, and the aqueous phase was extracted with DCM (3×1 mL). The combined organic layer was dried over Na_2SO_4 and concentrated *in vacuo*. The residue was purified by preparative TLC (DCM/acetone = 10:1)

to afford dankastatin B (**13**) (4.2 mg, 85% yield, 2 steps) as a colorless oil which slowly solidifies. $[\alpha]_D^{20}$ –36.3 (*c* 0.30, $CHCl_3$). ^{1}H NMR (700 MHz, $CDCl_3$) δ 7.18 (d, *J* = 15.3 Hz, 1H), 6.84 (d, *J* = 2.4 Hz, 1H), 5.73 (d, *J* = 15.4 Hz, 1H), 5.67 (d, *J* = 9.8 Hz, 1H), 5.59 (d, *J* = 8.1 Hz, 1H), 5.39 (d, *J* = 2.3 Hz, 1H), 4.36 (brs, 1H), 4.12 (ddd, *J* = 11.0, 4.8, 2.0 Hz, 1H), 4.07 – 4.00 (m, 1H), 3.96 (t, *J* = 2.3 Hz, 1H), 3.20 (t, *J* = 10.9 Hz, 1H), 2.53 – 2.47 (m, 1H), 2.44 (ddd, *J* = 12.3, 4.1, 2.0 Hz, 1H), 1.76 (t, *J* = 11.9 Hz, 1H), 1.74 (d, *J* = 1.2 Hz, 3H), 1.38 – 1.32 (m, 1H), 1.31 – 1.17 (m, 9H), 0.96 (d, *J* = 7.0 Hz, 3H), 0.86 (t, *J* = 7.1 Hz, 3H). ^{13}C NMR (151 MHz, $CDCl_3$) δ 184.1, 167.2, 149.2, 148.1, 143.6, 133.1, 130.9, 116.7, 84.0, 70.0, 69.7, 61.7, 44.1, 43.6, 37.3, 33.4, 32.0, 29.5, 27.6, 22.8, 20.6, 14.2, 12.6. IR (thin film) ν_{max} (cm^{-1}) 3359, 3194, 2956, 2922, 2852, 1722, 1658, 1633, 1539, 1467, 1423, 1107, 1055, 1031, 974, 806. HRMS (ESI) *calcd.* for $[C_{23}H_{32}O_4NCl_2]^-$ ($[M–H]^-$): *m/z* 456.1714, found: 456.1723.

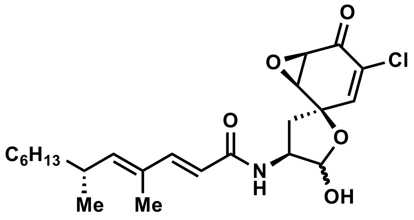

aranochloro A (**44**)

Aranochloro A (**44**) and Aranochloro B (**43**): To a 10 mL reaction tube was added aranorosin (5.0 mg, 0.012 mmol, 1.0 equiv), LiCl (0.61 mg, 0.014 mmol, 1.2 equiv, dissolved in THF) and THF (0.8 mL). The reaction was stirred at 22°C for 15 hours. The reaction was then quenched with saturated aq. NH_4Cl and extracted with DCM (3×1 mL). The combined organic layer was dried over Na_2SO_4 and concentrated *in vacuo*. ^{1}H NMR yields were obtained using 1,2,4,5-tetrachloro-3-nitrobenzene as internal standard. The crude mixture was then purified by preparative TLC (8% MeOH/DCM) to afford aranchloro A and B, gymnastatin G, and 1-*epi*-gymnastatin G. Aranochloro A and B was separated by a second preparative TLC (15% acetone/DCM). Aranochloro A (**44**): (only peaks from the major diastereomer at C-1 is listed) ^{1}H NMR (600 MHz, $CDCl_3$) δ 7.24 (under solvent peak), 6.67 (d, *J* = 2.7 Hz, 1H), 5.97 (d, *J* = 8.5 Hz, 1H), 5.75 (d, *J* = 15.3 Hz, 1H), 5.68 (d, *J* = 9.7 Hz, 1H), 5.56 (d, *J* = 4.5 Hz, 1H), 4.85–4.77 (m, 1H), 3.78 (dd, *J* = 3.9, 2.7 Hz, 1H), 3.63 (d, *J* = 3.7 Hz, 1H), 3.50 (brs, 1H), 2.55 (dd, *J* = 12.9, 8.5 Hz, 1H), 2.57 – 2.47 (m, 1H), 2.17 (ddd, *J* = 13.1, 11.0, 2.4 Hz, 1H), 1.77 (s, 3H), 1.40 – 1.20 (m, 10H), 0.97 (d, *J* = 6.6 Hz, 3H), 0.87 (t, *J* = 6.6 Hz, 3H). ^{13}C NMR (151 MHz, $CDCl_3$) δ 186.3, 166.7, 148.7, 147.7, 143.1, 130.9, 128.3, 117.0, 96.6, 80.7, 58.3, 53.0, 52.1, 39.2, 37.4, 33.4, 32.0, 29.5, 27.6, 22.8, 20.7, 14.2, 12.6. IR (thin film) ν_{max} (cm^{-1}) 3307, 2957, 2924, 2852, 1708, 1651, 1611, 1535, 1456, 1377, 1033, 984, 700. HRMS (ESI) *calcd.* for $[C_{23}H_{32}O_5NClNa]^+$ ($[M+Na]^+$): *m/z* 460.1861, found: 460.1863.

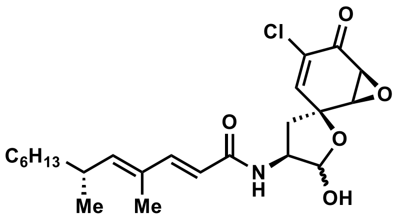

aranochloro B (**43**)

Aranochloro B (**43**): (only peaks from the major diastereomer at C1 is listed) ^{1}H NMR (600 MHz, $CDCl_3$) δ 7.24 (under solvent peak), 6.76 (d, *J* = 2.6 Hz, 1H), 5.93 (d, *J* = 8.4 Hz, 1H), 5.75 (d, *J* = 15.3 Hz, 1H), 5.68 (d, *J* = 9.7 Hz, 1H), 5.57 (d, *J* = 4.6 Hz, 1H), 4.89 – 4.82 (m, 1H), 3.67 (dd, *J* = 3.8, 2.6 Hz, 1H), 3.62 (d, *J* = 3.6 Hz, 1H), 3.37 (brs, 1H), 2.62 (dd, *J* = 13.1, 8.3 Hz, 1H), 2.54 – 2.47 (m, 1H), 2.13 (dd, *J* = 13.2, 10.8 Hz, 1H), 1.77 (s, 3H), 1.40 – 1.20 (m, 10H), 0.98 (d, *J* = 6.6 Hz, 3H), 0.87 (t, *J* = 6.9 Hz, 3H). ^{13}C NMR (151 MHz, $CDCl_3$) δ 186.2, 166.7, 148.7, 147.7, 144.5, 130.9, 128.0, 116.9, 96.7, 80.4, 57.0, 52.6, 51.7, 38.3, 37.4, 33.4, 32.0, 29.5, 27.6, 22.8, 20.7, 14.2, 12.7. IR (thin film) ν_{max} (cm^{-1}) 3358, 2956, 2923, 2851, 1707, 1651, 1611, 1531, 1461, 1377, 1022, 806, 699. HRMS (ESI) *calcd.* for $[C_{23}H_{32}O_5NClNa]^+$ ($[M+Na]^+$): *m/z* 460.1861, found: 460.1864.

Gymnastatin G diacetate spectra comparison

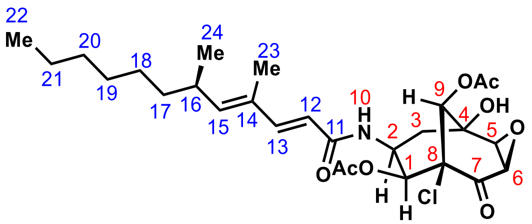

position	δ_H		δ_C	
	literature	this work	literature	this work
1	5.34 (dd, 3.1, 1.2)	5.33 (dd, 3.2, 1.2)	74.2 (CH)	74.5
2	4.40 (dddd, 13.1, 7.8, 5.0, 3.1)	4.45 – 4.37 (m)	45.3 (CH)	45.5
3	α 2.42 (ddd, 13.1, 5.0, 1.2)<br>β 2.09 (t,13.1)	α 2.44 (ddd, 13.2, 5.1, 1.3)<br>β 2.05 (t, 13.1)	36.9 (CH_2)	37.2
4			70.8 (C)	71.1
5	3.60 (dd, 3.7, 2.0)	3.59 (3.7, 2.0)	58.1 (CH)	58.2
6	3.79 (d, 3.7)	3.78 (d, 3.6)	53.7 (CH)	53.9
7			194.6 (C)	194.7
8			73.7 (C)	73.8
9	5.65 (d, 2.0)	5.64 (d, 2.1)	73.9 (CH)	74.1
10 (N*H*)	5.60 (d, 7.8)	5.40 (d, 8.1)		
11			166.0 (C)	166.0
12	5.62 (d, 15.1)	5.61 (d, 15.2)	116.0 (CH)	116.2
13	7.21 (d, 15.1)	7.20 (d, 15.2)	148.2 (CH)	148.4
14			130.7 (C)	130.9
15	5.66 (d, 9.8)	5.66 (d, 9.9)	149.0 (CH)	149.1
16	2.49 (m)	2.54 – 2.47 (m)	33.3 (CH)	33.4
17	a 1.33 (m)	b 1.38 – 1.31 (m)	37.2 (CH_2)	37.4
	b 1.25 (m)			
18	1.20 (m)		27.5 (CH_2)	27.6
19	1.22 (m)	1.31 – 1.18 (m, 9H)	29.4 (CH_2)	29.5
20	1.22 (m)		31.8 (CH_2)	32.0
21	1.26 (m)		22.6 (CH_2)	22.8
22	0.87 (t, 6.9)	0.87 (t, 7.0)	14.1 (CH_3)	14.2
23	1.73 (s)	1.74 (d, 1.3)	12.5 (CH_3)	12.7
24	0.96 (d, 6.6)	0.97 (d, 6.6)	20.5 (CH_3)	20.6
8-OH	3.48 (br. s)	3.02 (s)		
1-O*C*OCH_3			169.4 (C)	169.6
1-OCOC*H*$_3$	2.25 (s)	2.26 (s)	20.8 (CH_3)	20.97
9-O*C*OCH_3			171.5 (C)	171.7
9-OCOC*H*$_3$	2.18 (s)	2.18 (s)	20.9 (CH_3)	20.99

Dankastatin B spectra comparison

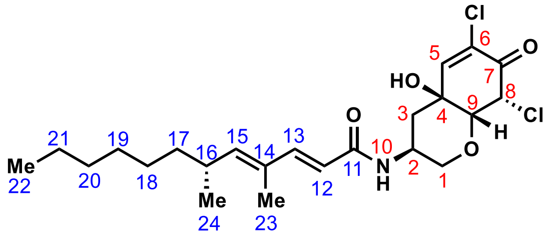

position	δ_H (*J*/Hz)		δ_C	
	literature	this work	literature	this work
1	α 4.13 (ddd, 10.8, 4.8, 2.1)<br>*β* 3.21 (t,10.8)	α 4.12 (ddd, 11.0, 4.8, 2.0)<br>*β* 3.20 (t,10.9)	69.9 (CH)	70.0
2	4.06 (ddddd, 12.1, 10.8, 8.0, 4.8, 4.1)	4.07 – 4.00 (m)	43.9 (CH)	44.1
3	α 2.47 (ddd, 12.1, 4.1, 2.1)<br>*β* 1.74 (t,12.1)	α 2.44 (ddd, 12.3, 4.1, 2.0)<br>*β* 1.76 (t, 11.9)	43.8 (CH_2)	43.6
4			69.6 (C)	69.7
5	6.83 (d, 2.5)	6.84 (d, 2.4)	142.7 (CH)	143.6
6		3.78 (d, 3.6)	133.4 (C)	133.1
7			183.2 (C)	184.1
8	5.35 (d, 2.3)	5.39 (d, 2.3)	61.4 (CH)	61.7
9	3.96 (dd, 2.5, 2.3)	3.96 (t, 2.3)	83.8 (CH)	84.0
10 (N*H*)	5.43 (br d, 8.0)	5.59 (d, 8.1)		
11			166.7 (C)	167.2
12	5.71 (d, 15.3)	5.73 (d, 15.4)	116.6 (CH)	116.7
13	7.20 (d, 15.3)	7.18 (d, 15.3)	147.8 (CH)	148.1
14			130.7 (C)	130.9
15	5.67 (dd, 9.6, 1.1)	5.67 (d, 9.8)	149.0 (CH)	149.2
16	2.50 (m)	2.53 – 2.47 (m)	33.3 (CH)	33.4
17	a 1.34 (m)	b 1.38 – 1.32 (m)	37.2 (CH_2)	37.3
	b 1.25 (m)			
18	1.21 (m)		27.5 (CH_2)	27.6
19	1.23 (m)	1.31 – 1.17 (m, 9H)	29.4 (CH_2)	29.5
20	1.23 (m)		31.8 (CH_2)	32.0
21	1.27 (m)		22.6 (CH_2)	22.8
22	0.87 (t, 7.1)	0.86 (t, 7.1)	14.1 (CH_3)	14.2
23	1.75 (d, 1.1)	1.74 (d, 1.2)	12.5 (CH_3)	12.6
24	0.97 (d, 6.6)	0.96 (d, 7.0)	20.5 (CH_3)	20.6
4-OH	3.85 (br s)	4.36 (br s)		

Dankastatin C spectra comparison

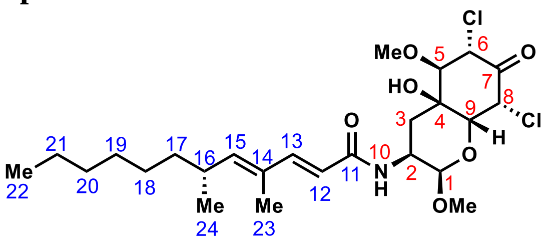

position	δ_H (*J*/Hz)		δ_C	
	literature	this work	literature	this work
1	4.69 (d, 3.9)	4.69 (d, 3.9)	97.5 (CH)	97.7
2	4.37 (dddd, 12.6, 8.9, 5.0, 3.9)	4.41 – 4.34 (m)	46.2 (CH)	46.3
3	α 2.40 (dd, 12.6, 5.0)<br>*β* 1.76 (t,12.6)	α 2.40 (dd, 12.7, 5.1)<br>*β* 1.76 (t, 12.6)	35.0 (CH_2)	35.2
4			72.4 (C)	72.6
5	3.76 (d, 10.1)	3.76 (d, 10.0)	83.5 (CH)	83.6
6	4.79 (dd, 10.1, 0.9)	4.79 (dd, 9.9, 0.9)	66.1 (CH)	66.3
7			190.0 (C)	190.1
8	5.11 (dd, 3.4, 0.9)	5.10 (d, 3.0)	61.0 (CH)	61.1
9	4.30 (d, 3.4)	4.30 (d, 3.6)	73.7 (CH)	73.8
10 (N*H*)	5.72 (br d, 8.9)	5.71 (d, 8.4)		
11			166.1 (C)	166.3
12	5.72 (d, 15.1)	5.72 (d, 15.6)	116.8 (CH)	117.0
13	7.25 (d, 15.1)	7.25 (d)	147.4 (CH)	147.5
14			130.8 (C)	130.9
15	5.65 (dd, 9.6, 1.1)	5.65 (d, 9.8)	148.4 (CH)	148.6
16	2.50 (m)	2.55 – 2.46 (m)	33.2 (CH)	33.4
17	a 1.35 (m)	1.40 – 1.12 (m, 10H)	37.3 (CH_2)	37.4
	b 1.23 (m)			
18	1.20 (m)		27.5 (CH_2)	27.6
19	1.24 (m)		29.4 (CH_2)	29.5
20	1.22 (m)		31.8 (CH_2)	32.0
21	1.27 (m)		22.6 (CH_2)	22.8
22	0.87 (t, 6.8)	0.86 (t, 7.1)	14.1 (CH_3)	14.2
23	1.76 (d, 1.1)	1.77 (d, 1.2)	12.5 (CH_3)	12.7
24	0.97 (d, 6.9)	0.96 (d, 7.0)	20.5 (CH_3)	20.7
1-OC*H*$_3$	3.48 (s)	3.48 (s)	55.3 (CH_3)	55.5
5-OC*H*$_3$	3.87 (s)	3.87 (s)	63.3 (CH_3)	63.4
4-OH	2.86 (br s)	2.81 (s)		

Aranochloro A spectra comparison

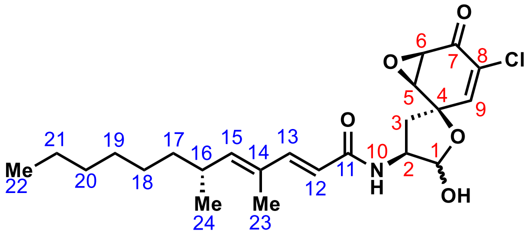

position	δ_H (*J*/Hz)		δ_C	
	literature	this work	literature	this work
1	5.54 (d, 4.4)	5.56 (d, 4.5)	96.48 (CH)	96.6
2	4.76 (m)	4.85 – 4.77 (m)	52.03 (CH)	52.1
3	α 2.57 (dd, 13, 8.5)<br>β 2.14 (dd, 13, 10.6)	α 2.55 (dd, 12.9, 8.5)<br>β 2.17 (ddd, 13.1, 11.0. 2.4)	39.09 (CH_2)	39.2
4			80.03 (C)	80.7
5	3.78 (dd, 3.6, 2.5)	3.78 (dd, 3.9, 2.7)	58.34 (CH)	58.3
6	3.60 (d, 3.6)	3.63 (d, 3.7)	53.03 (CH)	53.0
7			186.35 (C)	186.3
8			128.00 (C)	128.3
9	6.64 (d, 2.5)	6.67 (d, 2.7)	143.40 (CH)	143.1
10 (N*H*)	6.03 (d, 7.2)	5.97 (d, 8.5)		
11			167.07 (C)	166.7
12	5.74 (d, 15.2)	5.75 (d, 15.3)	116.82 (CH)	117.0
13	7.24 (d, 15.2)	7.24 (under sol.)	147.72 (CH)	147.7
14			130.83 (C)	130.9
15	5.65 (d, 10)	5.68 (d, 9.7)	148.89 (CH)	148.7
16	2.52 (m)	2.57 – 2.47 (m)	33.29 (CH)	33.4
17	1.28 (br s)	1.40 – 1.20 (m, 10H)	37.27 (CH_2)	37.4
18			27.52 (CH_2)	27.6
19			29.43 (CH_2)	29.5
20			31.88 (CH_2)	32.0
21			22.67 (CH_2)	22.8
22	0.91 (t, 6.5)	0.87 (t, 6.6)	14.03 (CH_3)	14.2
23	1.79 (d, 0.9)	1.77 (s)	12.55 (CH_3)	12.6
24	0.99 (d, 6.7)	0.97 (d, 6.6)	20.43 (CH_3)	20.7
1-OH	Not seen	3.50 (brs)		

Aranochloro B spectra comparison

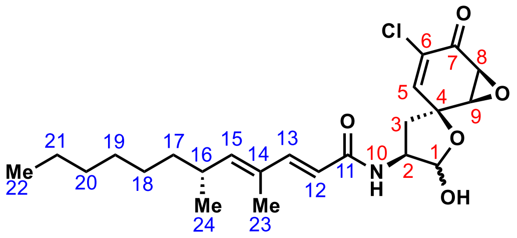

position	δ_H (*J*/Hz)		δ_C	
	literature	this work	literature	this work
1	5.55 (d, 4.3)	5.57 (d, 4.6)	96.45 (CH)	96.7
2	4.78 (m)	4.89 – 4.82 (m)	51.58 (CH)	51.7
3	α 2.64 (dd, 13, 10.6)<br>β 2.12 (dd, 13, 10.6)	α 2.66 (dd, 13.1, 8.3)<br>β 2.13 (dd, 13.2, 10.8)	38.07 (CH_2)	38.3
4			78.94 (C)	80.4
5	6.75 (d, 2.5)	6.76 (d, 2.6)	144.76 (CH)	144.5
6			127.43 (C)	128.0
7			186.21 (C)	186.2
8	3.62 (m)	3.62 (d, 3.6)	56.91 (CH)	57.0
9	3.62 (m)	3.67 (dd, 3.8, 2.6)	52.53 (CH)	52.6
10 (N*H*)	6.02 (d, 7.2)	5.93 (d, 8.4)		
11			166.99 (C)	166.7
12	5.74 (d, 15.2)	5.75 (d, 15.3)	116.70 (CH)	116.9
13	7.22 (d, 15.2)	7.24 (under solvent)	147.69 (CH)	147.7
14			130.4 (C)	130.9
15	5.65 (d, 10)	5.68 (d, 9.7)	148.83 (CH)	148.7
16	2.56 (m)	2.54 – 2.47 (m)	33.20 (CH)	33.4
17			37.15 (CH_2)	37.4
18			27.42 (CH_2)	27.6
19	1.28 (br s)	1.40 – 1.20 (m, 10H)	29.33 (CH_2)	29.5
20			31.76 (CH_2)	32.0
21			22.75 (CH_2)	22.8
22	0.91 (t, 6.8)	0.87 (t, 6.9)	14.03 (CH_3)	14.2
23	1.78 (d, 0.9)	1.77 (s)	12.45 (CH_3)	12.7
24	0.99 (d, 6.5)	0.98 (d, 6.6)	20.43 (CH_3)	20.7
1-OH	Not seen	3.37 (brs)		

Determination of optical purity using Mosher's ester/amide

General procedure: To the reaction vessel was added the alcohol or amine (1 equiv), DMAP (catalytical amount), Et_3N (6 equiv) and DCM. (*R*) or (*S*)-Mosher's acid chloride (3 equiv) was added at rt. The reaction mixture was stirred at rt or 40 °C for 12 h (for alcohol) or 1 h (for amine). The reaction was then quenched with saturated *aq*. NH_4Cl and extracted with ethyl acetate. The combined organic phases were dried over Na_2SO_4 and concentrate *in vacuo*. ^{1}H NMR was taken with the crude mixture. The NMR spectra of the crude Mosher's esters/amide derived from (*R*) and (*S*)-Mosher's acid were compared and the resonances with better separation were used for enantiomeric ratio determination. In some cases the crude mixture were purified by preparative TLC to help the assignment of the resonances in the crude NMR.

Gymnastatin G Route

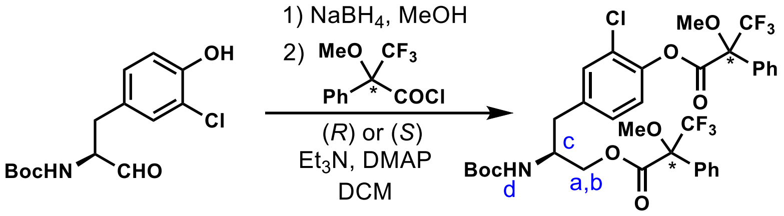

Green (top): (*R*)-Mosher's ester
Red (bottom): (*S*)-Mosher's ester

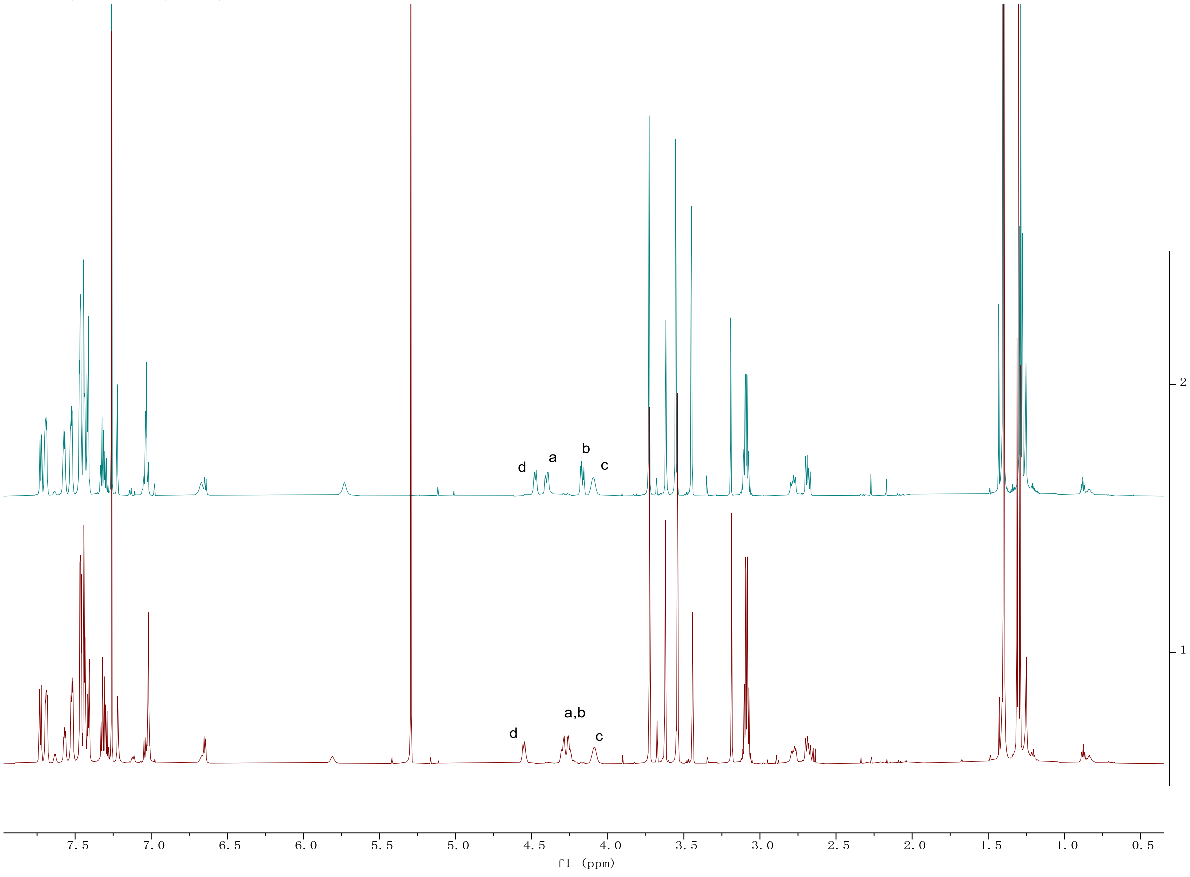

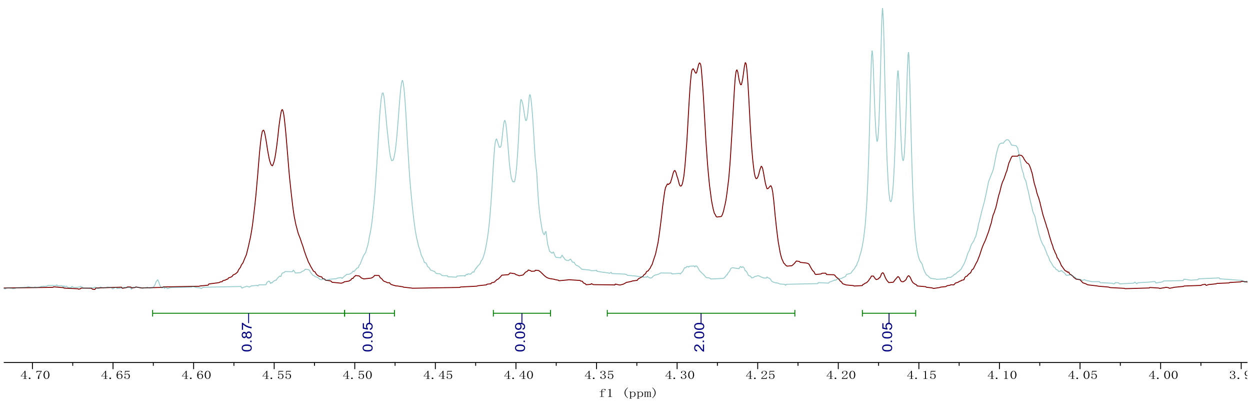

dr = 14:1, corresponding to 93% e.r.

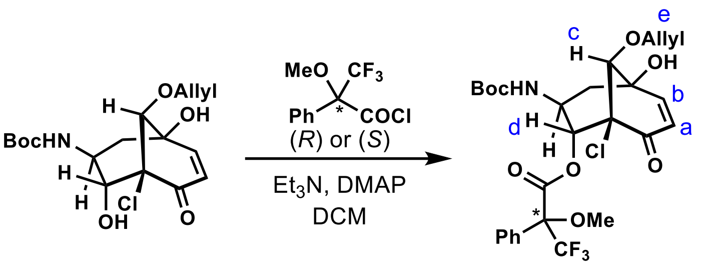

Green (top): (*R*)-Mosher's ester

Red (bottom): (*S*)-Mosher's ester

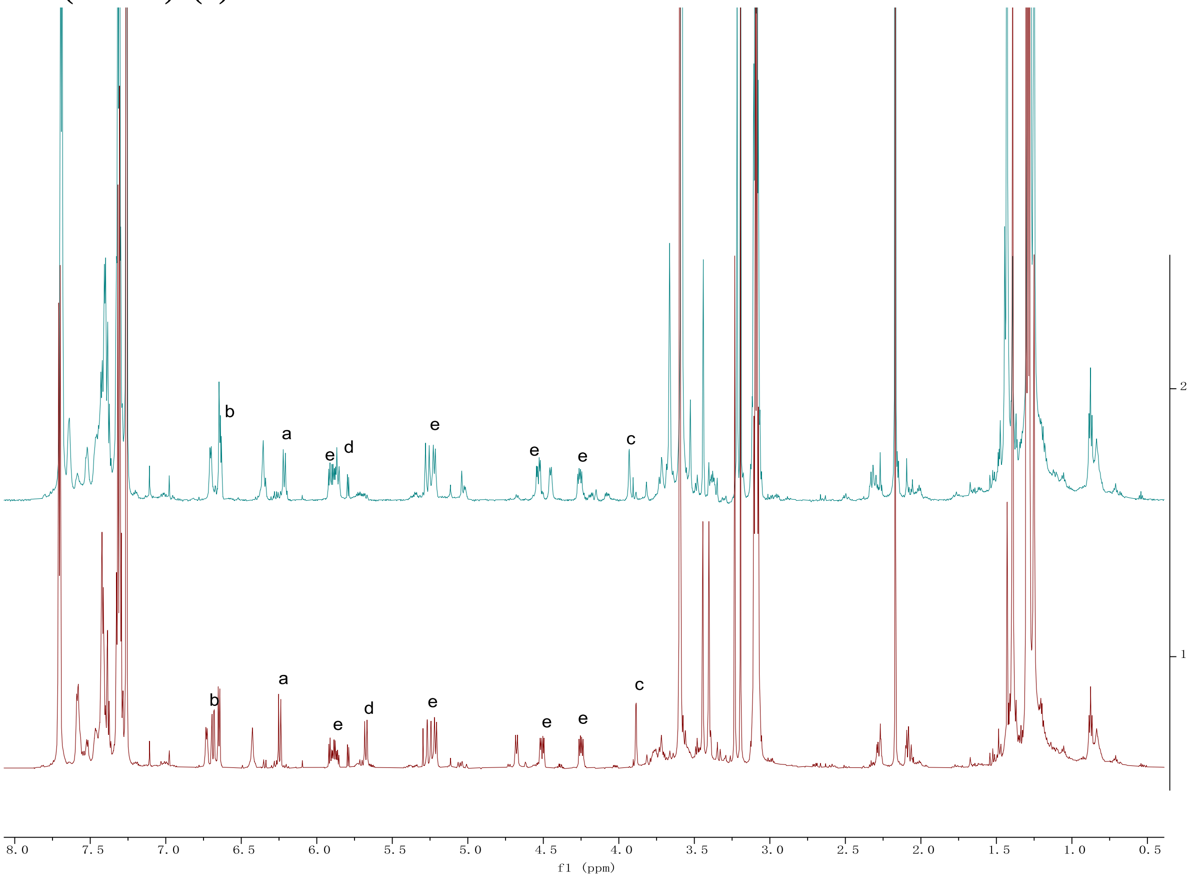

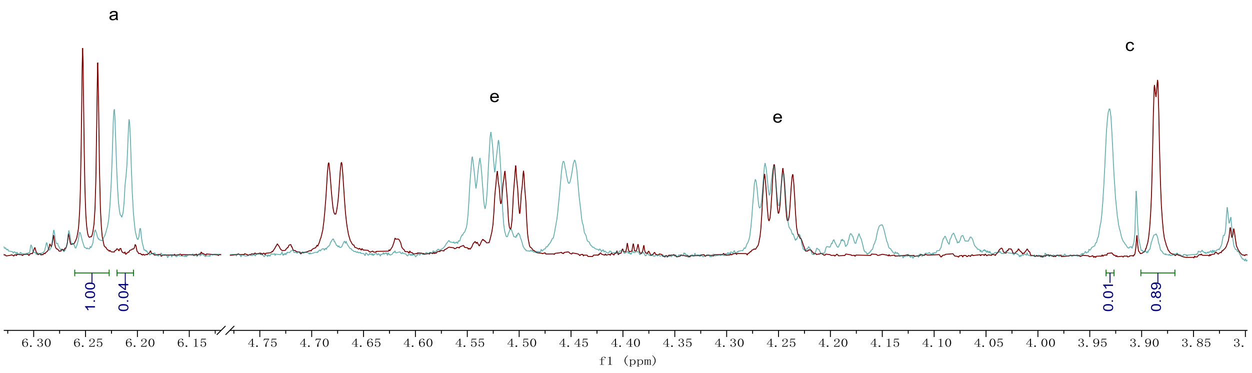

dr > 25:1, corresponding to > 96% e.r., as measure using (*R*)-Mosher's ester

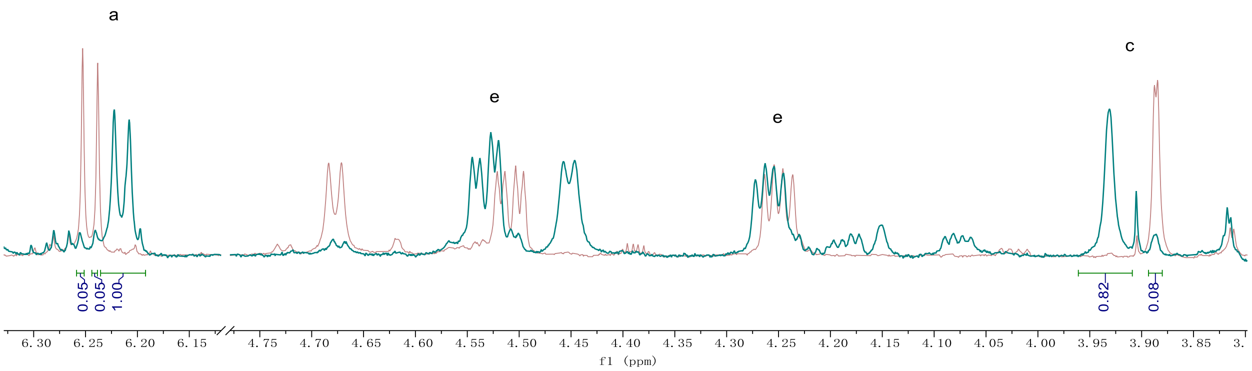

dr = 10:1, corresponding to 91% e.r., as measured by (*S*)-Mosher's ester

The difference might be due to kinetic resolution, thus the overall e.r. ≥ 91%

Dankastatin B Route

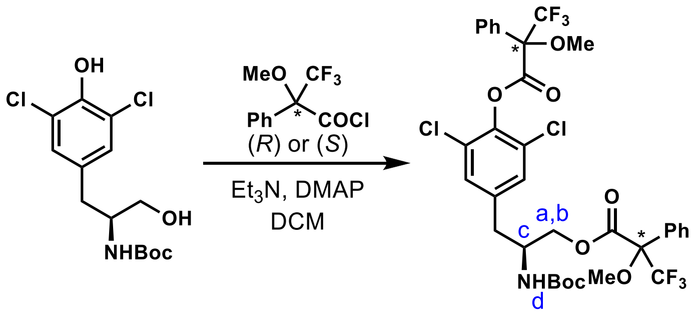

Green (top): (*R*)-Mosher's ester
Red (bottom): (*S*)-Mosher's ester

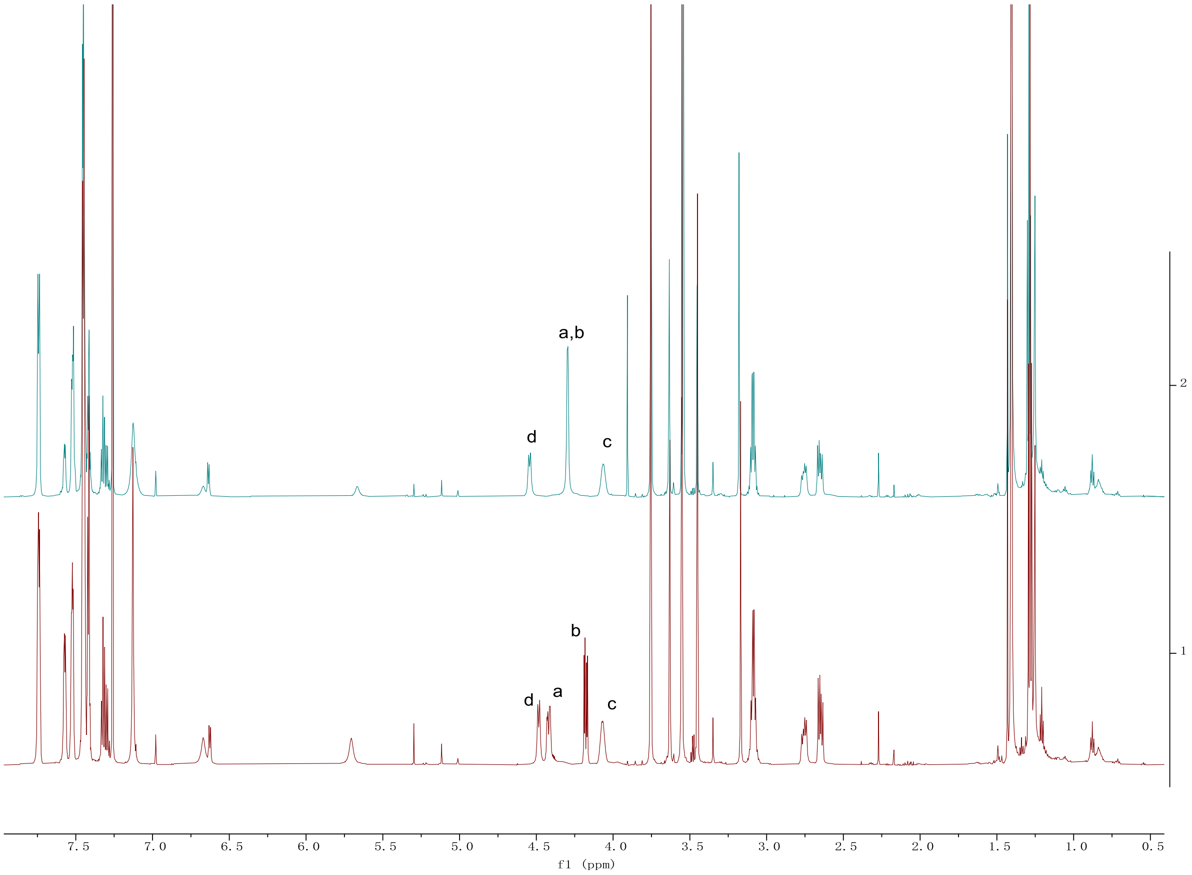

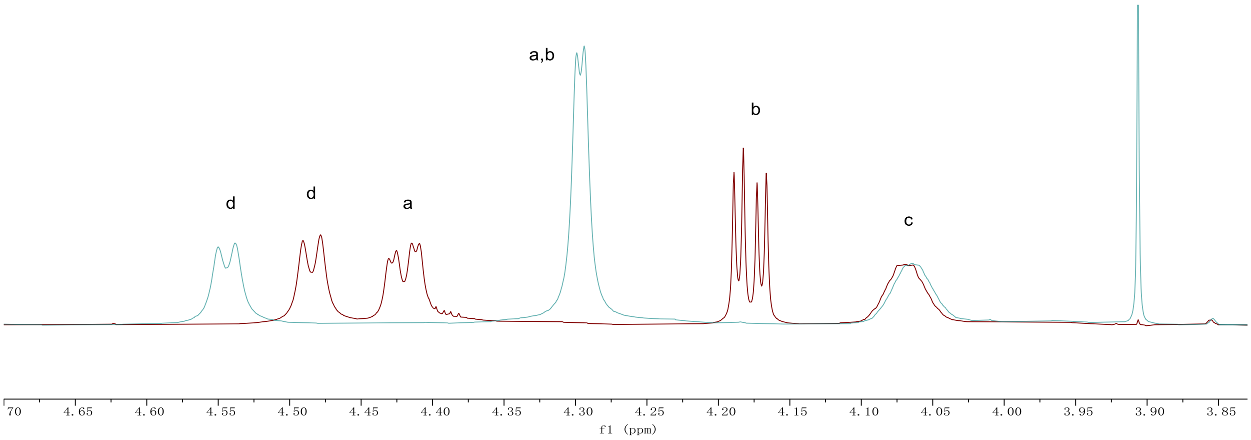

Single diastereomer (under NMR detecting limit), corresponding single enantiomer for **40**.

Dankastatin C Route

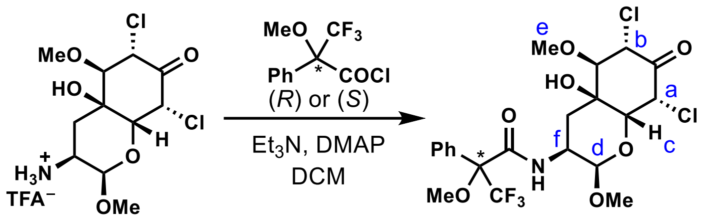

Green (top): (*S*)-Mosher’s amide
Red (bottom): (*R*)-Mosher’s amide

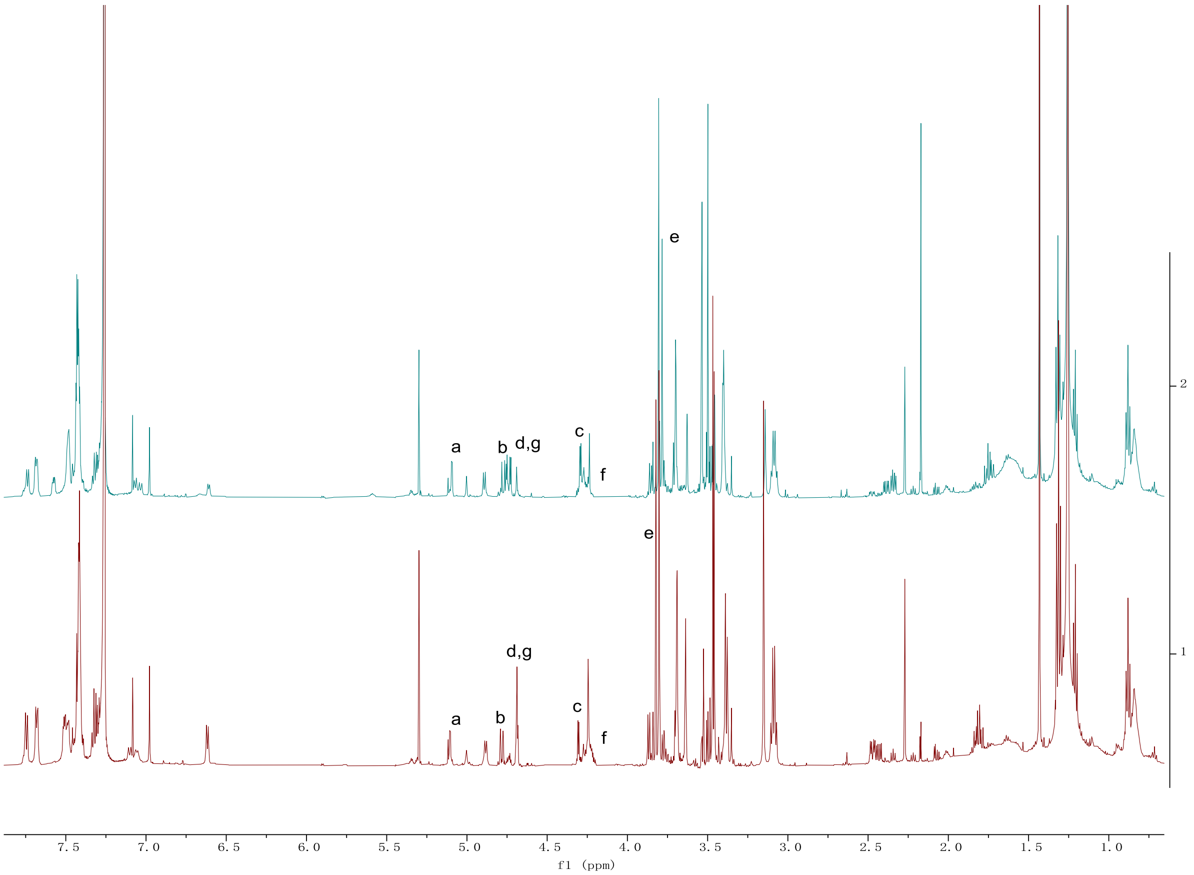

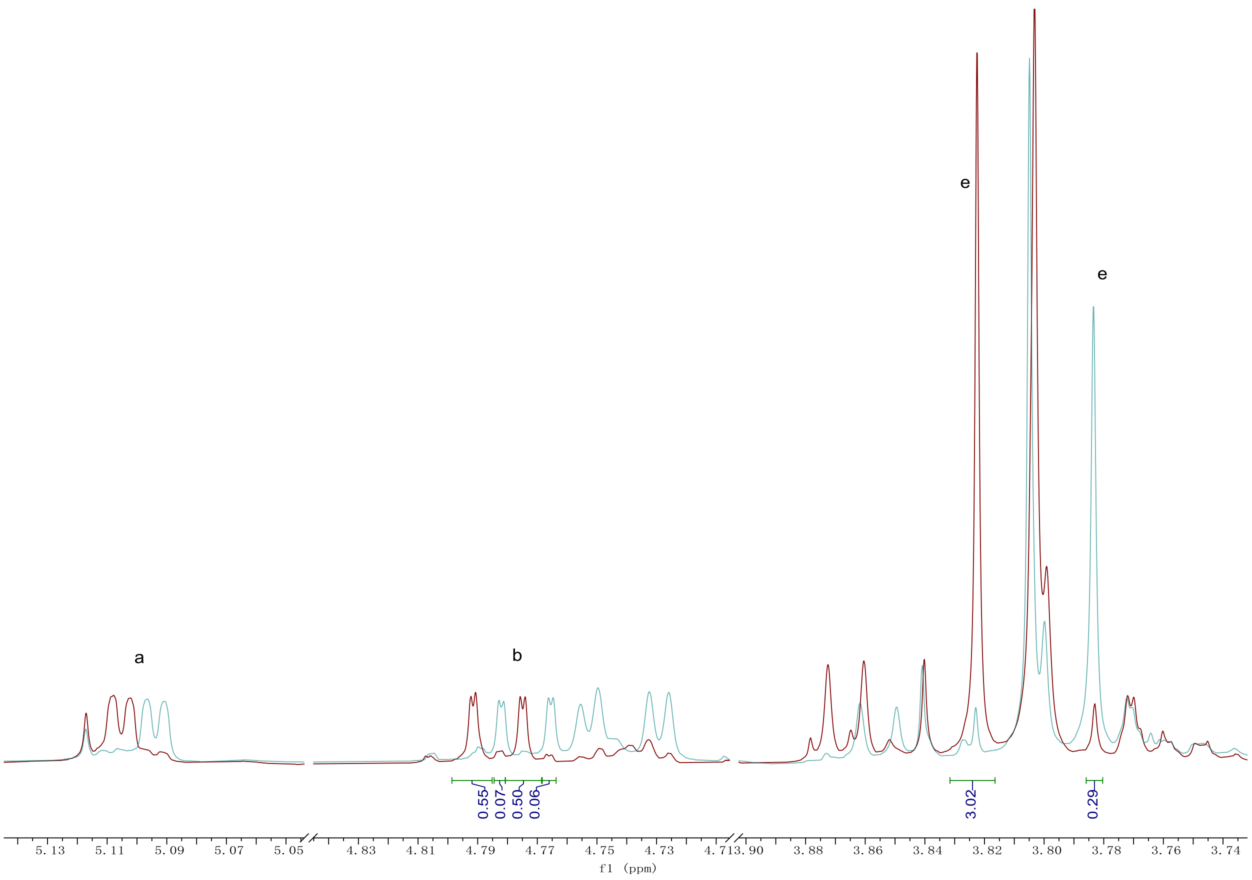

dr = 10:1, corresponding to 91% e.r.

Since the last step of the synthesis of dankastatin C was the amide coupling with enantio-pure acid **32**, the enantiomers of **38** were converted to a pair of diastereomers. Notably, the two diastereomers were separable on preparative TLC, thus dankastatin C (**14**) can be obtained in high optical purity regardless of the e.r. of compound **38**.

The ^{1}H NMR of dankastatin C and 6'-*epi*-dankastatin C only have subtle differences (see spectra below, expanded area).

Red: dankastatin C; green: 6’-*epi*-dankastatin C (major) + dankastatin C (minor)

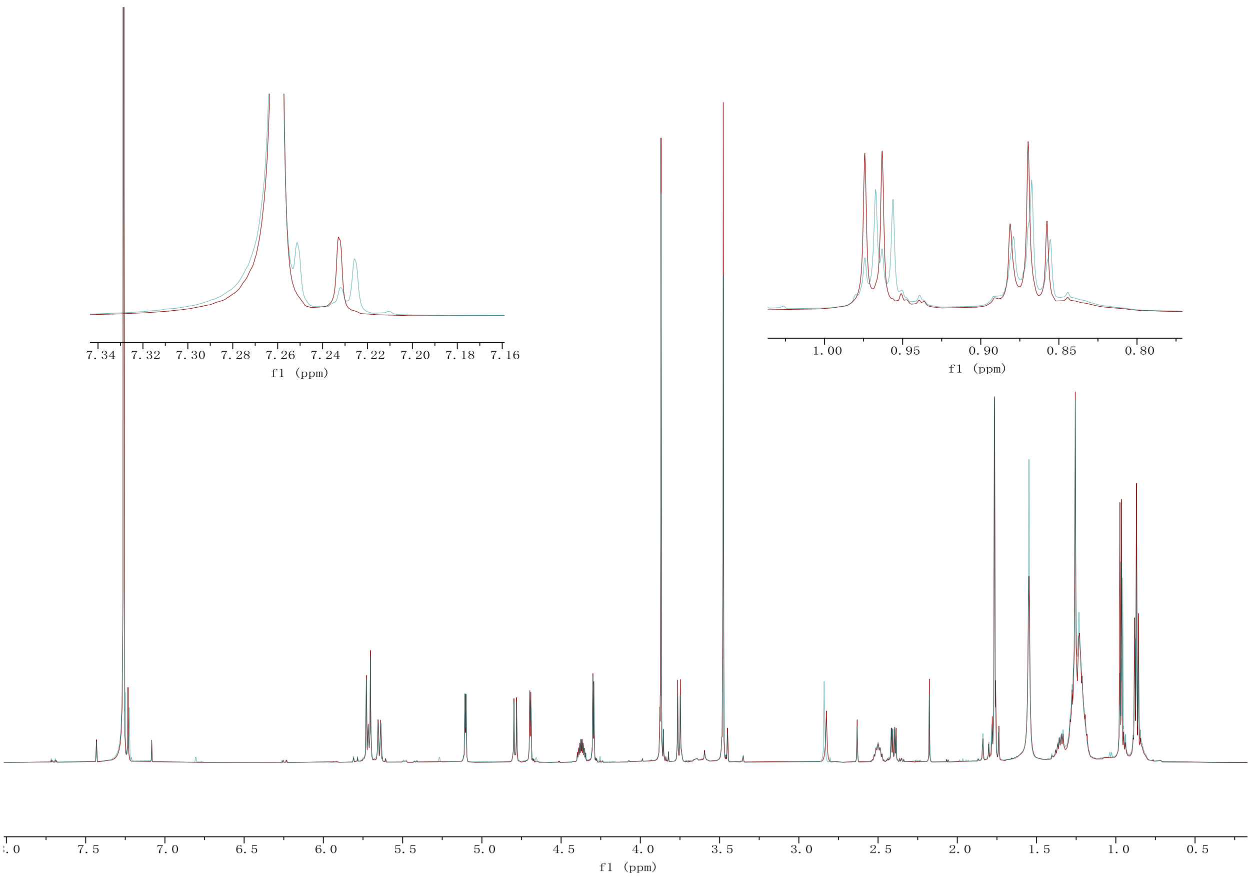

References

[1] Spradlin, J. N. et. al. *Nat. Chem. Biol*. **2019**, *15,* 747.

[2] Restrepo, M. P., Jaramillo, E. G., Martínez, A. M., Restrepo, S. R. *Med. Chem. Res*. **2018**, *27*, 2454.

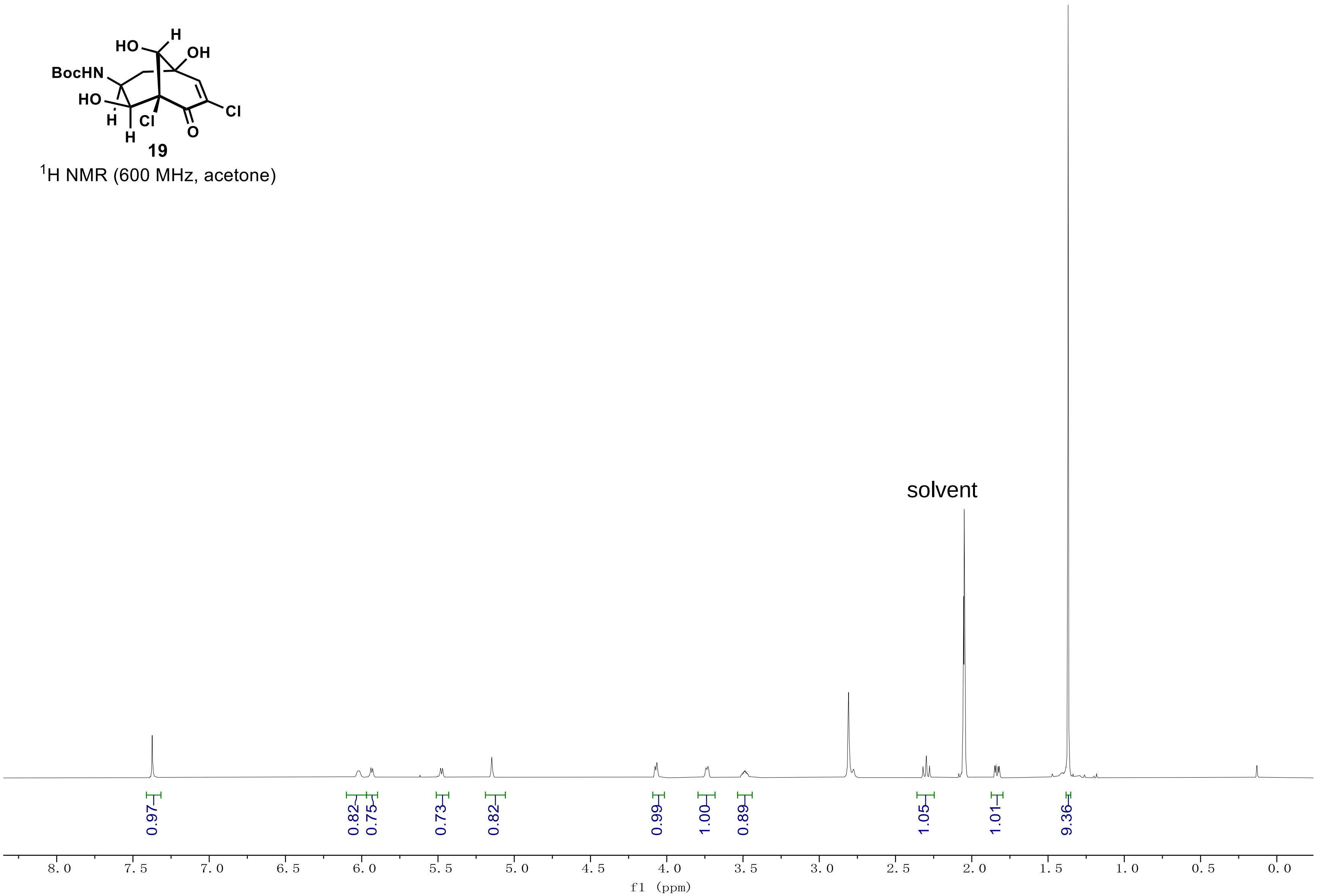
HO
H
OH
BocHN
HO
H
Cl
H
Cl
O
19
^{1}H NMR (600 MHz, acetone)
solvent
0.97
0.82
0.75
0.73
0.82
0.99
1.00
0.89
1.05
1.01
9.36
8.0 7.5 7.0 6.5 6.0 5.5 5.0 4.5 4.0 3.5 3.0 2.5 2.0 1.5 1.0 0.5 0.0
f1 (ppm)

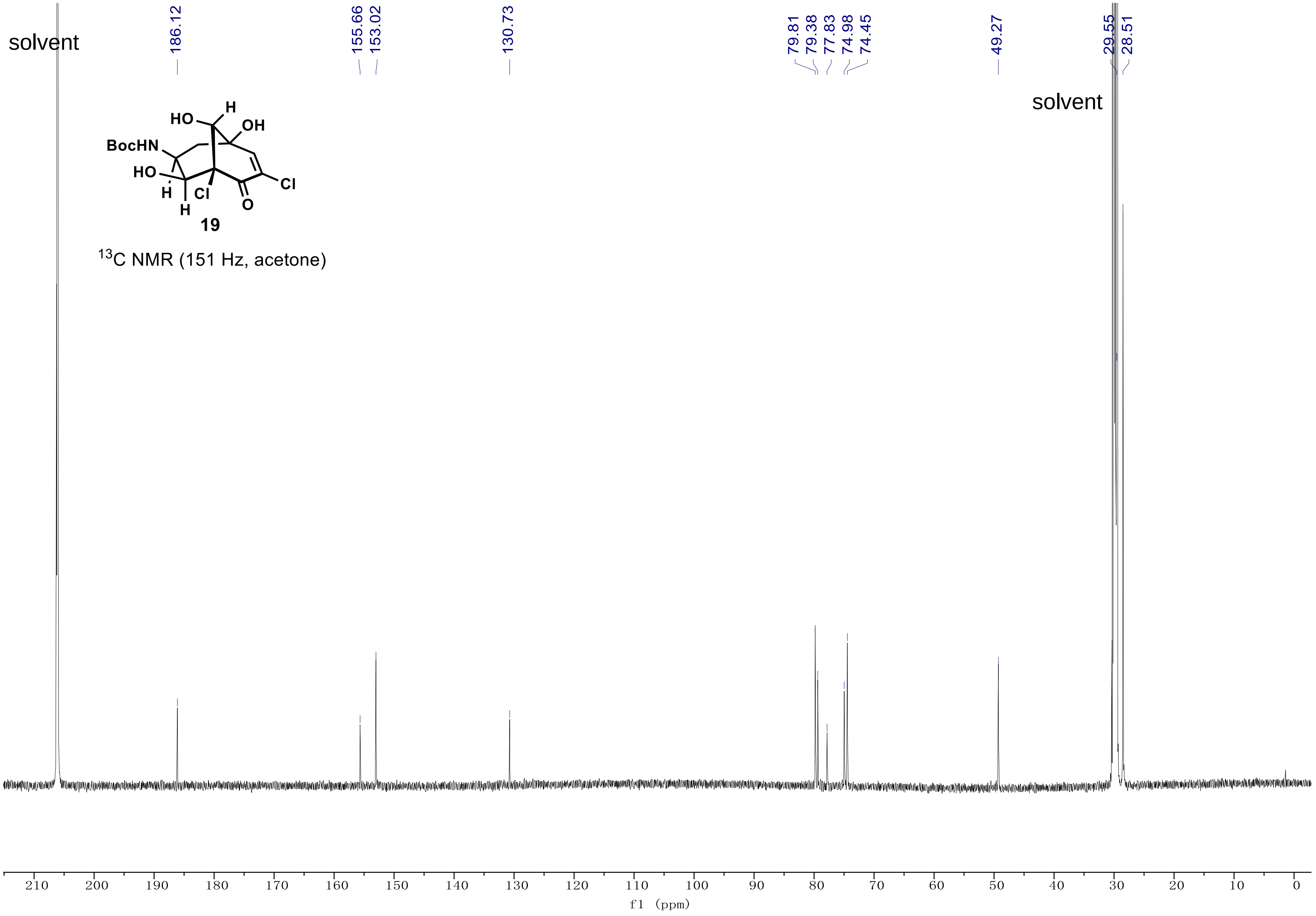
solvent
186.12
155.66
153.02
130.73
79.81
79.38
77.83
74.98
74.45
49.27
29.55
28.51
solvent
HO
H
OH
BocHN
HO
H
Cl
Cl
H
O
19
^{13}C NMR (151 Hz, acetone)
210 200 190 180 170 160 150 140 130 120 110 100 90 80 70 60 50 40 30 20 10 0
f1 (ppm)

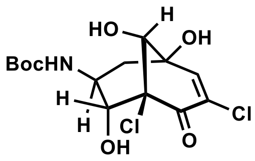

20

^{1}H NMR (600 MHz, acetone)

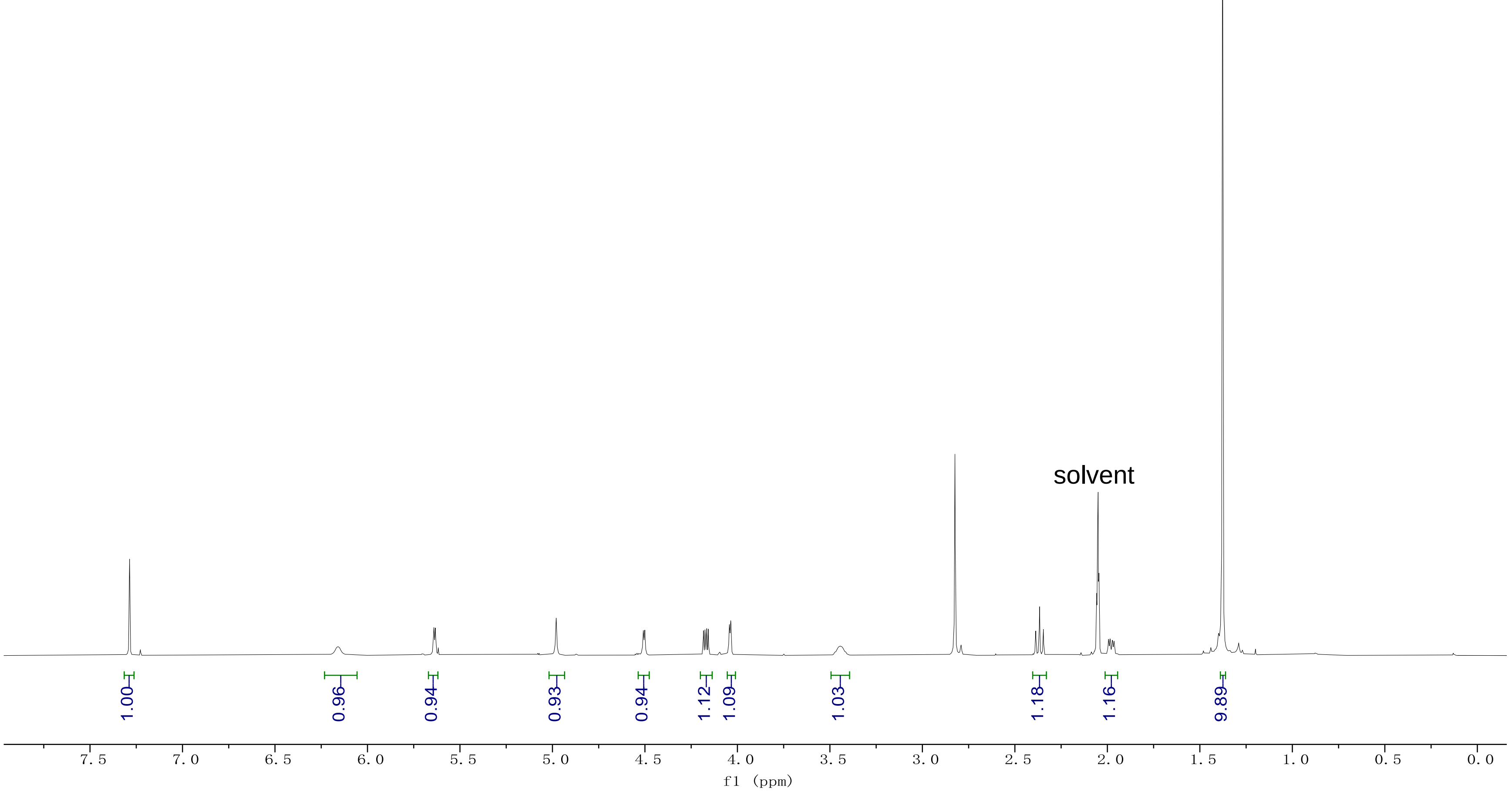

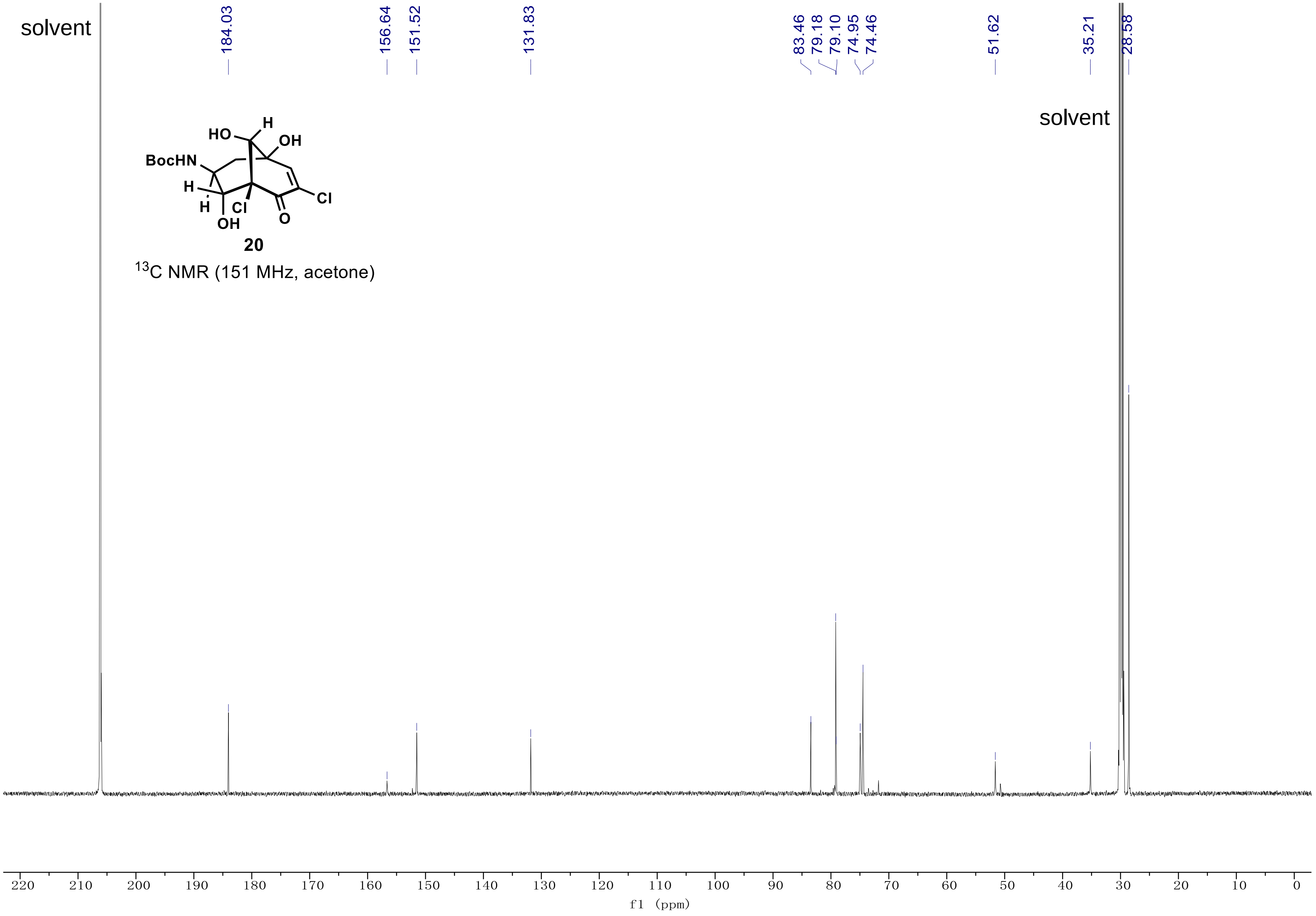
solvent
184.03
156.64
151.52
131.83
83.46
79.18
79.10
74.95
74.46
51.62
35.21
28.58
solvent
HO
H
OH
BocHN
H
H
Cl
Cl
O
OH
20
^{13}C NMR (151 MHz, acetone)
220 210 200 190 180 170 160 150 140 130 120 110 100 90 80 70 60 50 40 30 20 10 0
f1 (ppm)

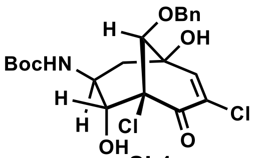

SI-1

^{1}H NMR (700 MHz, acetone)

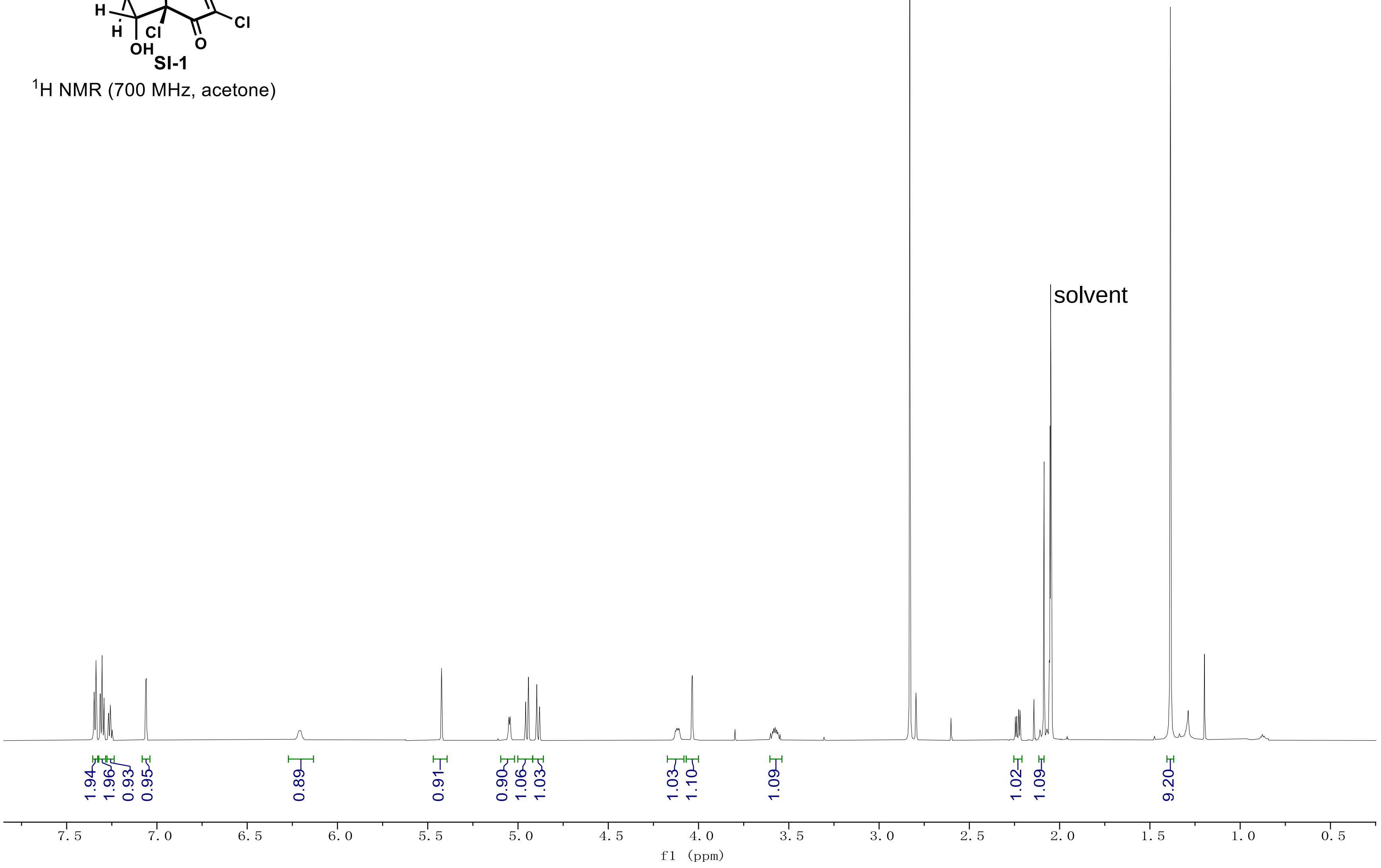

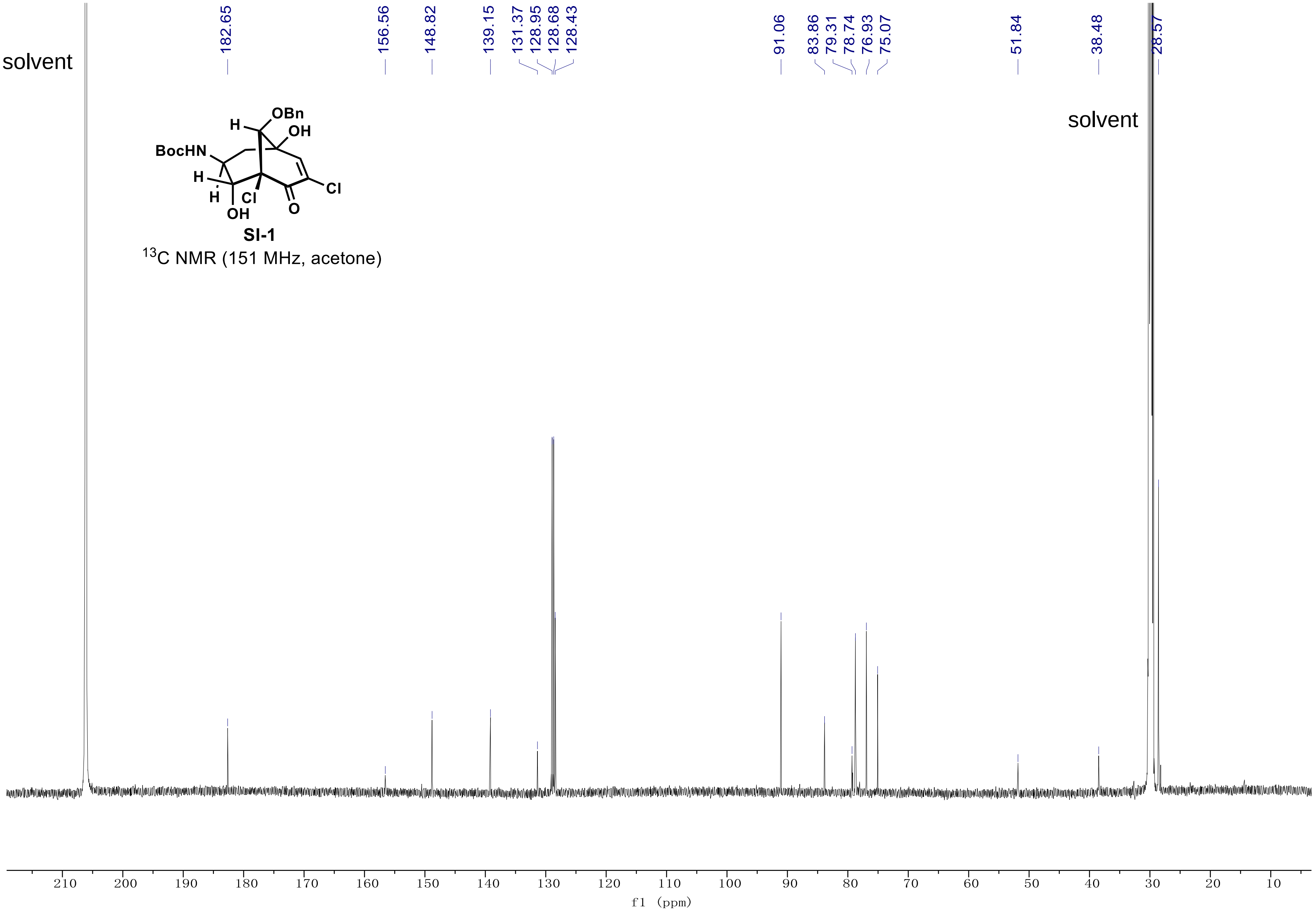
solvent
182.65
156.56
148.82
139.15
131.37
128.95
128.68
128.43
91.06
83.86
79.31
78.74
76.93
75.07
51.84
38.48
28.57
OBn
OH
BocHN
H
H
Cl
Cl
OH
O
SI-1
^{13}C NMR (151 MHz, acetone)
solvent
210
200
190
180
170
160
150
140
130
120
110
100
90
80
70
60
50
40
30
20
10
f1 (ppm)

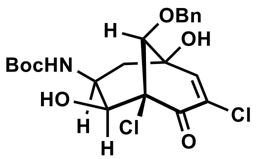

SI-2

^{1}H NMR (700 MHz, acetone)

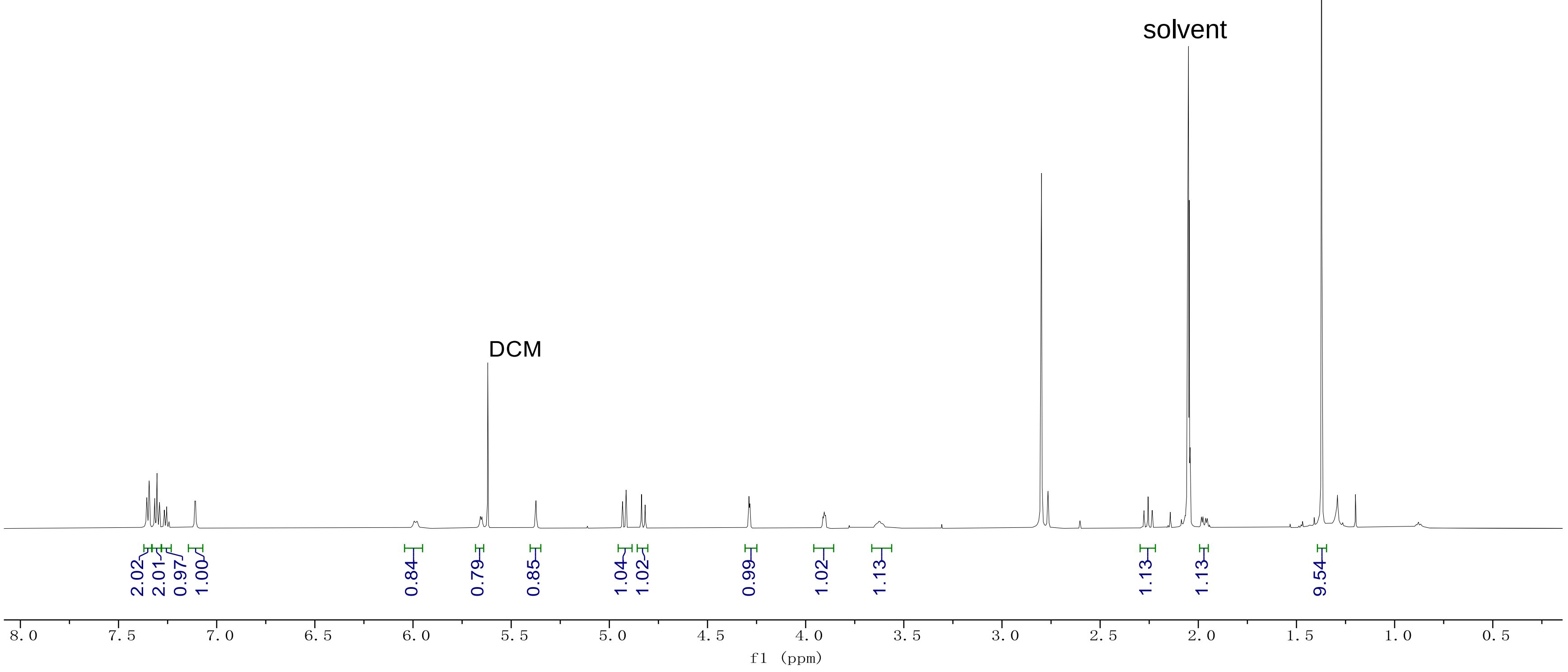

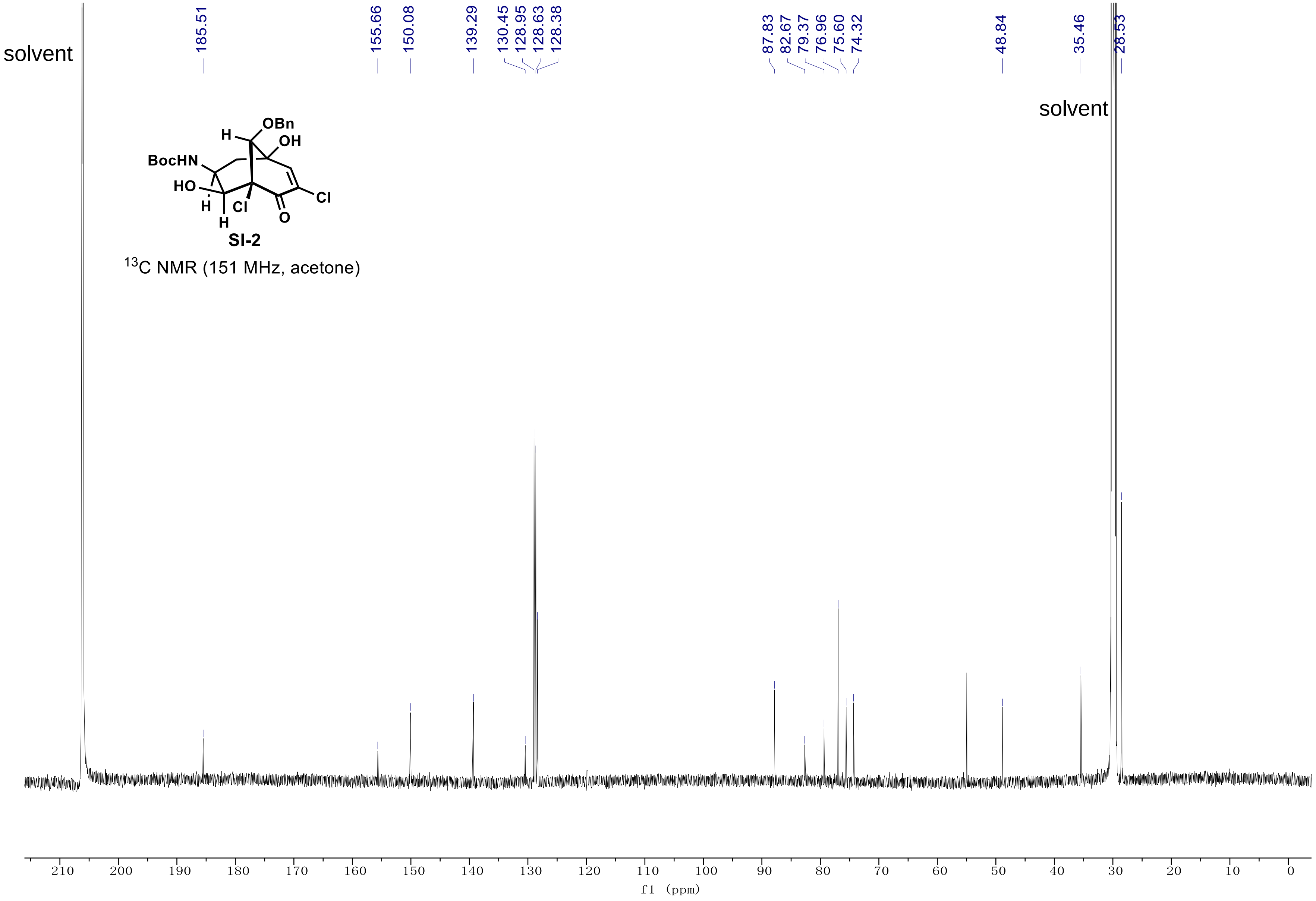
solvent
185.51
155.66
150.08
139.29
130.45
128.95
128.63
128.38
87.83
82.67
79.37
76.96
75.60
74.32
48.84
35.46
28.53
solvent
OBn
H
OH
BocHN
HO
H
Cl
Cl
H
O
SI-2
^{13}C NMR (151 MHz, acetone)
210 200 190 180 170 160 150 140 130 120 110 100 90 80 70 60 50 40 30 20 10 0
f1 (ppm)

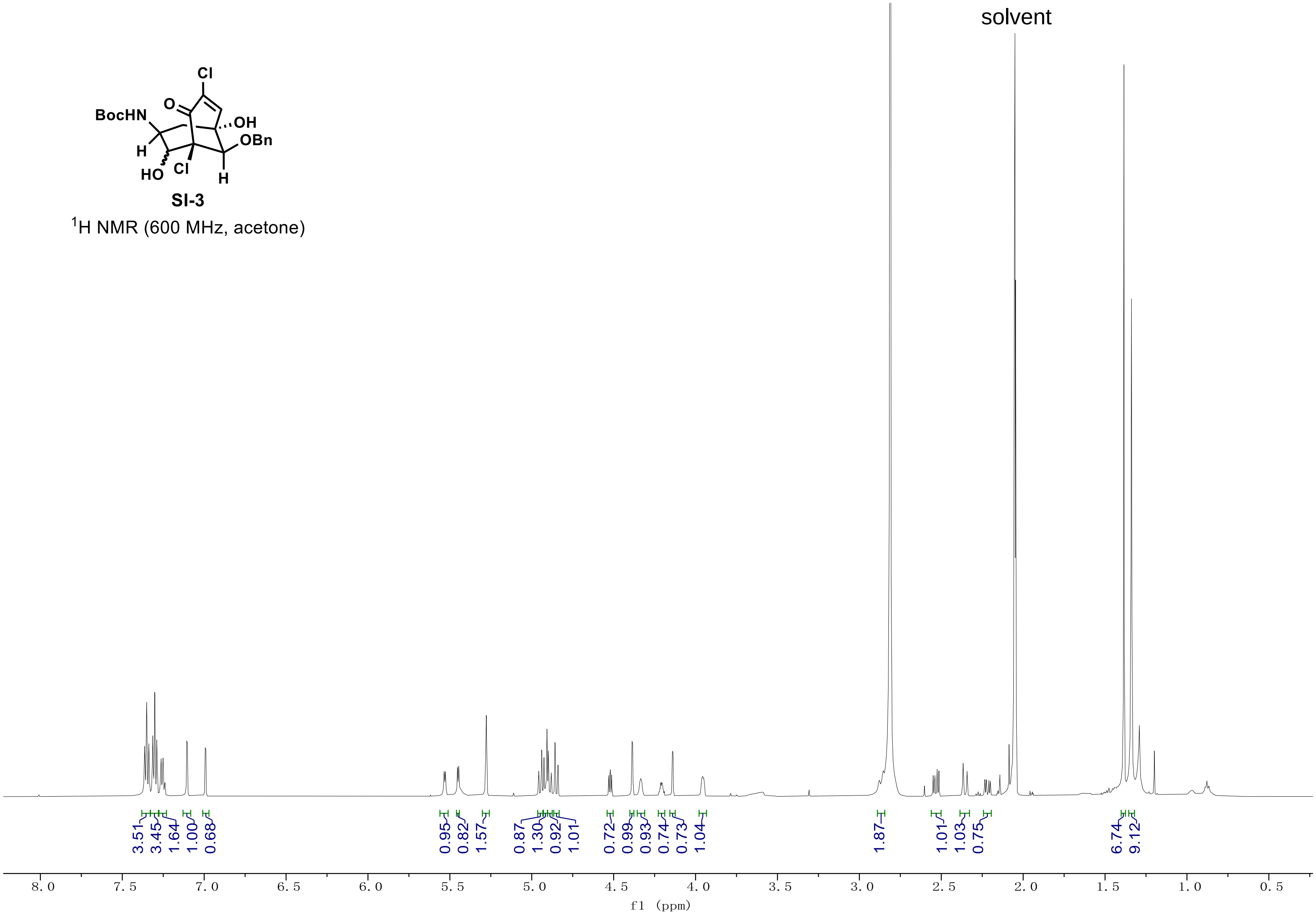

SI-3

^{1}H NMR (600 MHz, acetone)

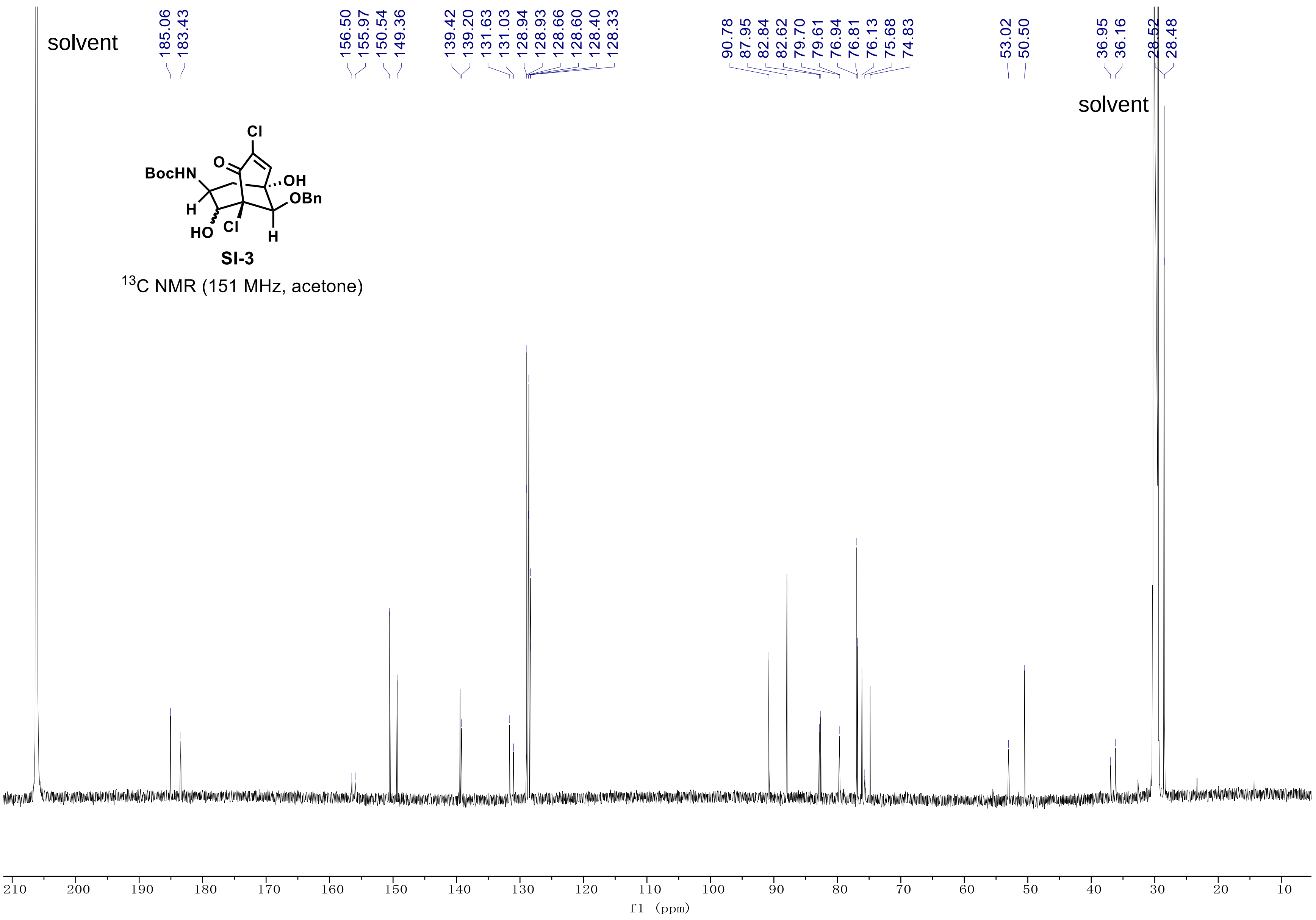
solvent
185.06
183.43
156.50
155.97
150.54
149.36
139.42
139.20
131.63
131.03
128.94
128.93
128.66
128.60
128.40
128.33
90.78
87.95
82.84
82.62
79.70
79.61
76.94
76.81
76.13
75.68
74.83
53.02
50.50
36.95
36.16
28.52
28.48
solvent
BocHN
O
Cl
OH
OBn
H
HO
Cl
H
SI-3
^{13}C NMR (151 MHz, acetone)
210
200
190
180
170
160
150
140
130
120
110
100
90
80
70
60
50
40
30
20
10
f1 (ppm)

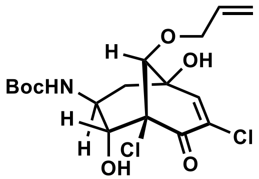

SI-4

^{1}H NMR (600 MHz, acetone)

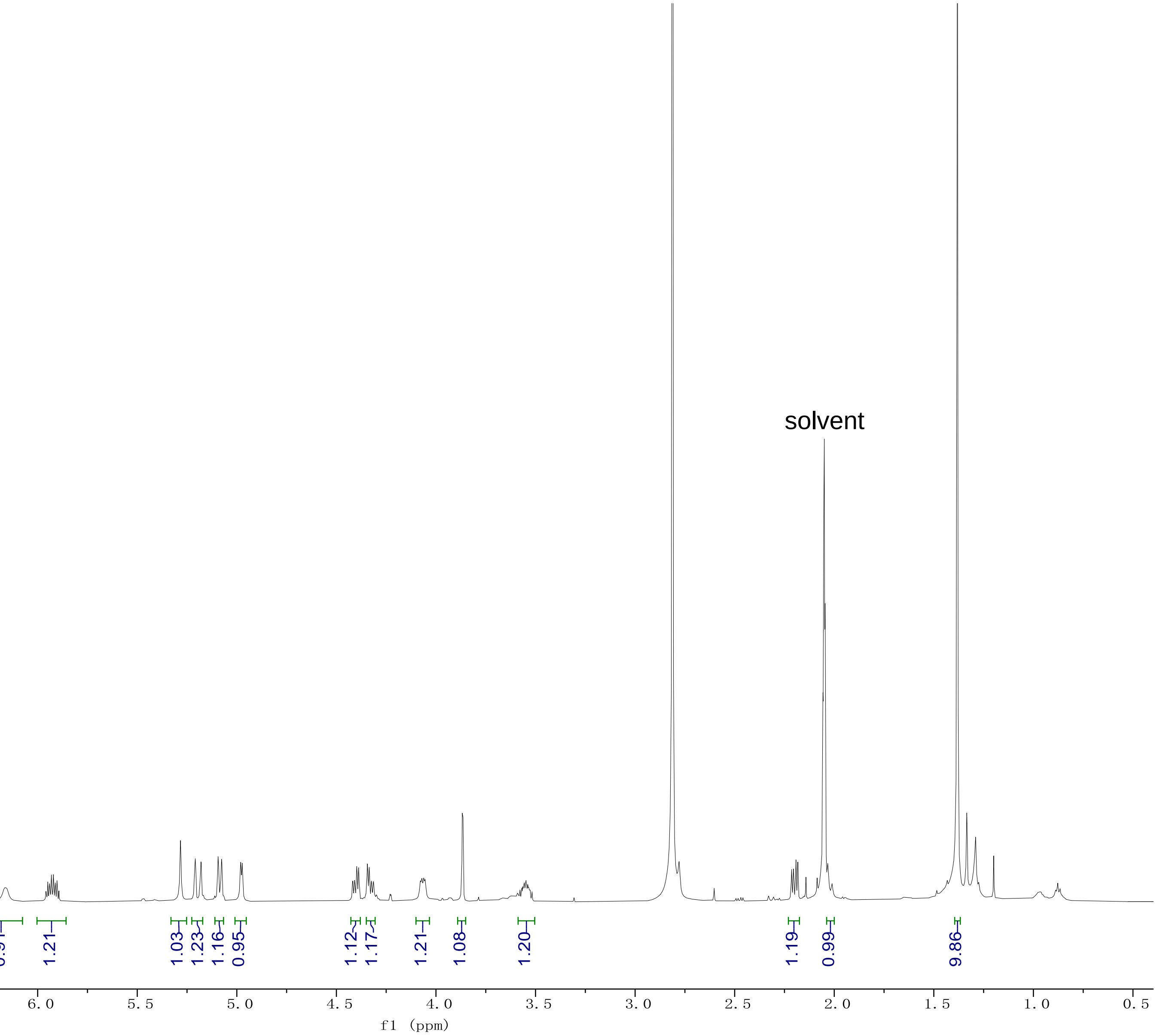

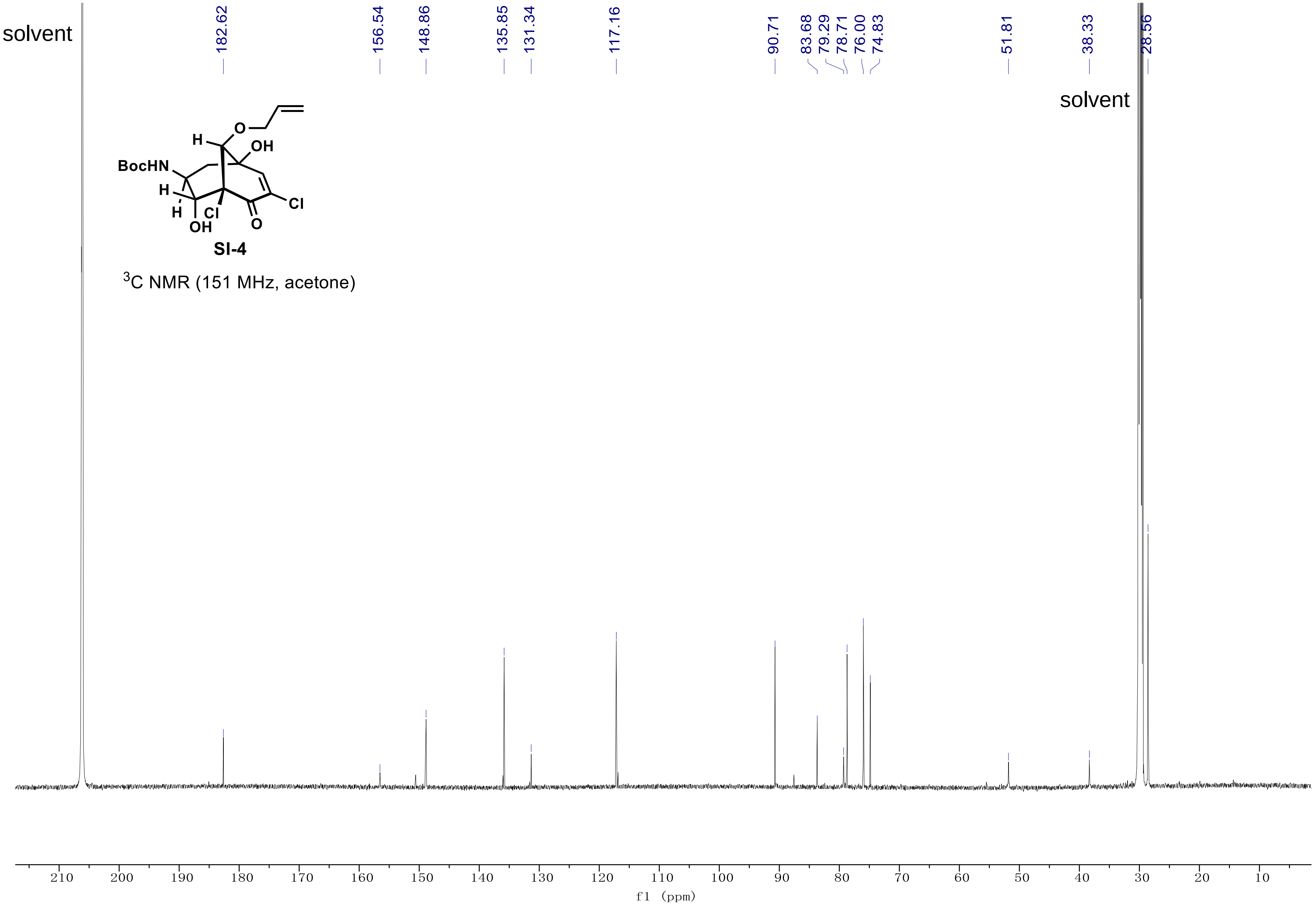
solvent
182.62
156.54
148.86
135.85
131.34
117.16
90.71
83.68
79.29
78.71
76.00
74.83
51.81
38.33
28.56
solvent
BocHN
OH
OH
Cl
Cl
O
H
SI-4
3C NMR (151 MHz, acetone)
210
200
190
180
170
160
150
140
130
120
110
100
90
80
70
60
50
40
30
20
10
f1 (ppm)

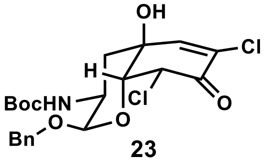

23

^{1}H NMR (600 MHz, acetone)

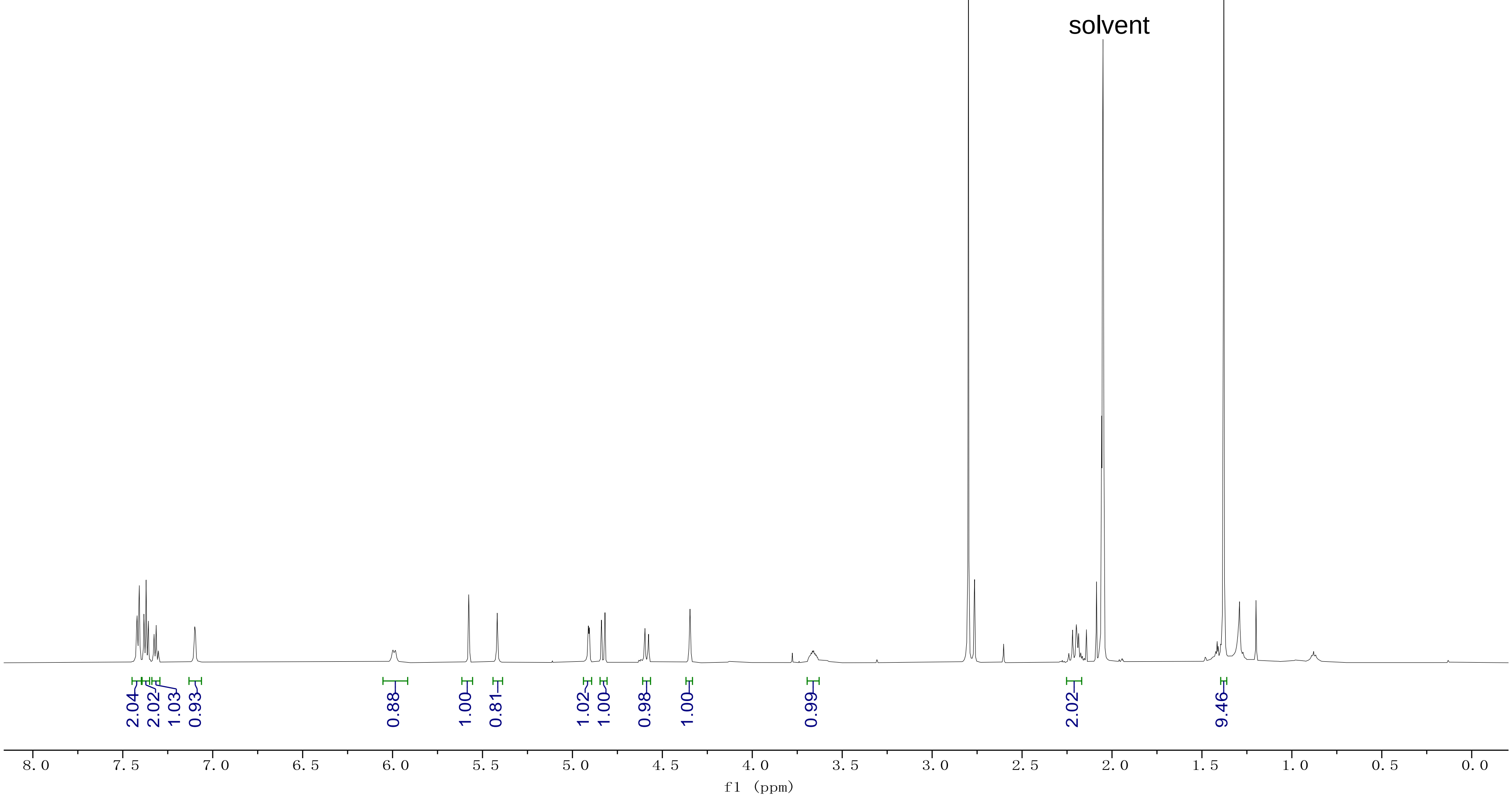

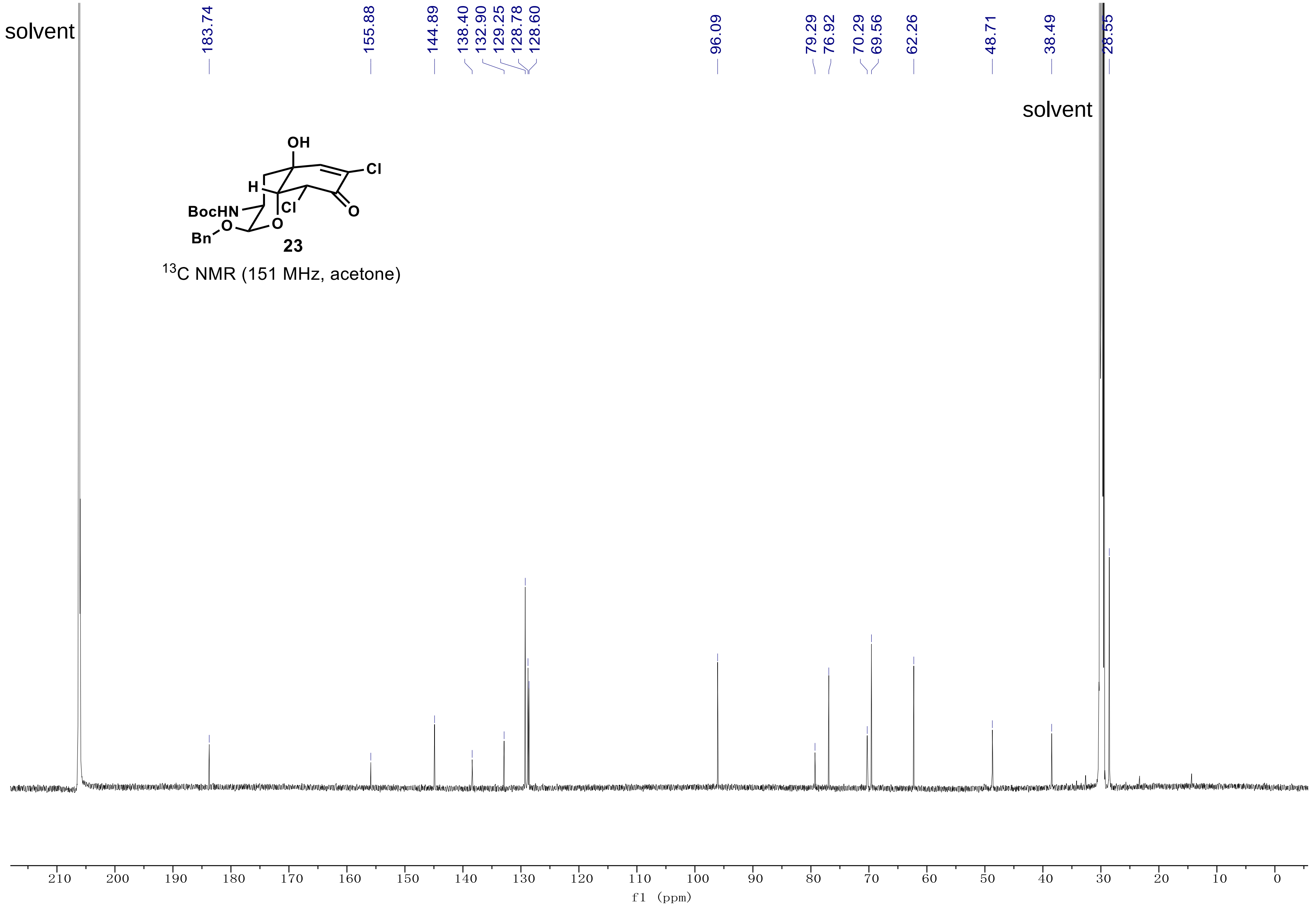

23

^{13}C NMR (151 MHz, acetone)

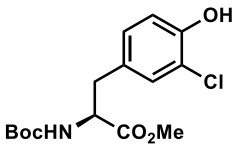

25

^{1}H NMR (600 MHz, $CDCl_3$)

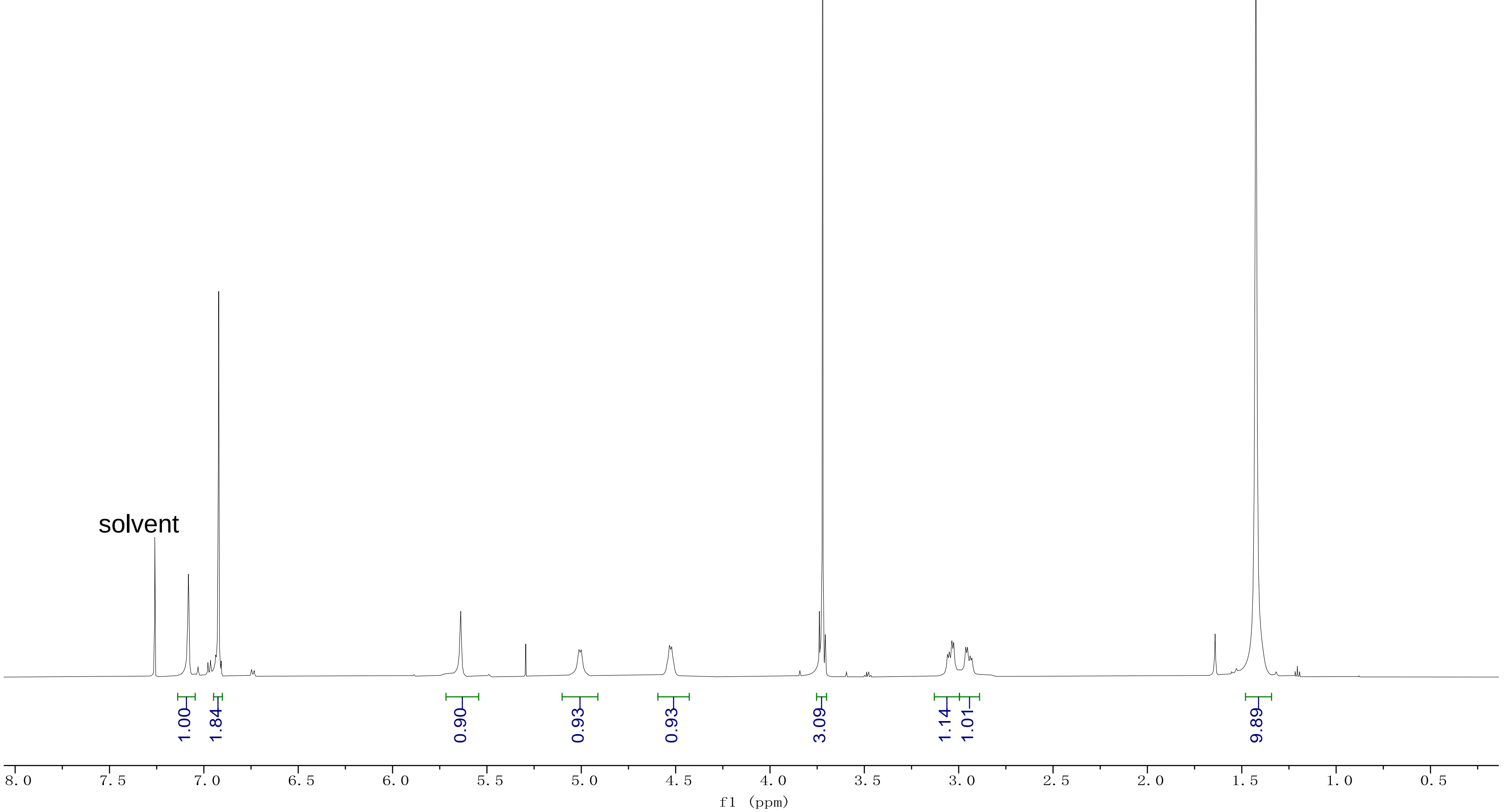

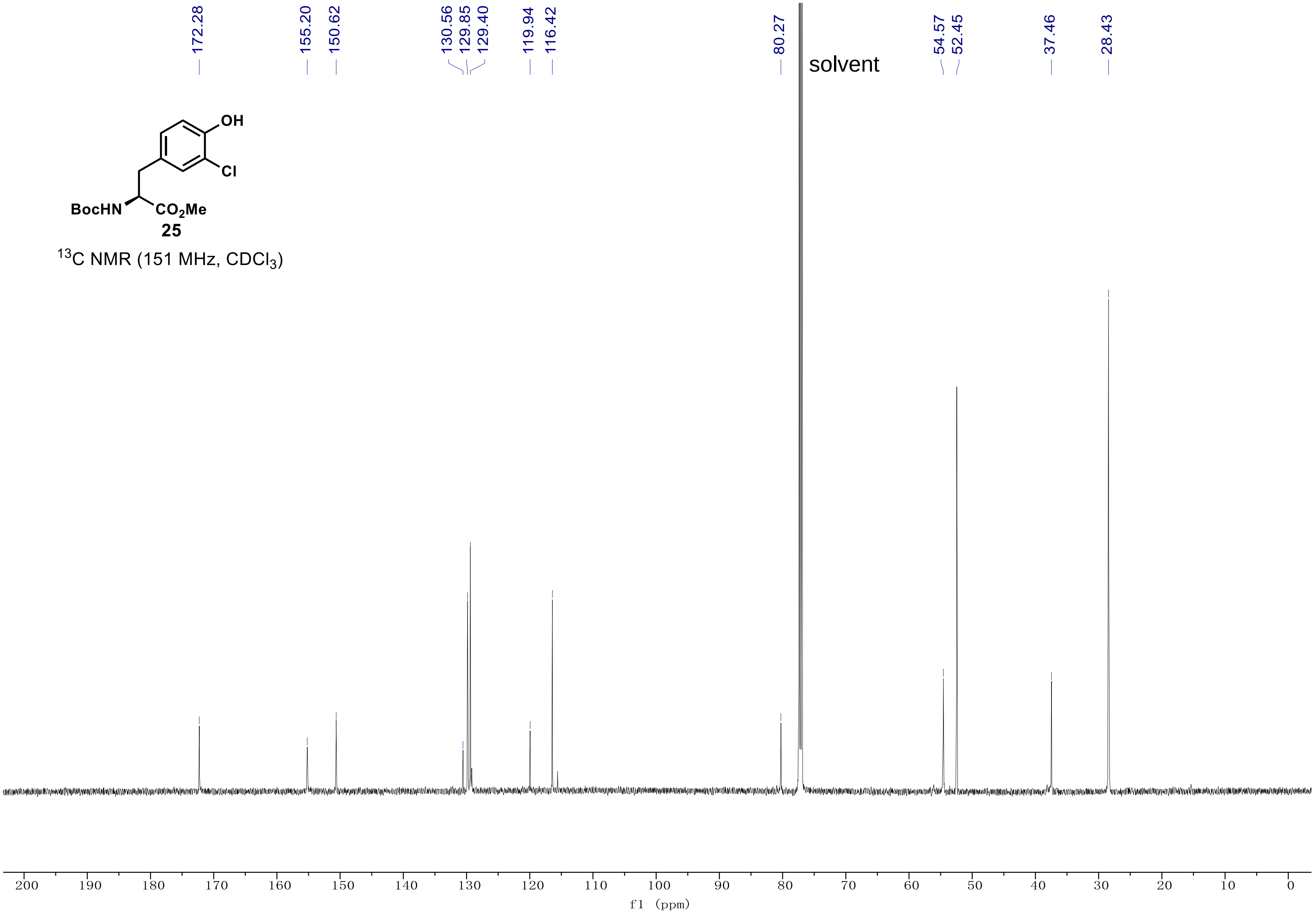

172.28
155.20
150.62
130.56
129.85
129.40
119.94
116.42
80.27
solvent
54.57
52.45
37.46
28.43
OH
Cl
BocHN
CO2Me
25
13C NMR (151 MHz, CDCl3)
200
190
180
170
160
150
140
130
120
110
100
90
80
70
60
50
40
30
20
10
0
f1 (ppm)

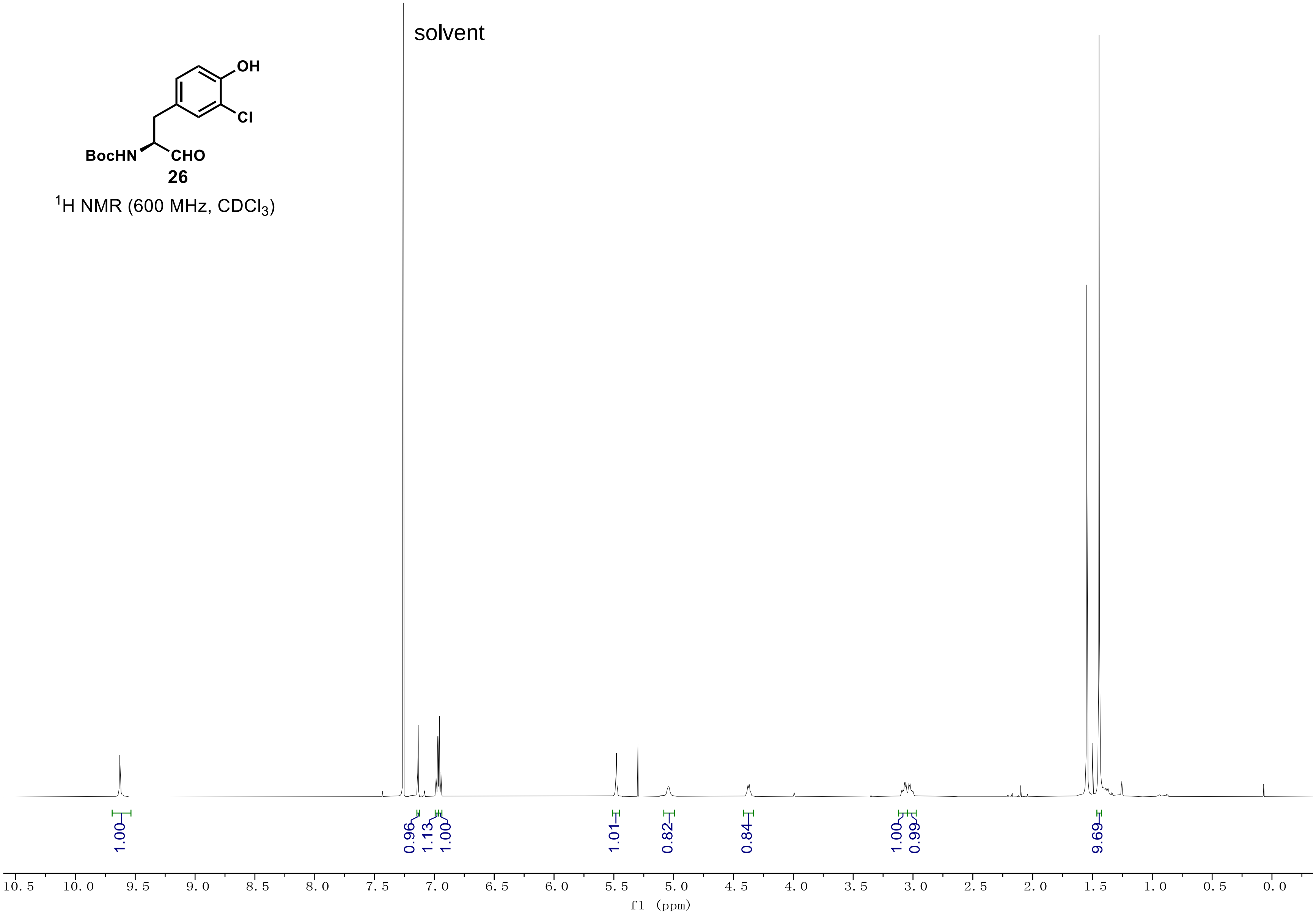
OH
Cl
BocHN
CHO
26
^{1}H NMR (600 MHz, $CDCl_3$)
solvent
1.00
0.96
1.13
1.00
1.01
0.82
0.84
1.00
0.99
9.69
10.5 10.0 9.5 9.0 8.5 8.0 7.5 7.0 6.5 6.0 5.5 5.0 4.5 4.0 3.5 3.0 2.5 2.0 1.5 1.0 0.5 0.0
f1 (ppm)

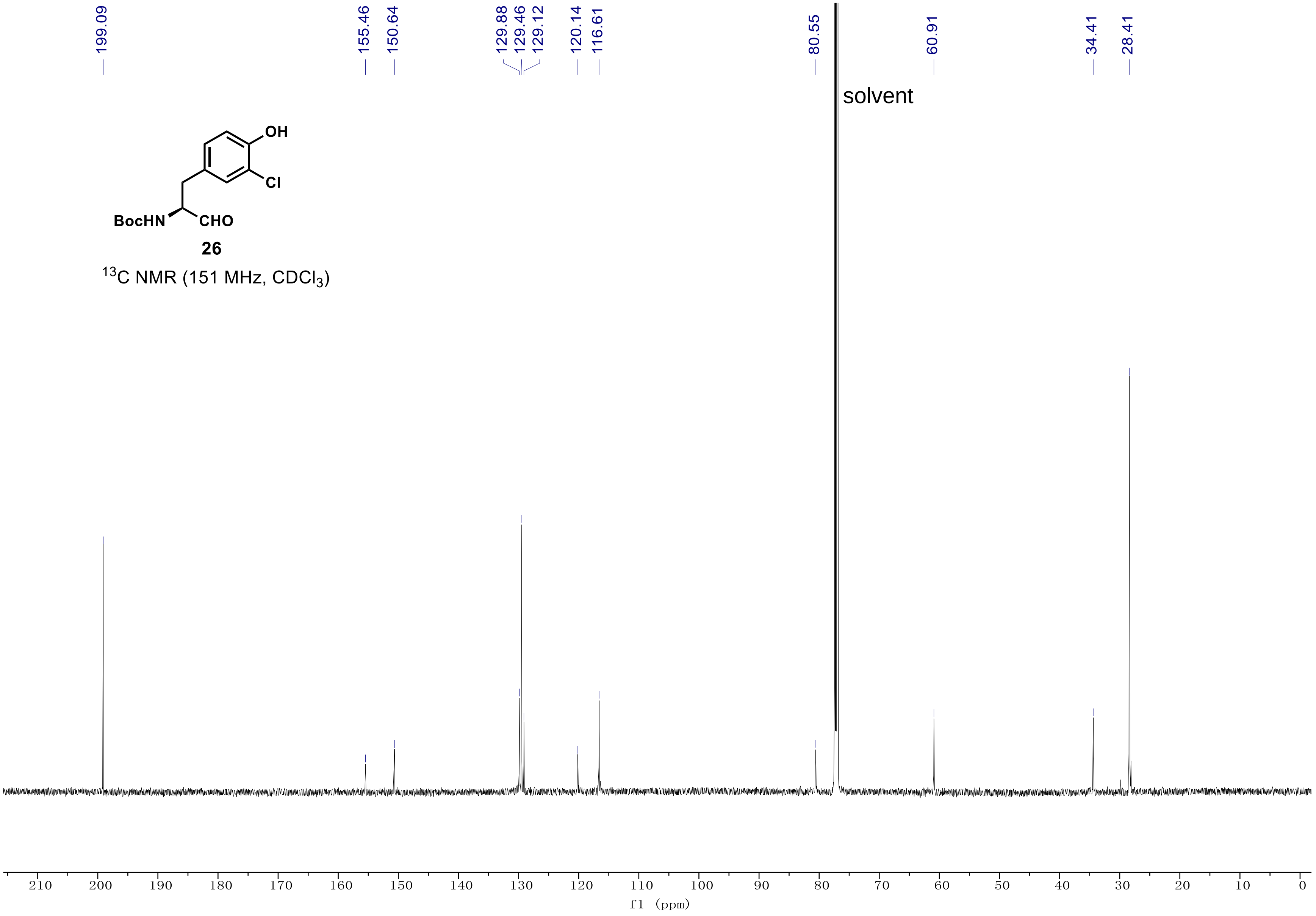

199.09
155.46
150.64
129.88
129.46
129.12
120.14
116.61
80.55
60.91
34.41
28.41
solvent
OH
Cl
BocHN
CHO
26
^{13}C NMR (151 MHz, $CDCl_3$)
210
200
190
180
170
160
150
140
130
120
110
100
90
80
70
60
50
40
30
20
10
0
f1 (ppm)

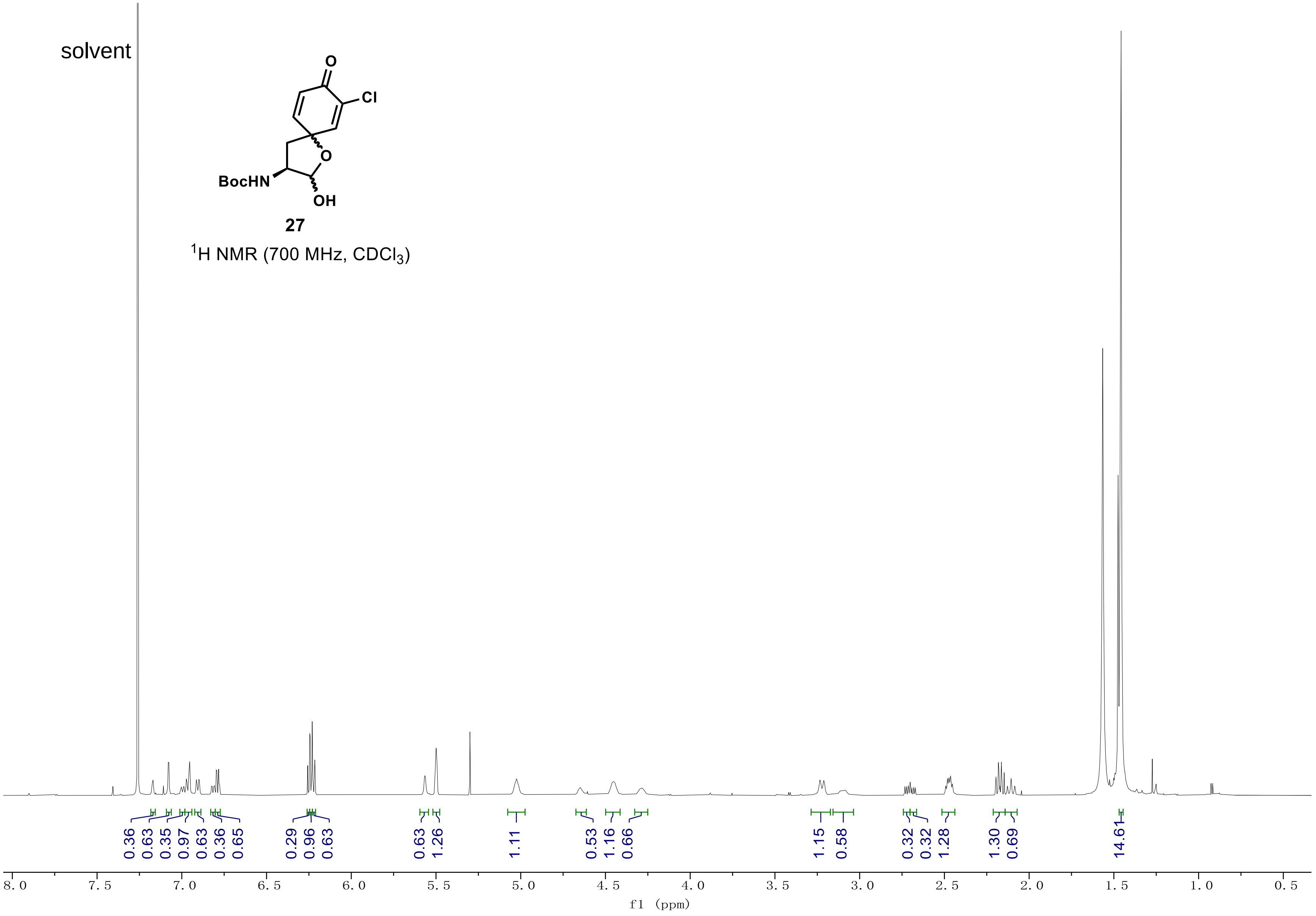
solvent
O
Cl
O
BocHN
OH
27
^{1}H NMR (700 MHz, CDCl$_3$)
0.36
0.63
0.35
0.97
0.63
0.36
0.65
0.29
0.96
0.63
0.63
1.26
1.11
0.53
1.16
0.66
1.15
0.58
0.32
0.32
1.28
1.30
0.69
14.61
8.0
7.5
7.0
6.5
6.0
5.5
5.0
4.5
4.0
3.5
3.0
2.5
2.0
1.5
1.0
0.5
f1 (ppm)

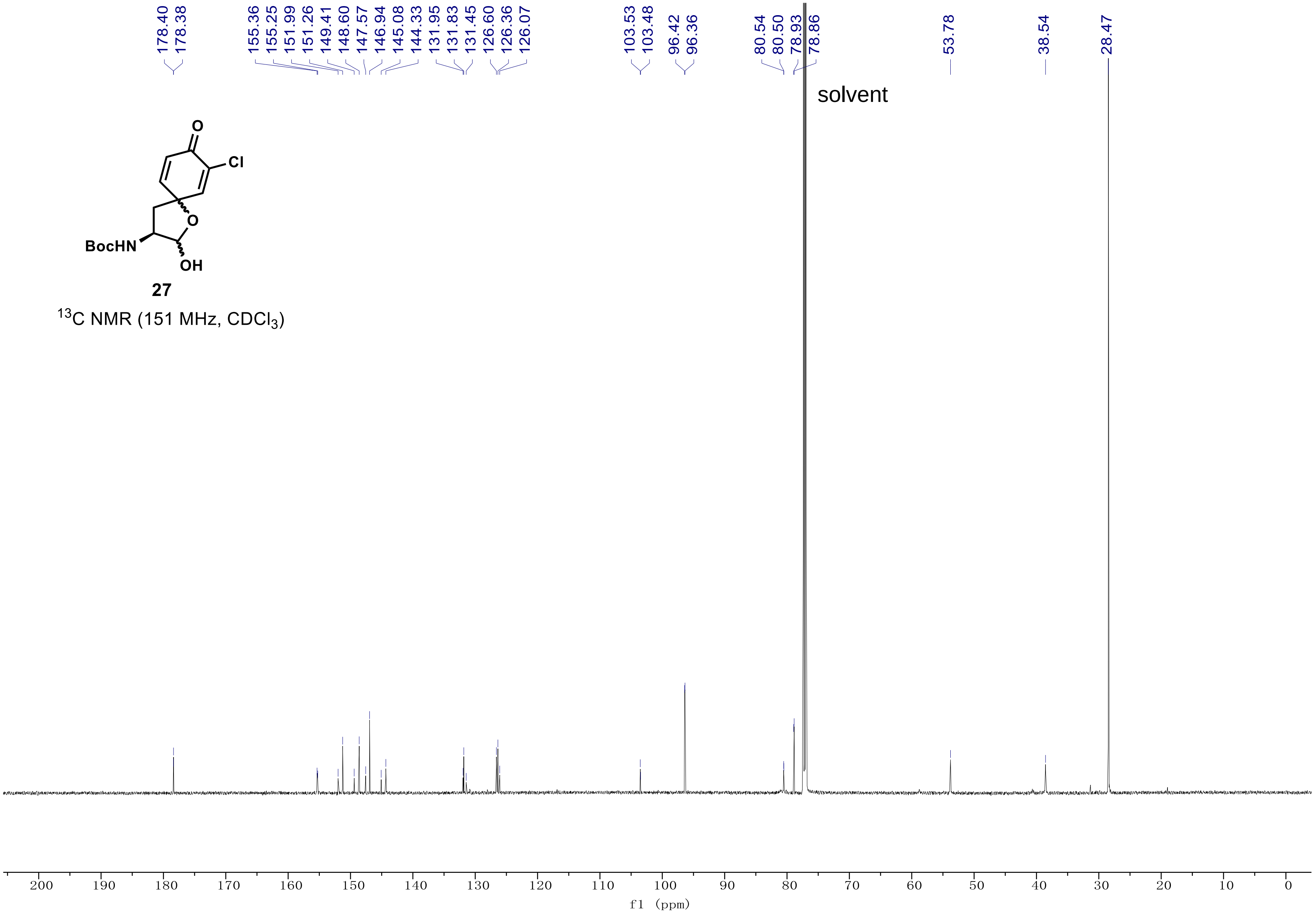
178.40
178.38
155.36
155.25
151.99
151.26
149.41
148.60
147.57
146.94
145.08
144.33
131.95
131.83
131.45
126.60
126.36
126.07
103.53
103.48
96.42
96.36
80.54
80.50
78.93
78.86
53.78
38.54
28.47
solvent
O
Cl
O
BocHN
OH
27
^{13}C NMR (151 MHz, CDCl$_3$)
200
190
180
170
160
150
140
130
120
110
100
90
80
70
60
50
40
30
20
10
0
f1 (ppm)

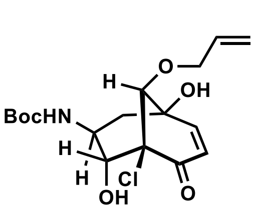

28a

^{1}H NMR (600 MHz, acetone)

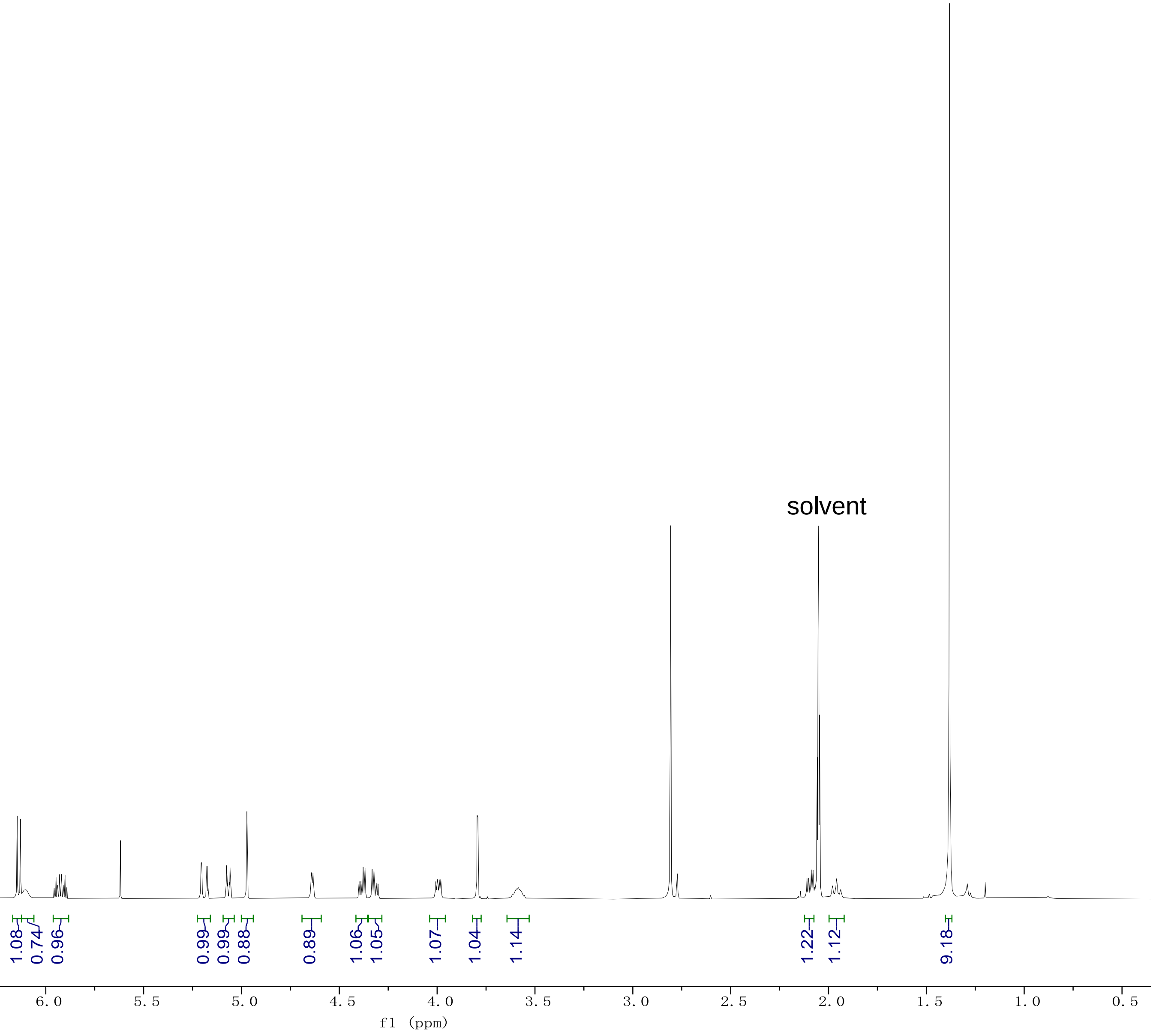

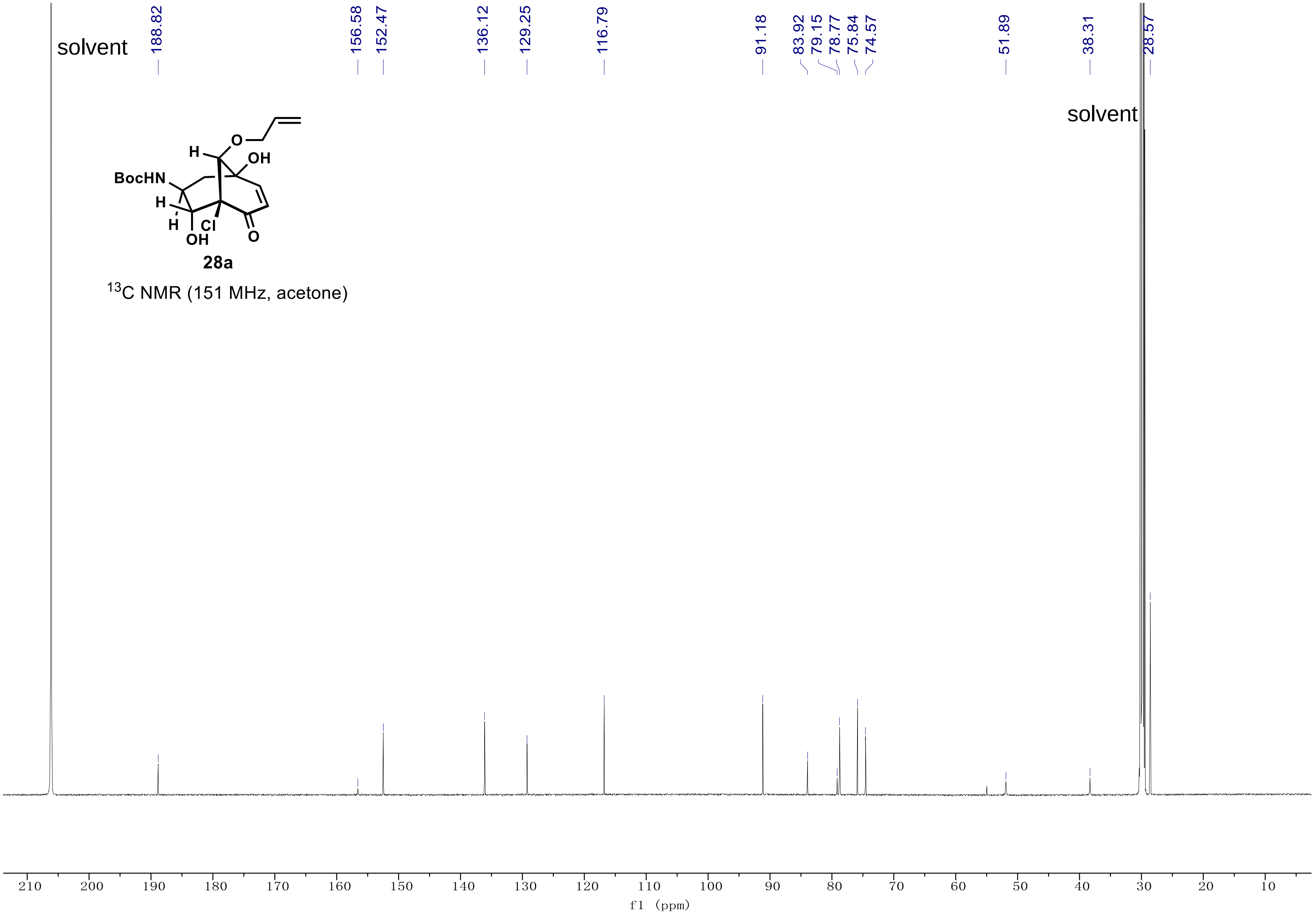
solvent
188.82
156.58
152.47
136.12
129.25
116.79
91.18
83.92
79.15
78.77
75.84
74.57
51.89
38.31
28.57
solvent
BocHN
OH
Cl
O
28a
^{13}C NMR (151 MHz, acetone)
210 200 190 180 170 160 150 140 130 120 110 100 90 80 70 60 50 40 30 20 10
f1 (ppm)

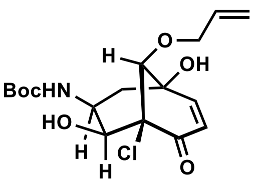

28b

^{1}H NMR (600 MHz, acetone)

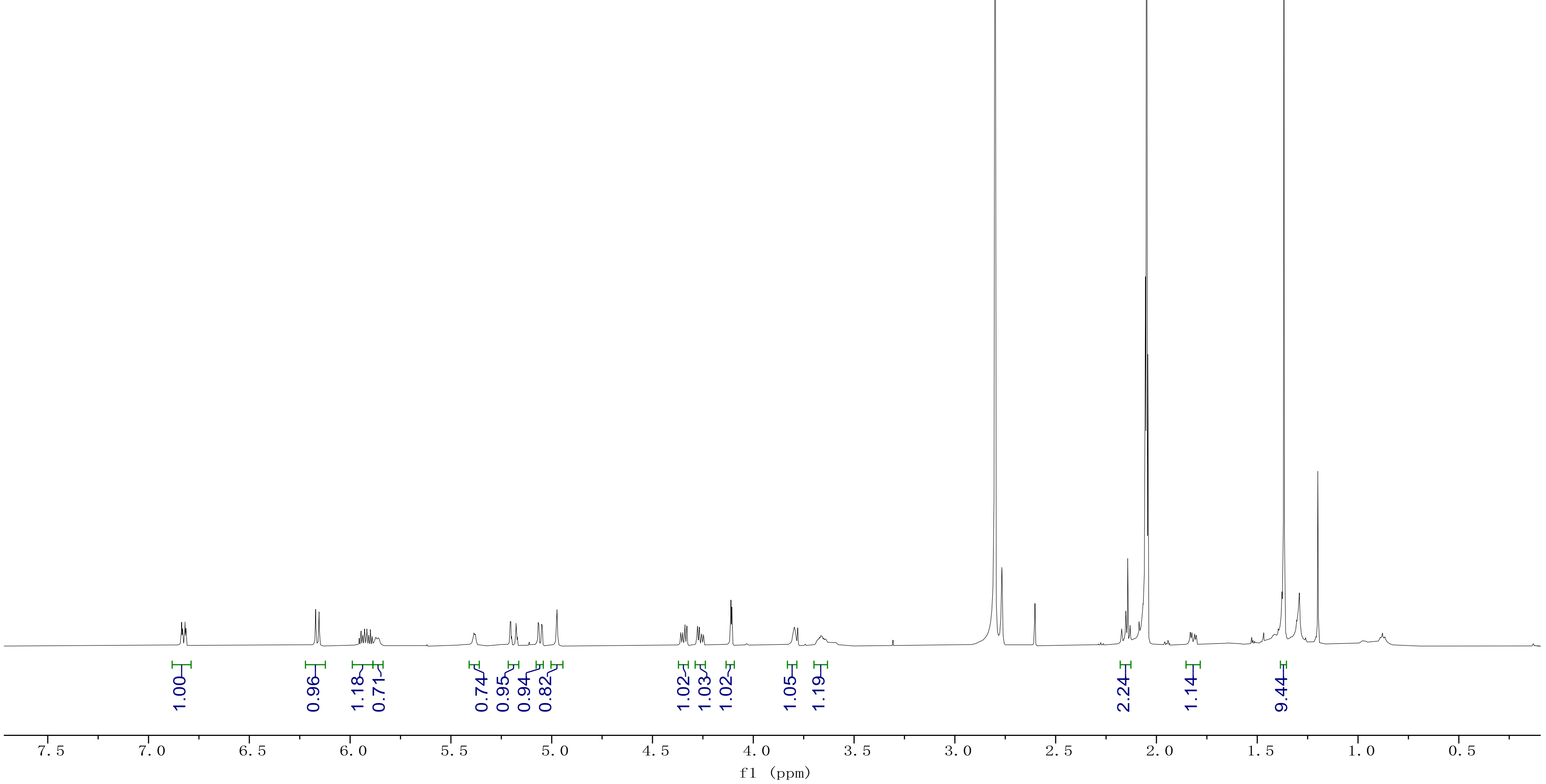

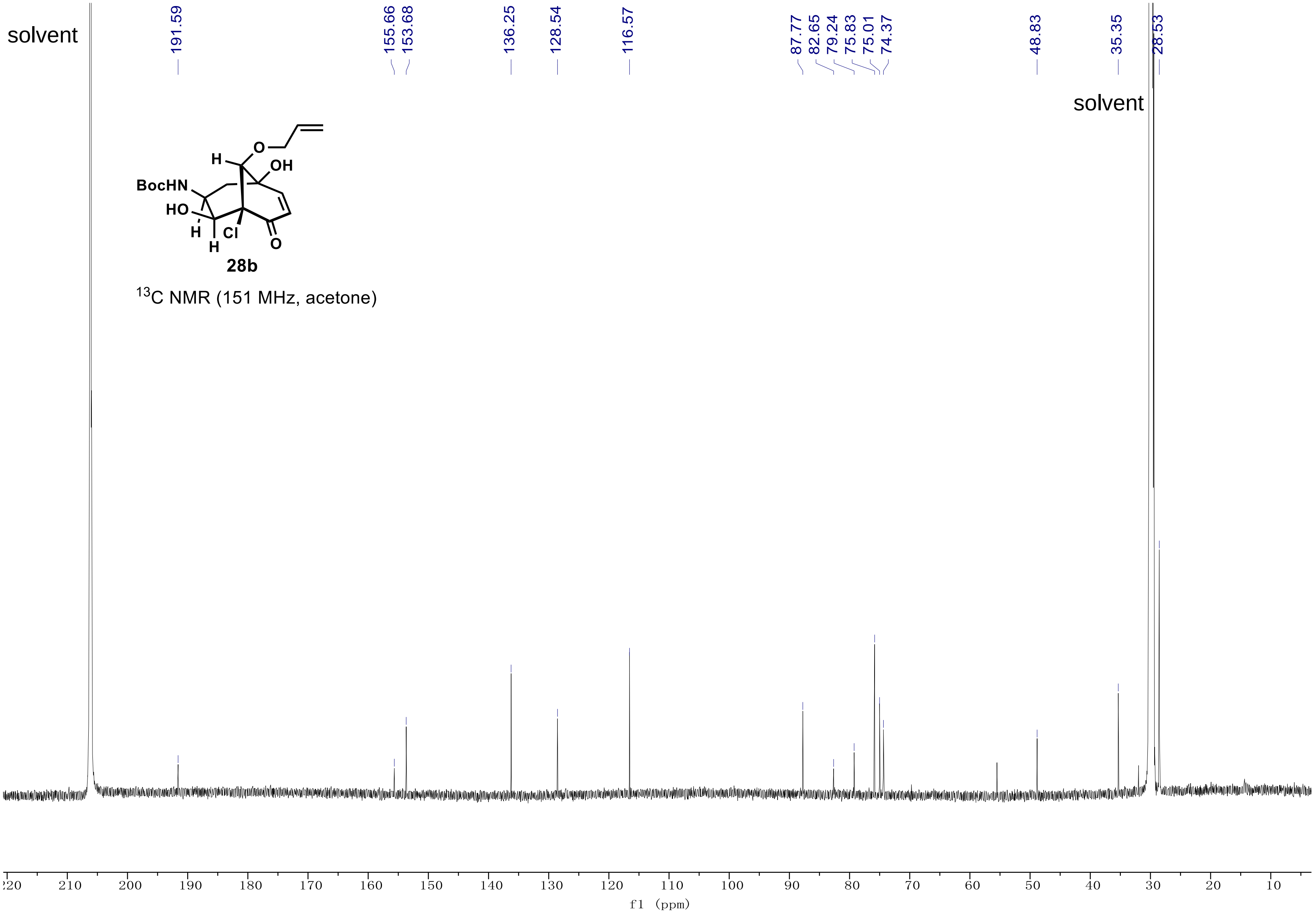

28b

^{13}C NMR (151 MHz, acetone)

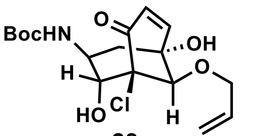

28c

^{1}H NMR (600 MHz, acetone)

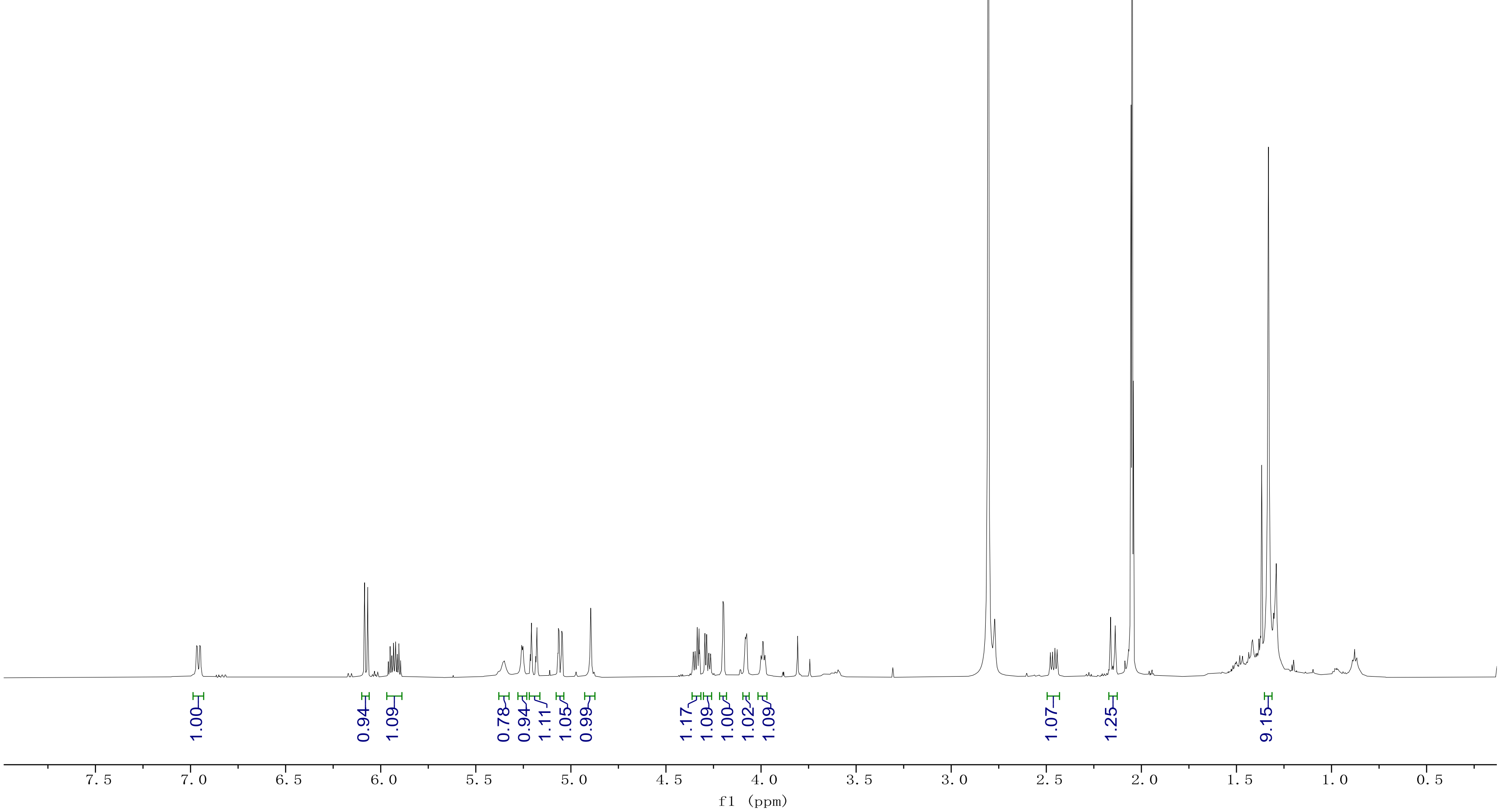

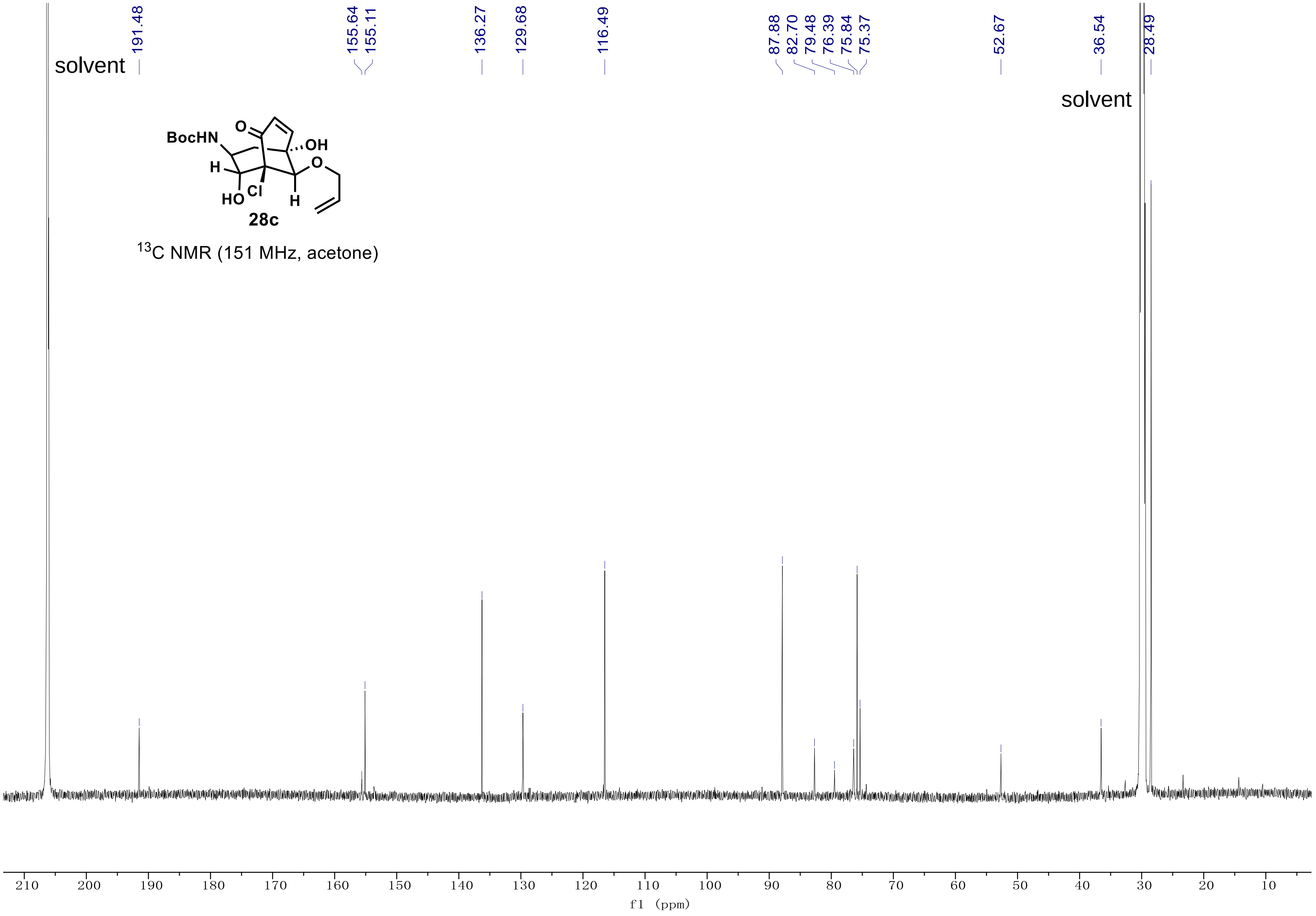

solvent
191.48
155.64
155.11
136.27
129.68
116.49
87.88
82.70
79.48
76.39
75.84
75.37
52.67
36.54
28.49
solvent
BocHN
O
OH
O
H
HO
Cl
H
28c
^{13}C NMR (151 MHz, acetone)
210 200 190 180 170 160 150 140 130 120 110 100 90 80 70 60 50 40 30 20 10
f1 (ppm)

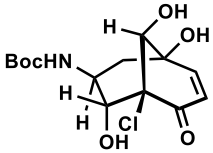

29

^{1}H NMR (600 MHz, acetone)

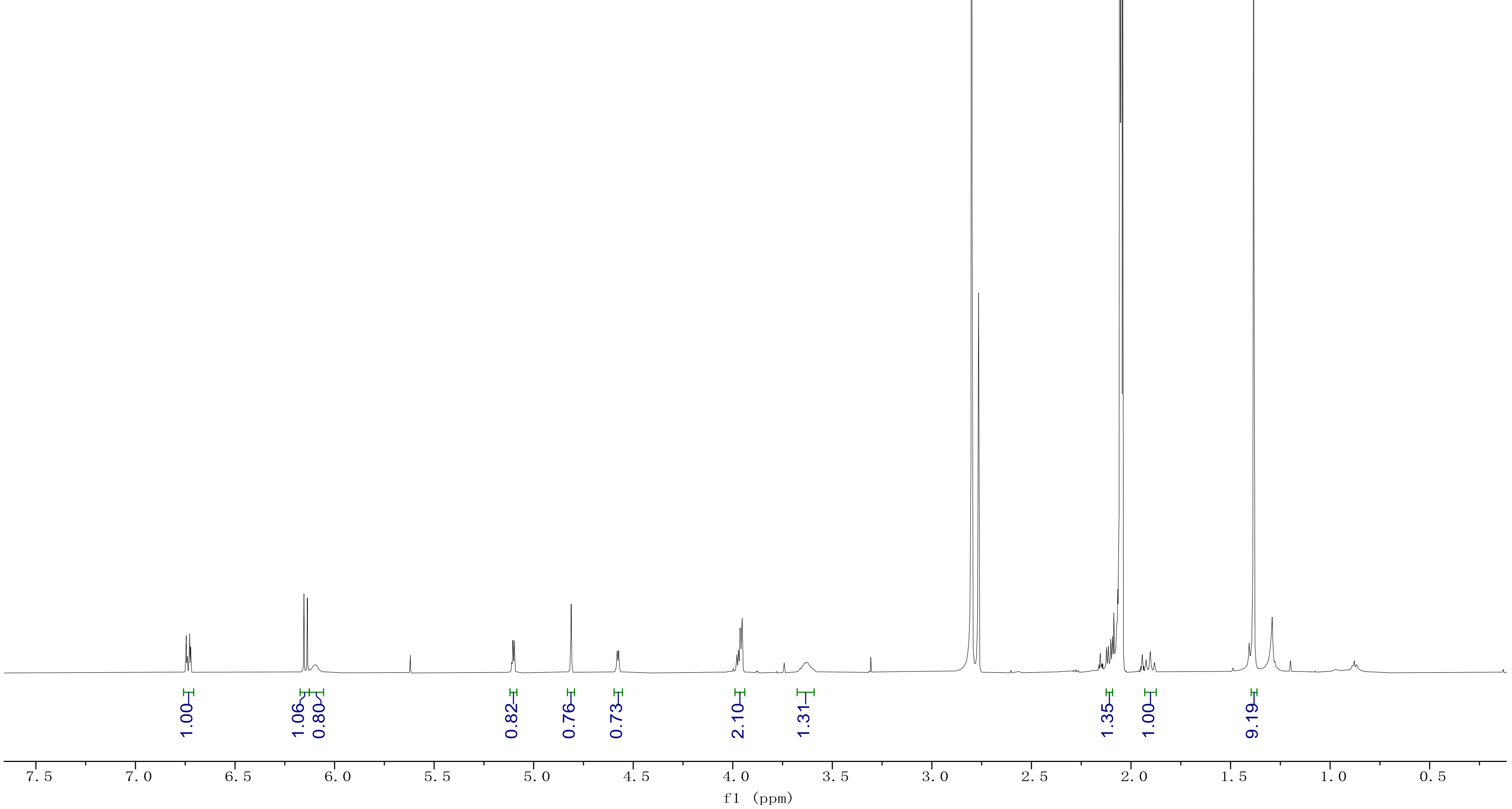

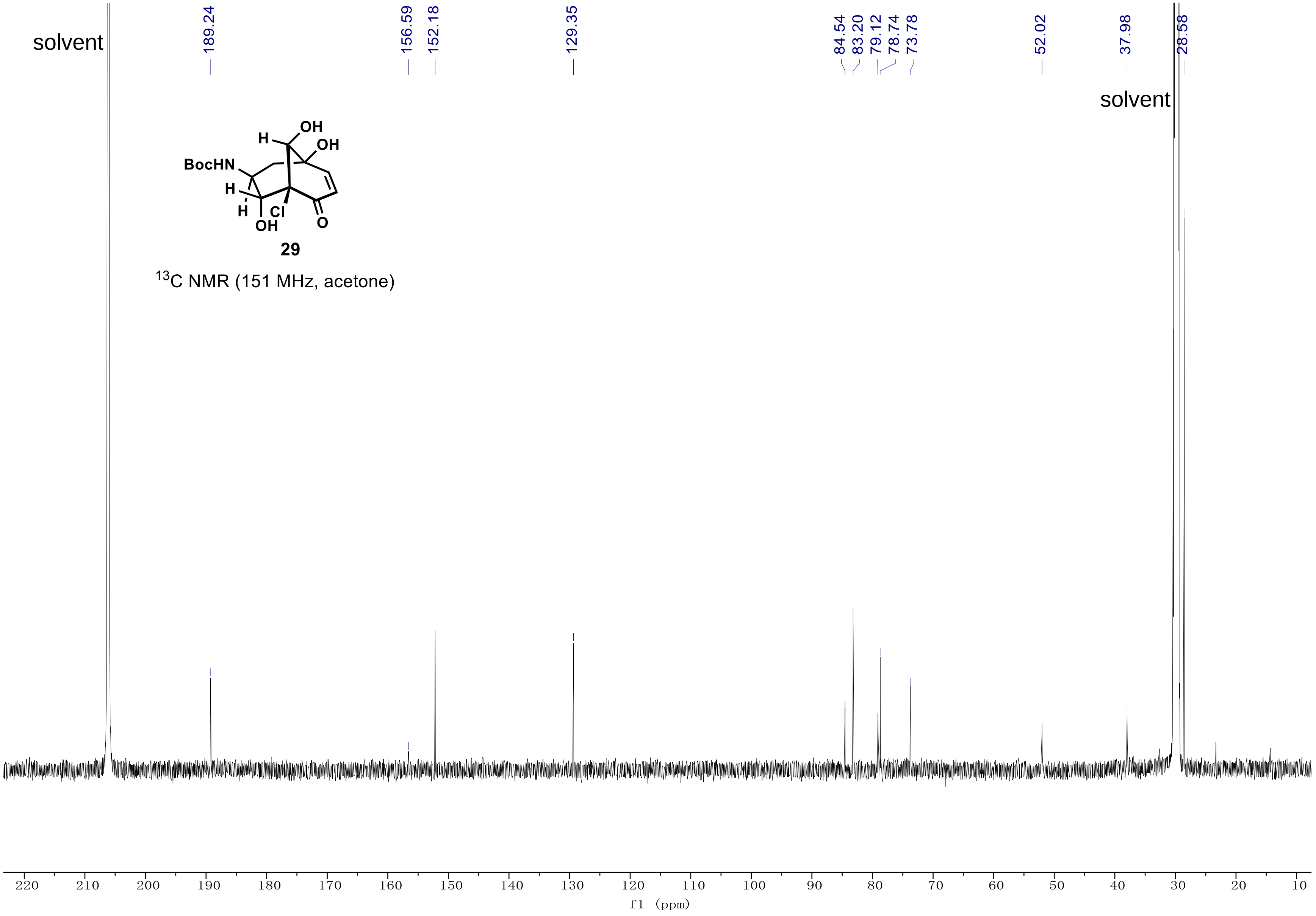

solvent
189.24
156.59
152.18
129.35
84.54
83.20
79.12
78.74
73.78
52.02
37.98
28.58
solvent
BocHN
OH
OH
H
H
H
Cl
OH
O
29
13C NMR (151 MHz, acetone)
220
210
200
190
180
170
160
150
140
130
120
110
100
90
80
70
60
50
40
30
20
10
f1 (ppm)

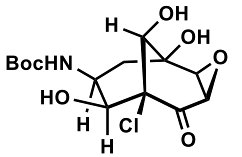

30a

^{1}H NMR (600 MHz, acetone)

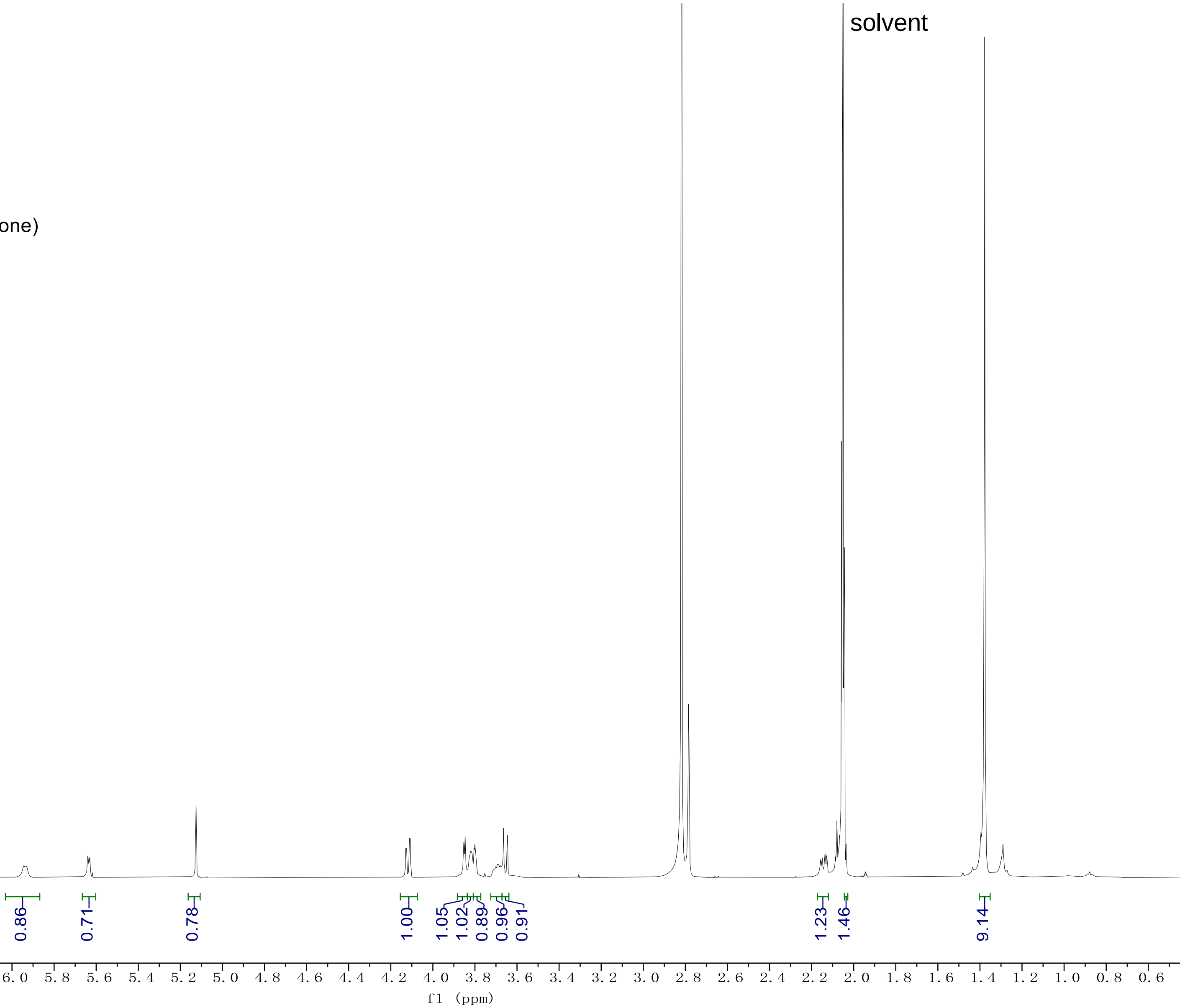

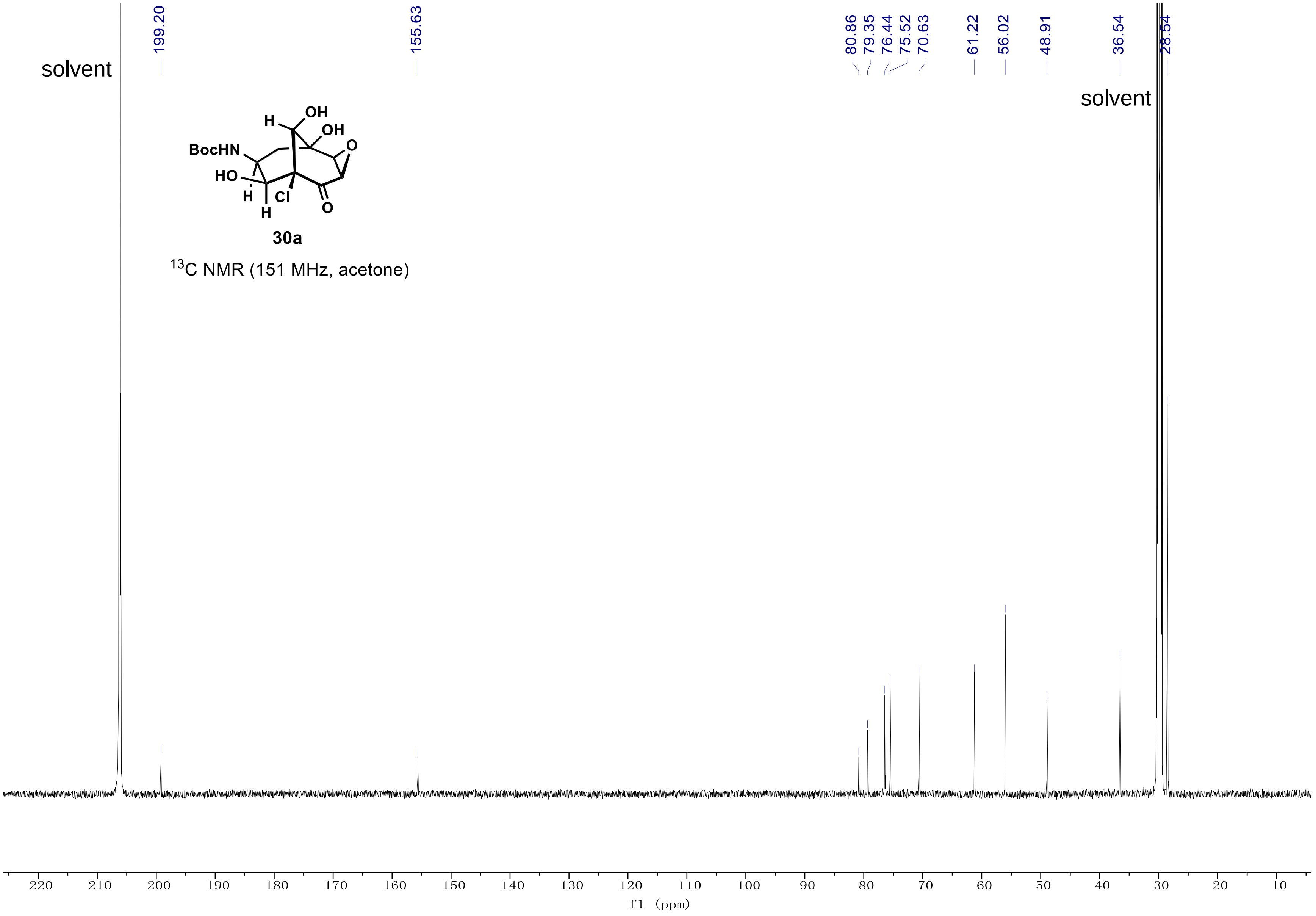
solvent
199.20
155.63
80.86
79.35
76.44
75.52
70.63
61.22
56.02
48.91
36.54
28.54
solvent
BocHN
HO
H
H
Cl
H
OH
OH
O
O
30a
^{13}C NMR (151 MHz, acetone)
220 210 200 190 180 170 160 150 140 130 120 110 100 90 80 70 60 50 40 30 20 10
f1 (ppm)

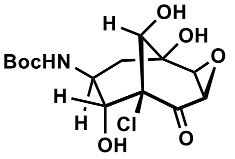

30b

^{1}H NMR (600 MHz, acetone)

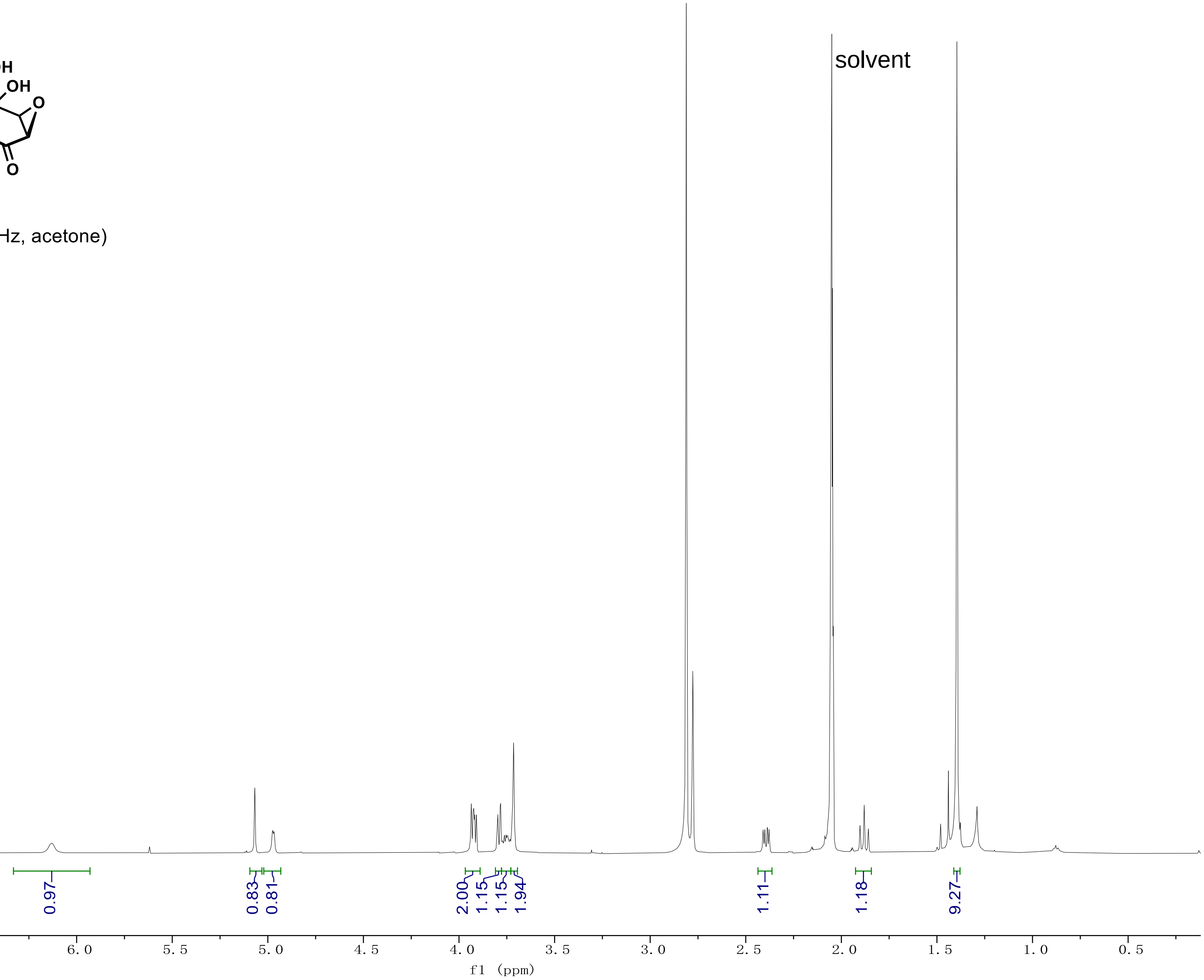

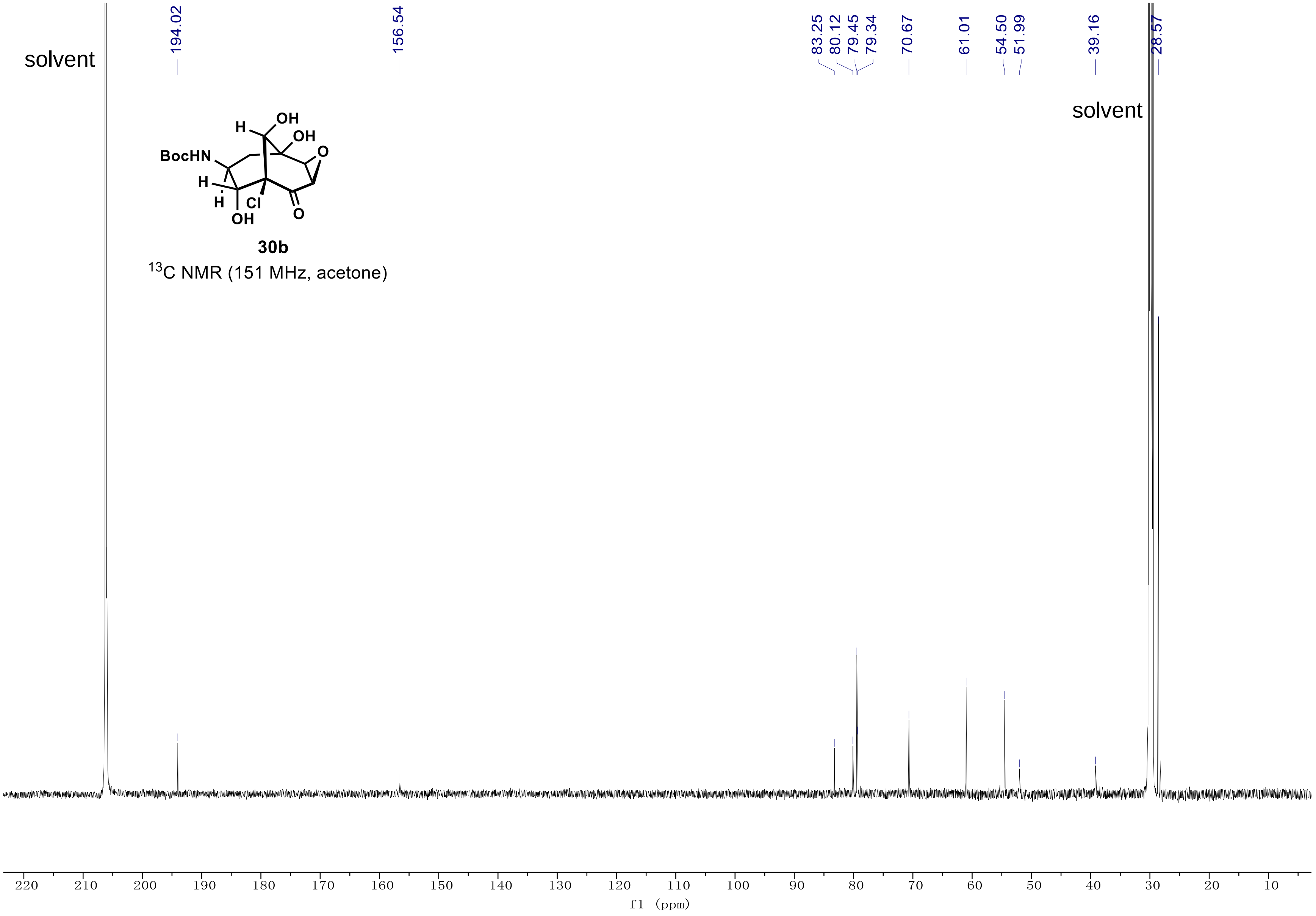

30b

^{13}C NMR (151 MHz, acetone)

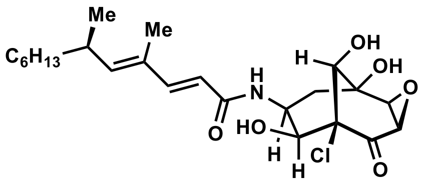

gymnastatin G (**11**)

^{1}H NMR (600 MHz, acetone)

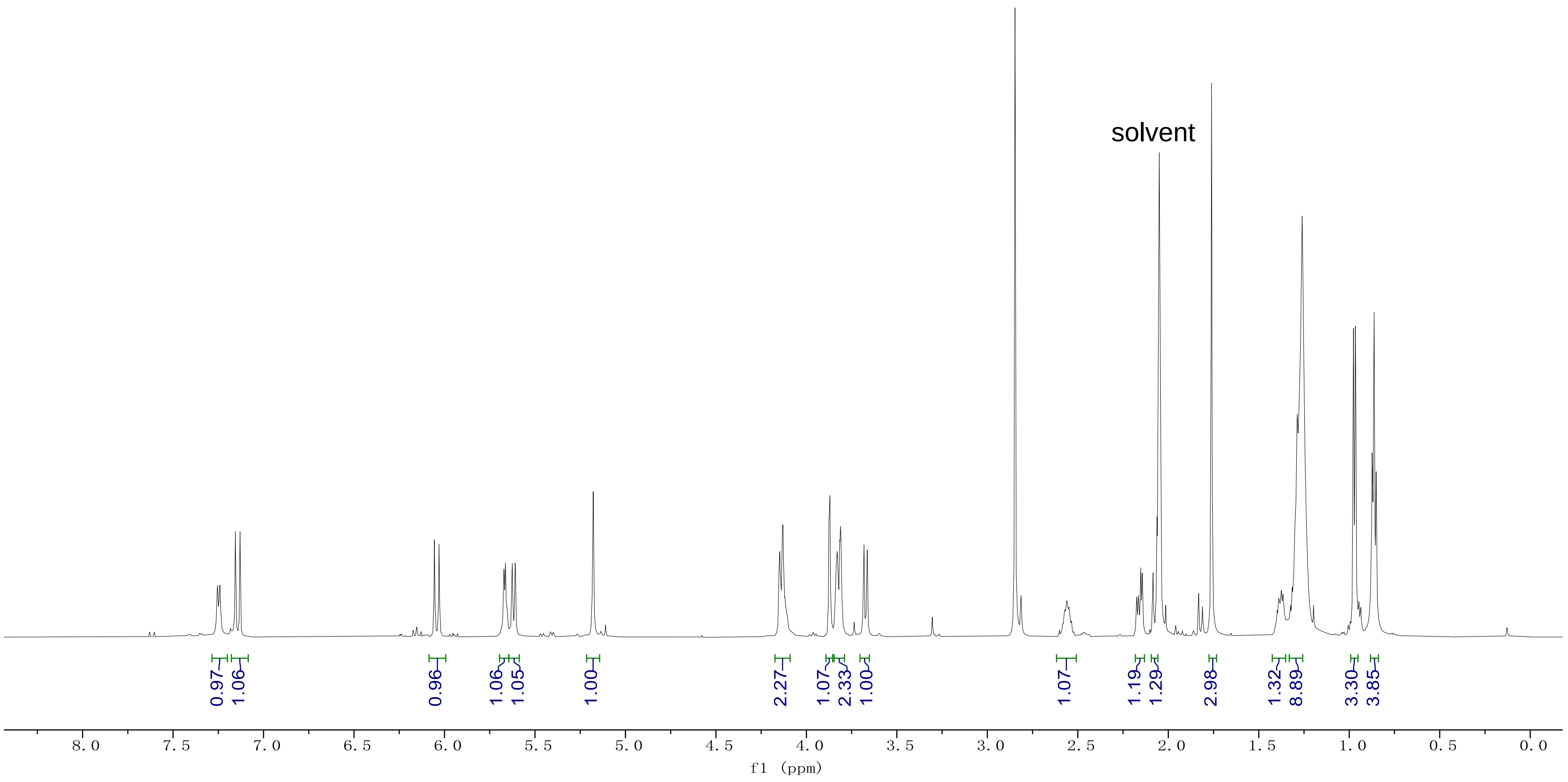

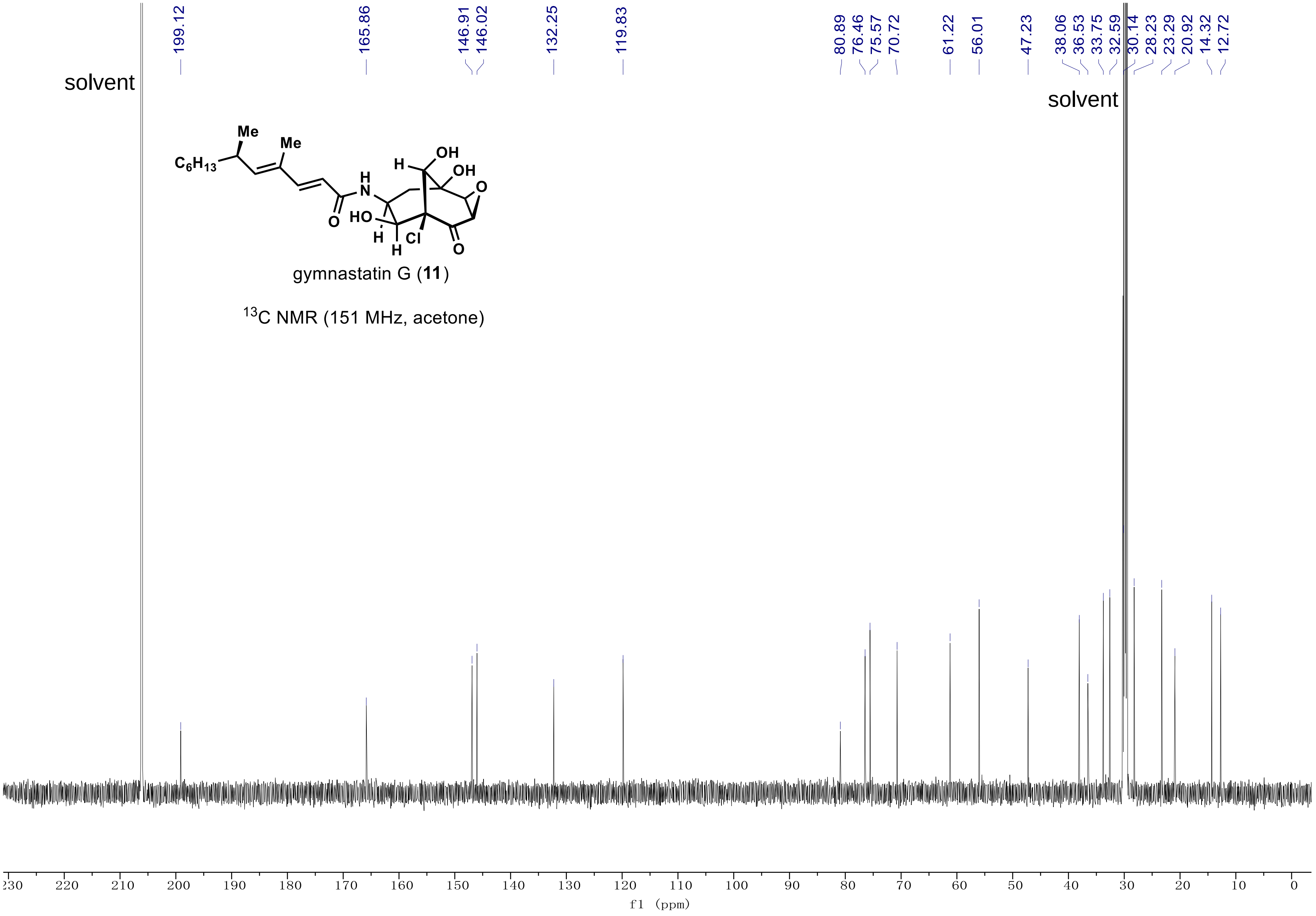
solvent
199.12
165.86
146.91
146.02
132.25
119.83
80.89
76.46
75.57
70.72
61.22
56.01
47.23
38.06
36.53
33.75
32.59
30.14
28.23
23.29
20.92
14.32
12.72
solvent
Me
Me
C_6H_{13}
H
N
O
HO
H
H
Cl
O
H
OH
OH
O
gymnastatin G (**11**)
^{13}C NMR (151 MHz, acetone)
230 220 210 200 190 180 170 160 150 140 130 120 110 100 90 80 70 60 50 40 30 20 10 0
f1 (ppm)

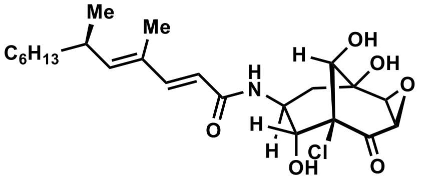

1-*epi*-gymnastatin G (**33**)

^{1}H NMR (600 MHz, acetone)

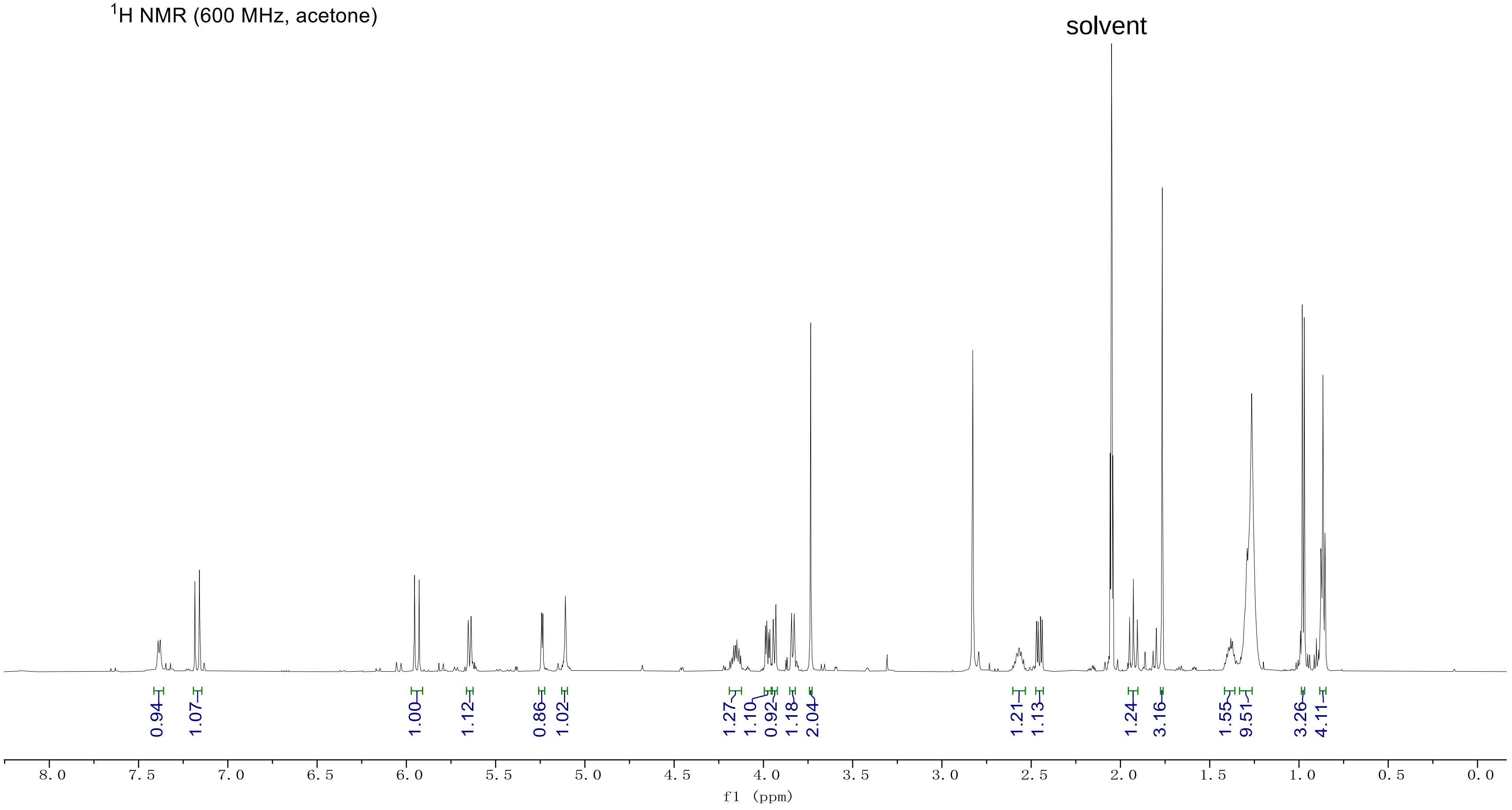

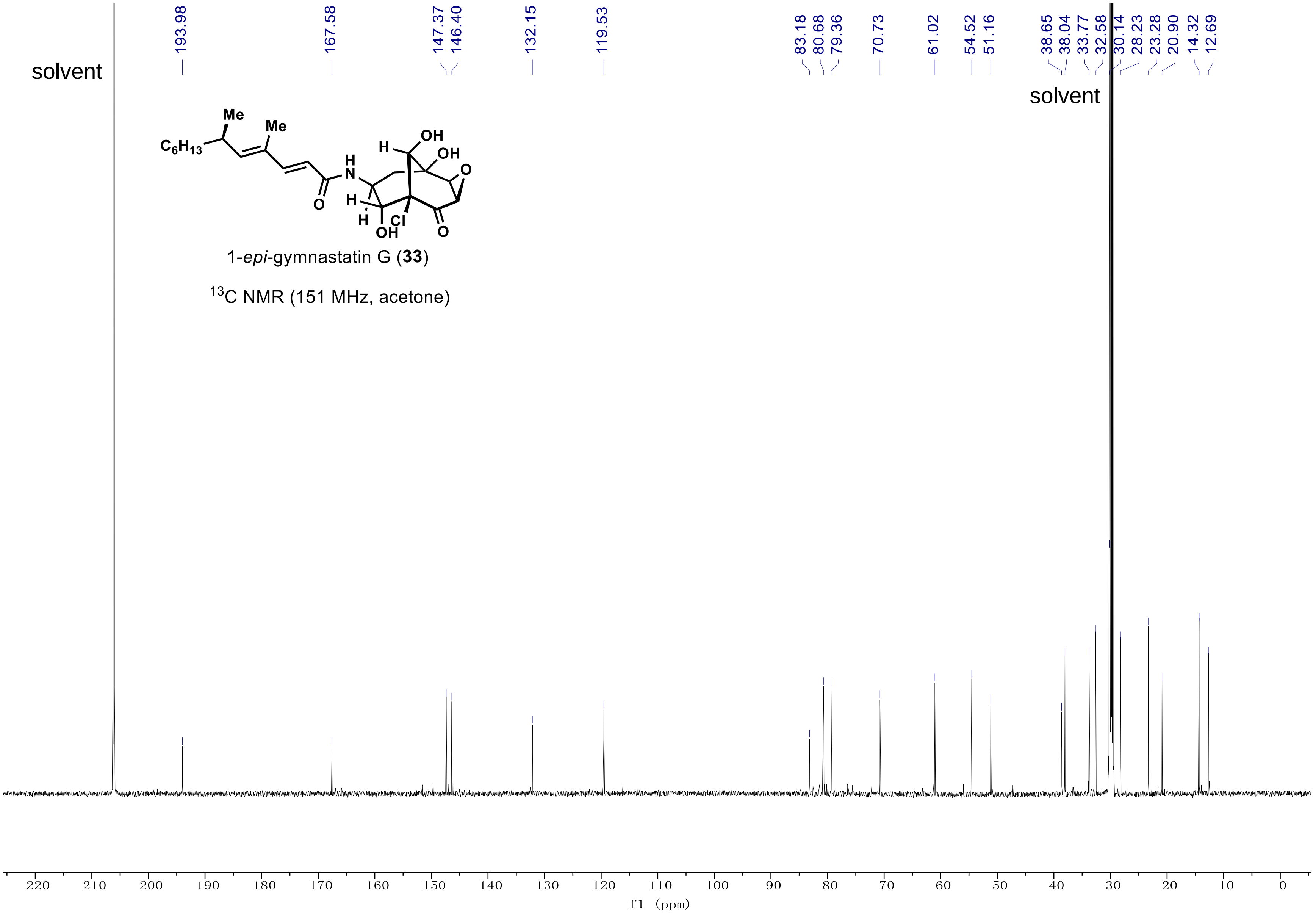
solvent
193.98
167.58
147.37
146.40
132.15
119.53
83.18
80.68
79.36
70.73
61.02
54.52
51.16
38.65
38.04
33.77
32.58
30.14
28.23
23.28
20.90
14.32
12.69
solvent
Me
Me
C6H13
H
N
H
OH
OH
O
O
H
H
Cl
OH
O
1-epi-gymnastatin G (33)
13C NMR (151 MHz, acetone)
220 210 200 190 180 170 160 150 140 130 120 110 100 90 80 70 60 50 40 30 20 10 0
f1 (ppm)

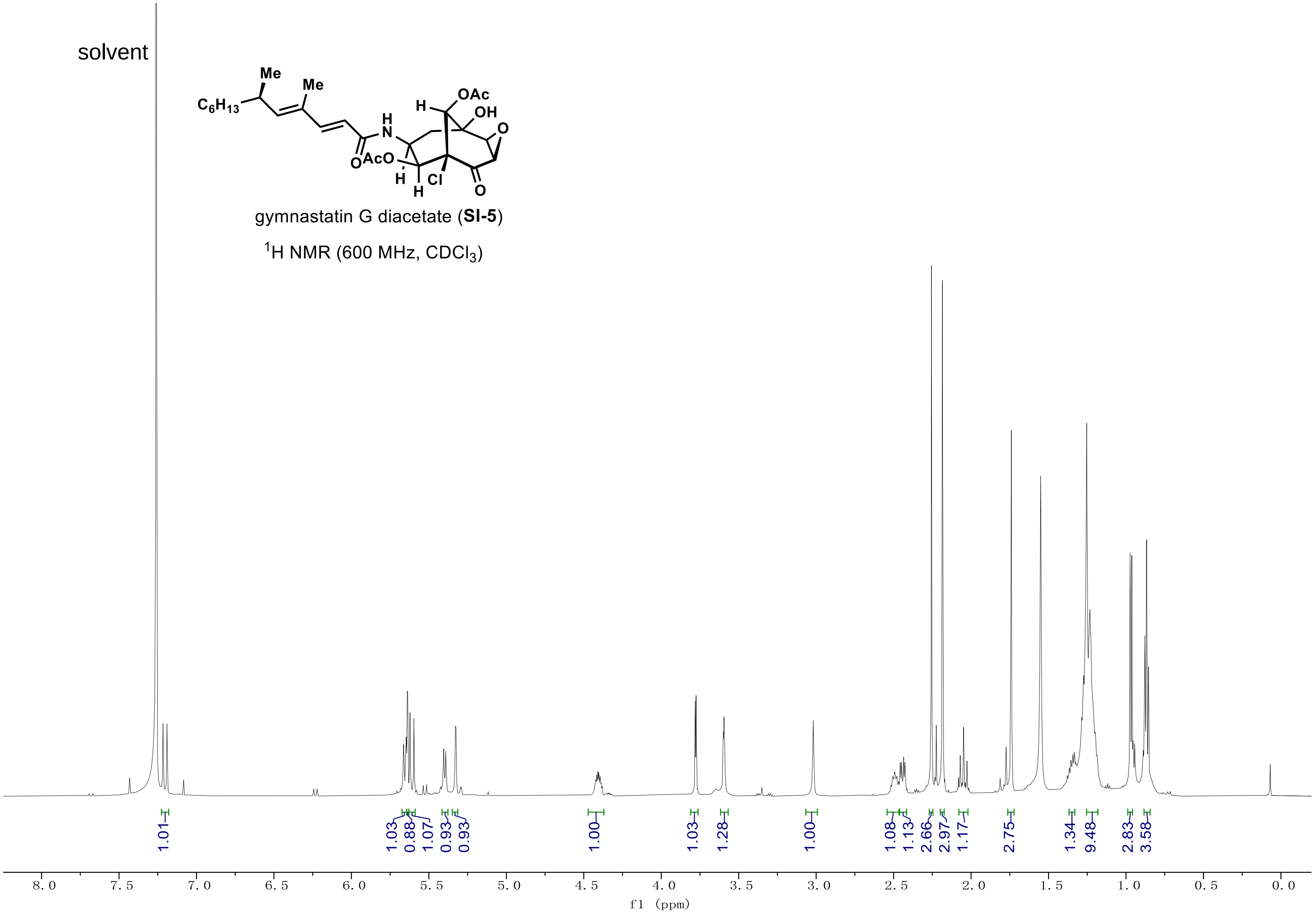
solvent
Me
Me
C6H13
H
OAc
OH
O
H
N
O
AcO
H
H
Cl
O
gymnastatin G diacetate (**SI-5**)
1H NMR (600 MHz, CDCl3)
1.01
1.03
0.88
1.07
0.93
0.93
1.00
1.03
1.28
1.00
1.08
1.13
2.66
2.97
1.17
2.75
1.34
9.48
2.83
3.58
8.0
7.5
7.0
6.5
6.0
5.5
5.0
4.5
4.0
3.5
3.0
2.5
2.0
1.5
1.0
0.5
0.0
f1 (ppm)

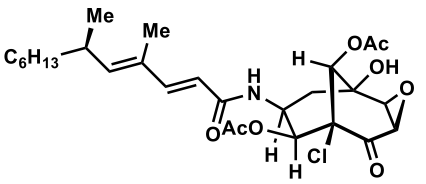

gymnastatin G diacetate (**SI-5**)

^{13}C NMR (151 MHz, $CDCl_3$)

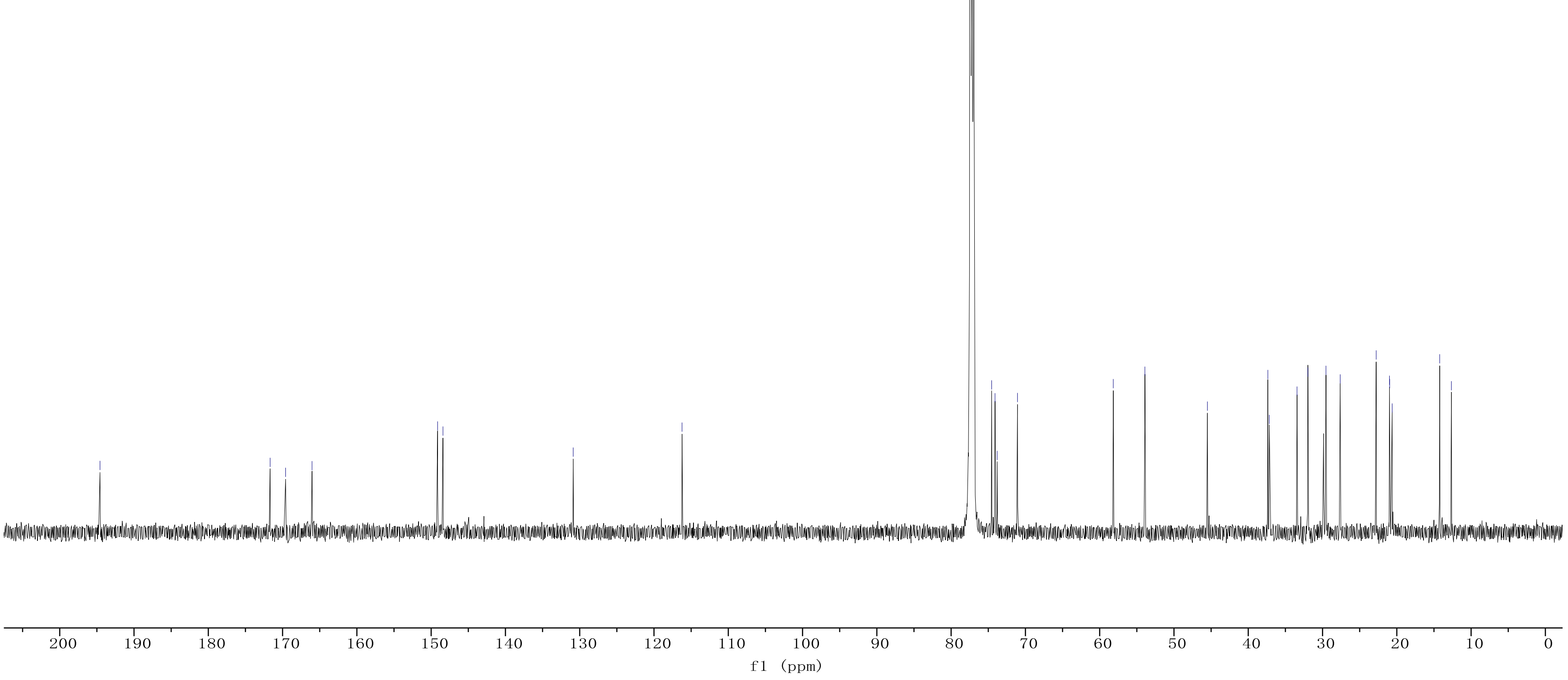

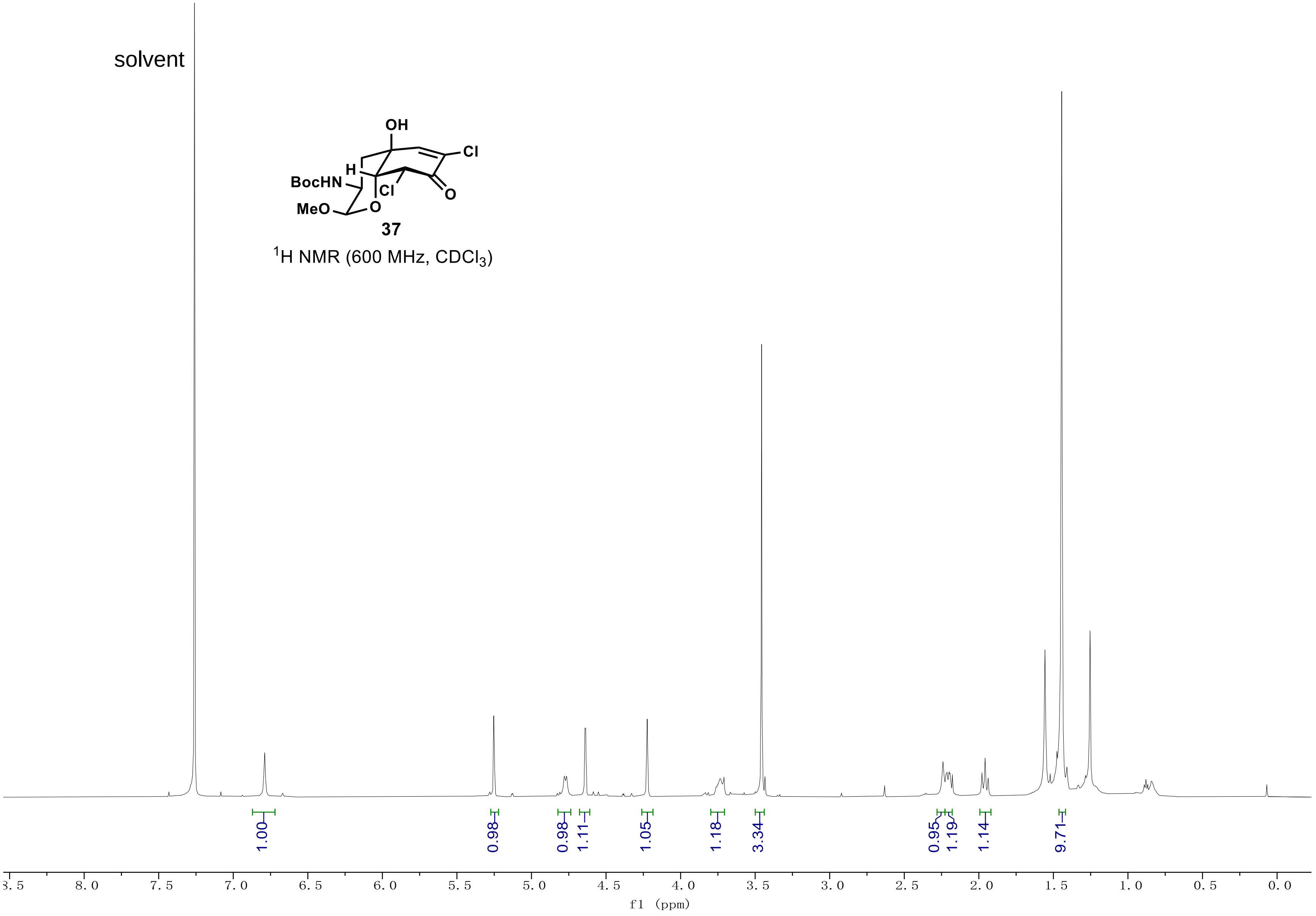
solvent
OH
Cl
H
BocHN
Cl
O
MeO
O
37
^{1}H NMR (600 MHz, $CDCl_3$)
1.00
0.98
0.98
1.11
1.05
1.18
3.34
0.95
1.19
1.14
9.71
8.5
8.0
7.5
7.0
6.5
6.0
5.5
5.0
4.5
4.0
3.5
3.0
2.5
2.0
1.5
1.0
0.5
0.0
f1 (ppm)

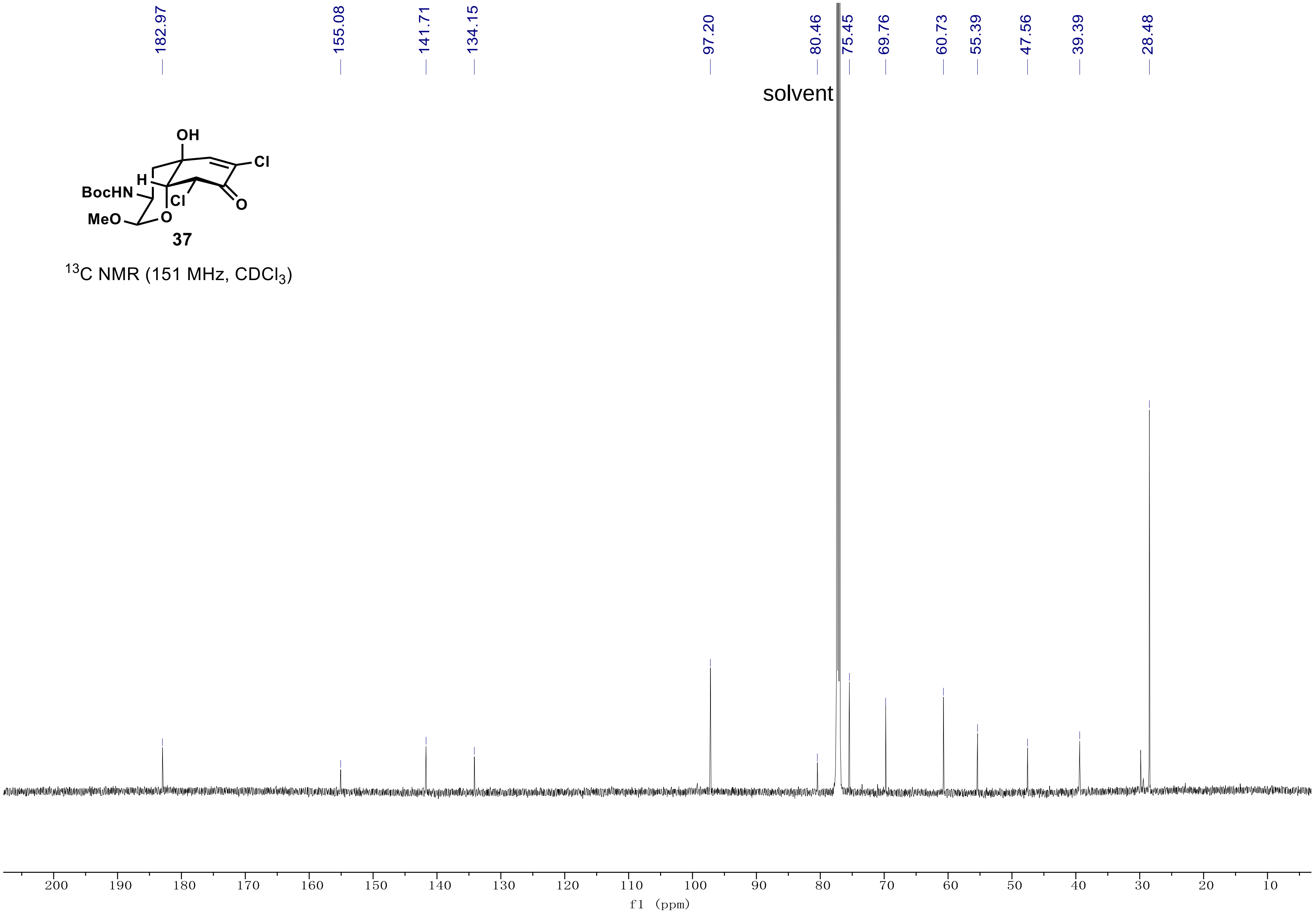
182.97
155.08
141.71
134.15
97.20
80.46
75.45
69.76
60.73
55.39
47.56
39.39
28.48
solvent
OH
Cl
H
BocHN
Cl
O
MeO
O
37
^{13}C NMR (151 MHz, CDCl$_3$)
200
190
180
170
160
150
140
130
120
110
100
90
80
70
60
50
40
30
20
10
f1 (ppm)

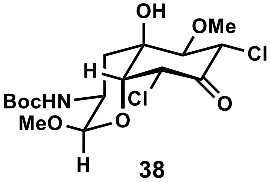

38

^{1}H NMR (600 MHz, $CDCl_3$)

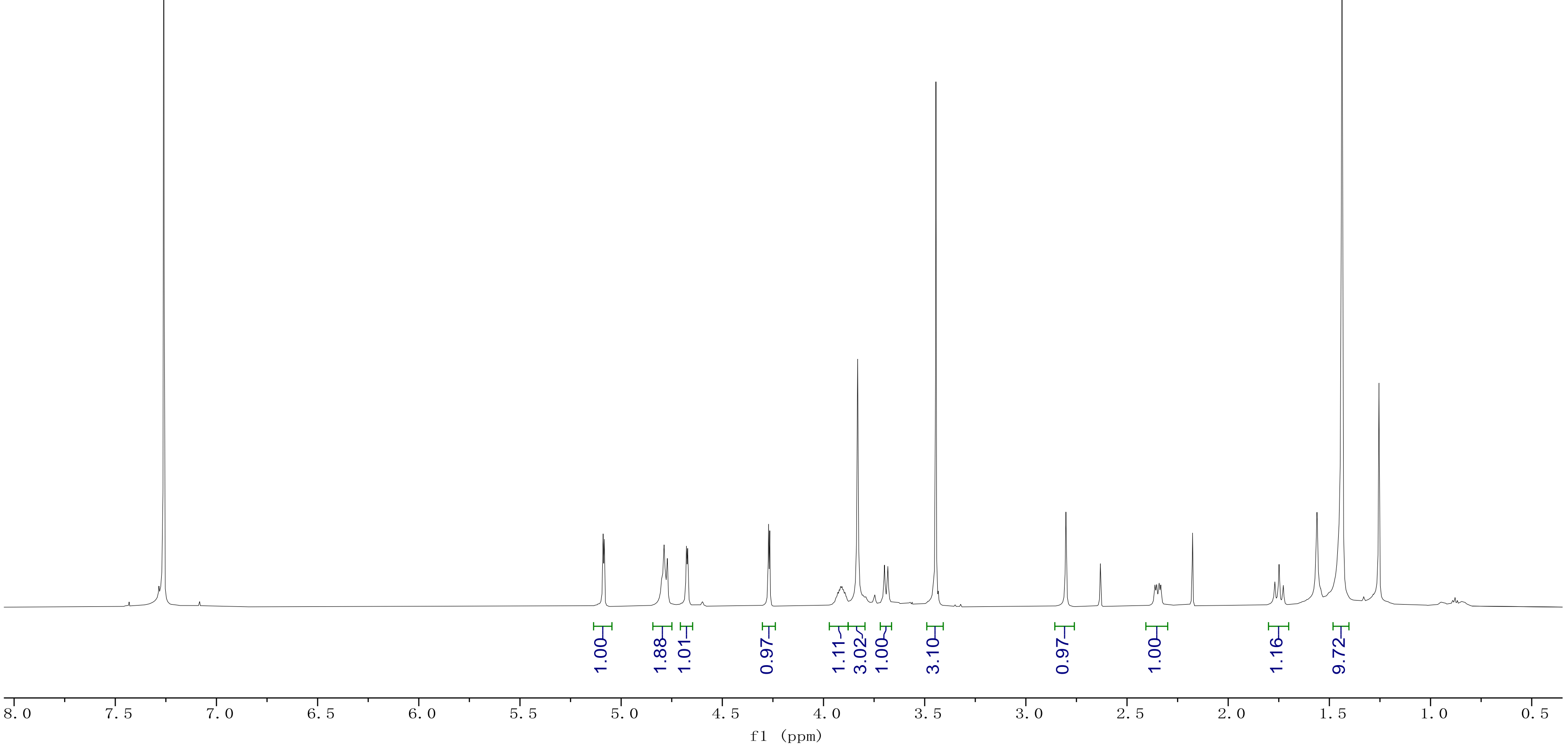

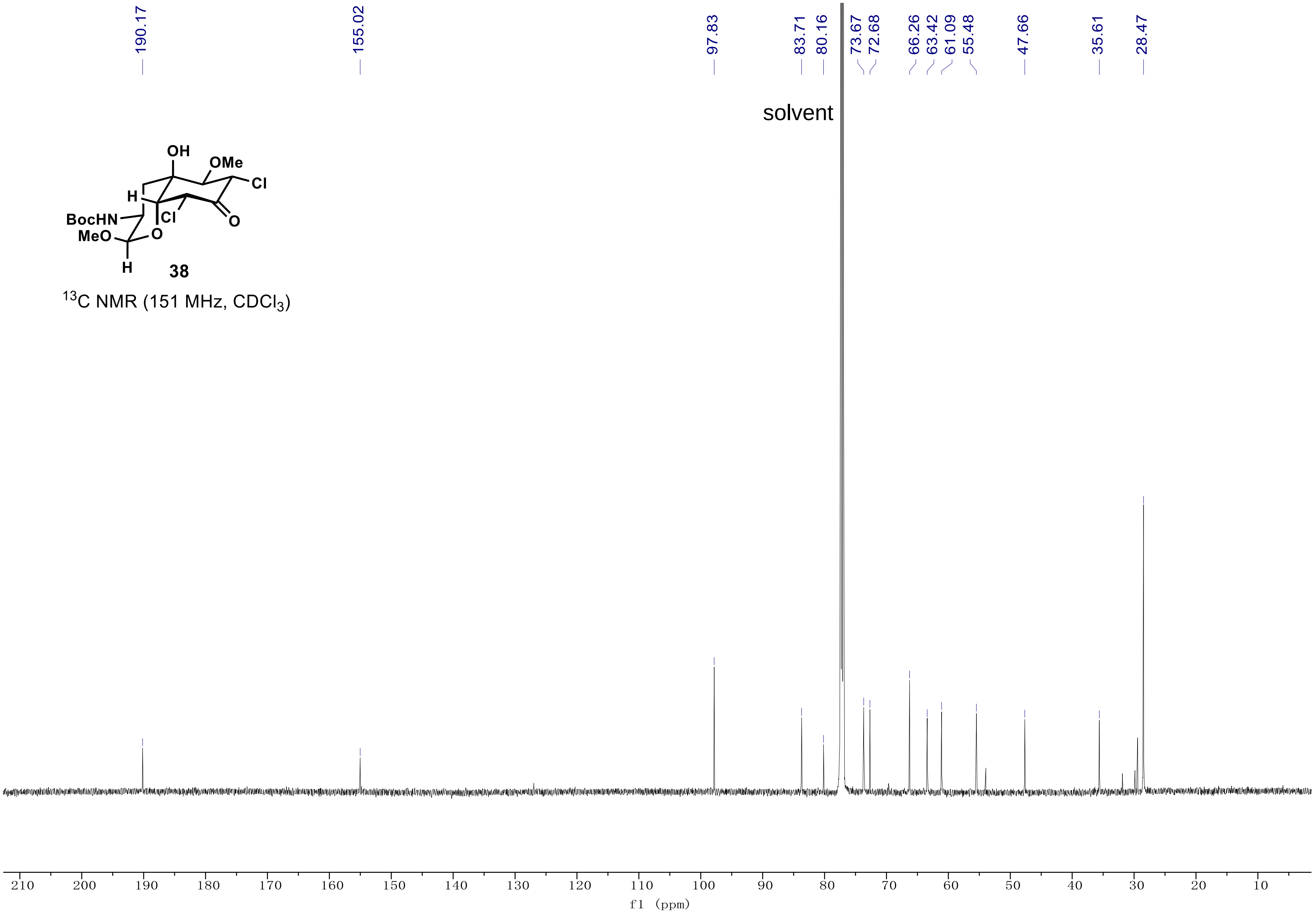
190.17
155.02
97.83
83.71
80.16
73.67
72.68
66.26
63.42
61.09
55.48
47.66
35.61
28.47
solvent
OH
OMe
Cl
H
BocHN
Cl
O
MeO
O
H
38
^{13}C NMR (151 MHz, $CDCl_3$)
210
200
190
180
170
160
150
140
130
120
110
100
90
80
70
60
50
40
30
20
10
f1 (ppm)

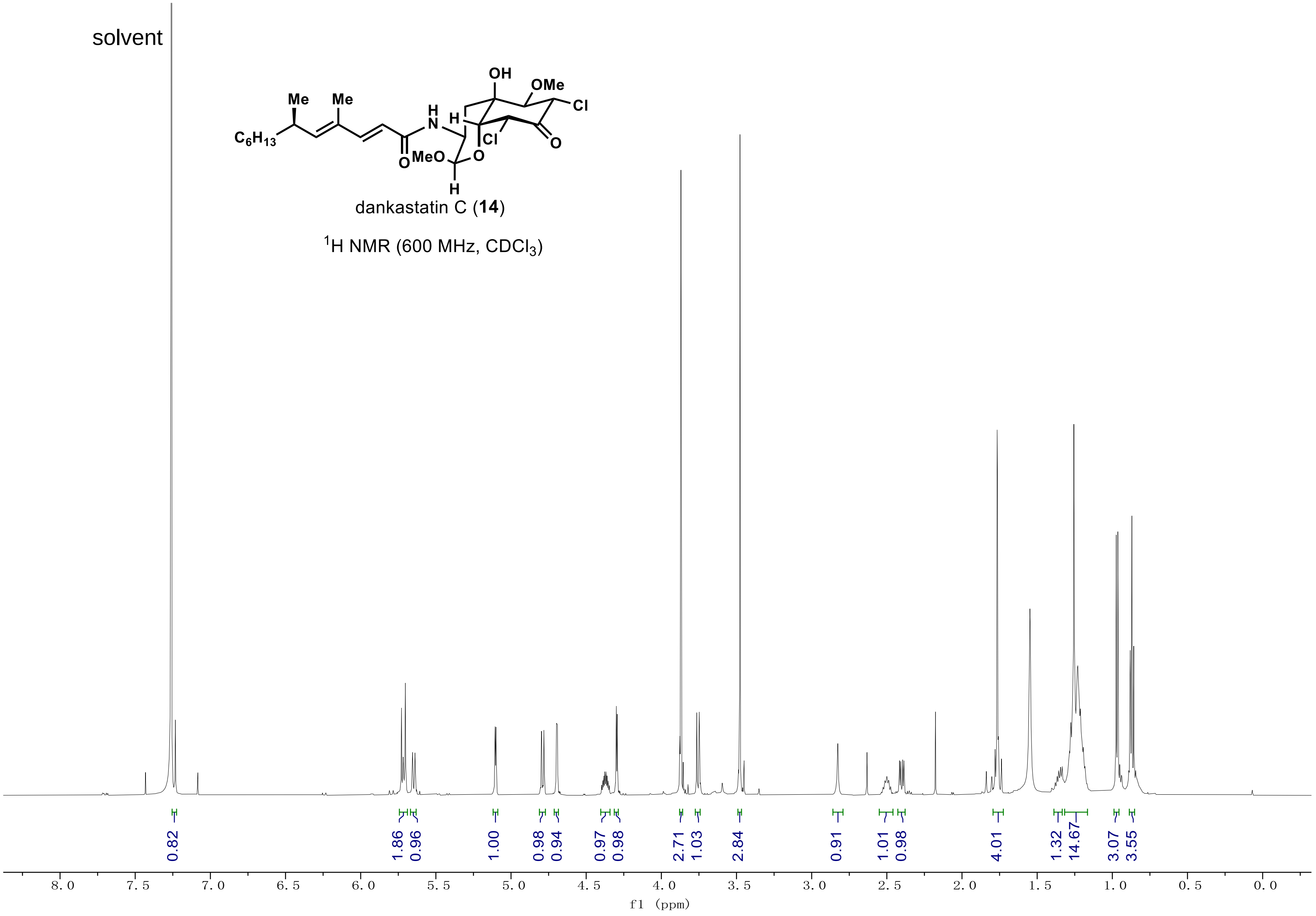

solvent
OH
OMe
Cl
Me
Me
H
N
H
C6H13
Cl
O
O
MeO
O
H
dankastatin C (14)
1H NMR (600 MHz, CDCl3)
0.82
1.86
0.96
1.00
0.98
0.94
0.97
0.98
2.71
1.03
2.84
0.91
1.01
0.98
4.01
1.32
14.67
3.07
3.55
8.0
7.5
7.0
6.5
6.0
5.5
5.0
4.5
4.0
3.5
3.0
2.5
2.0
1.5
1.0
0.5
0.0
f1 (ppm)

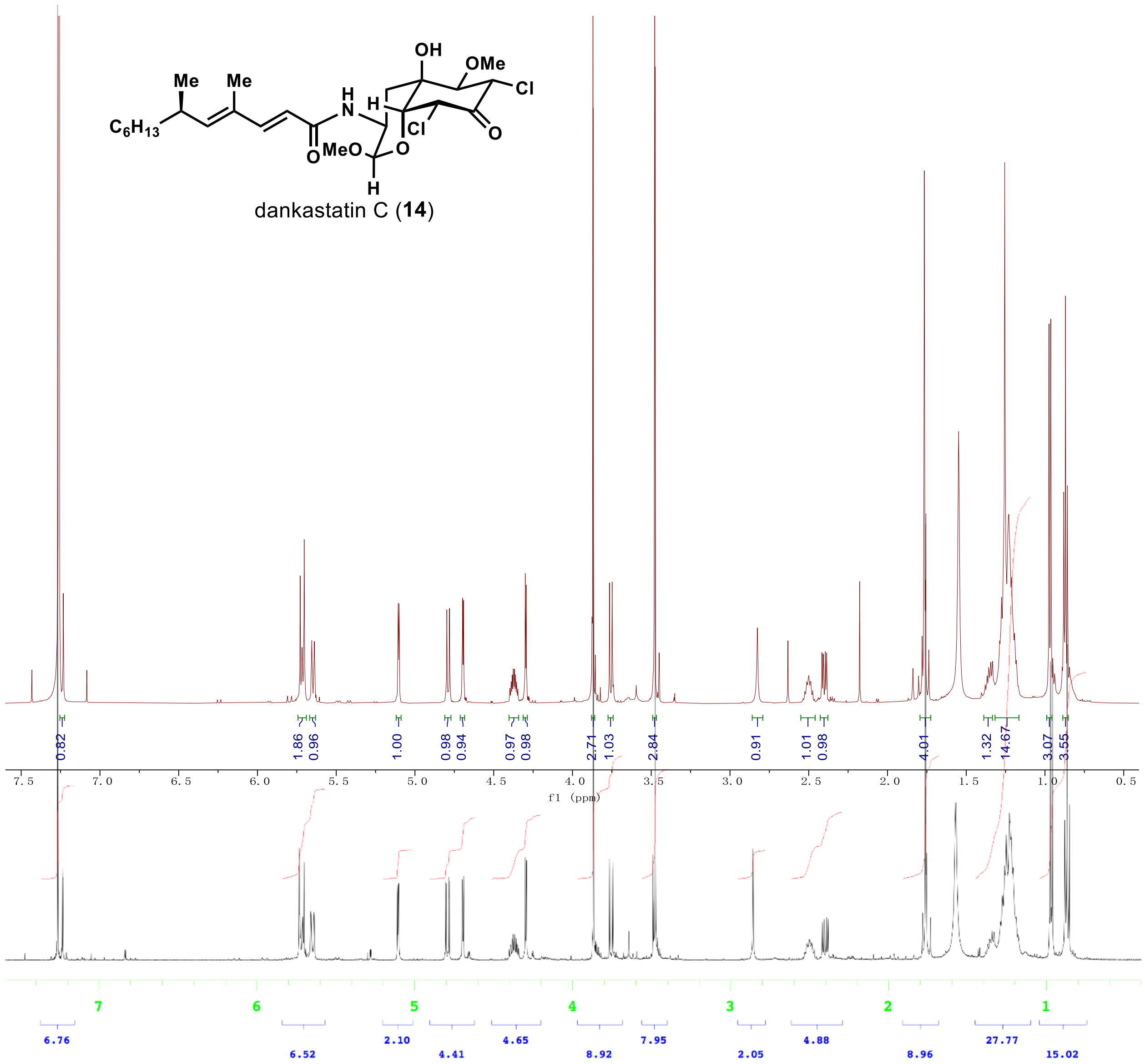

Top: synthetic dankastatin C

Bottom: natural dankastatin C

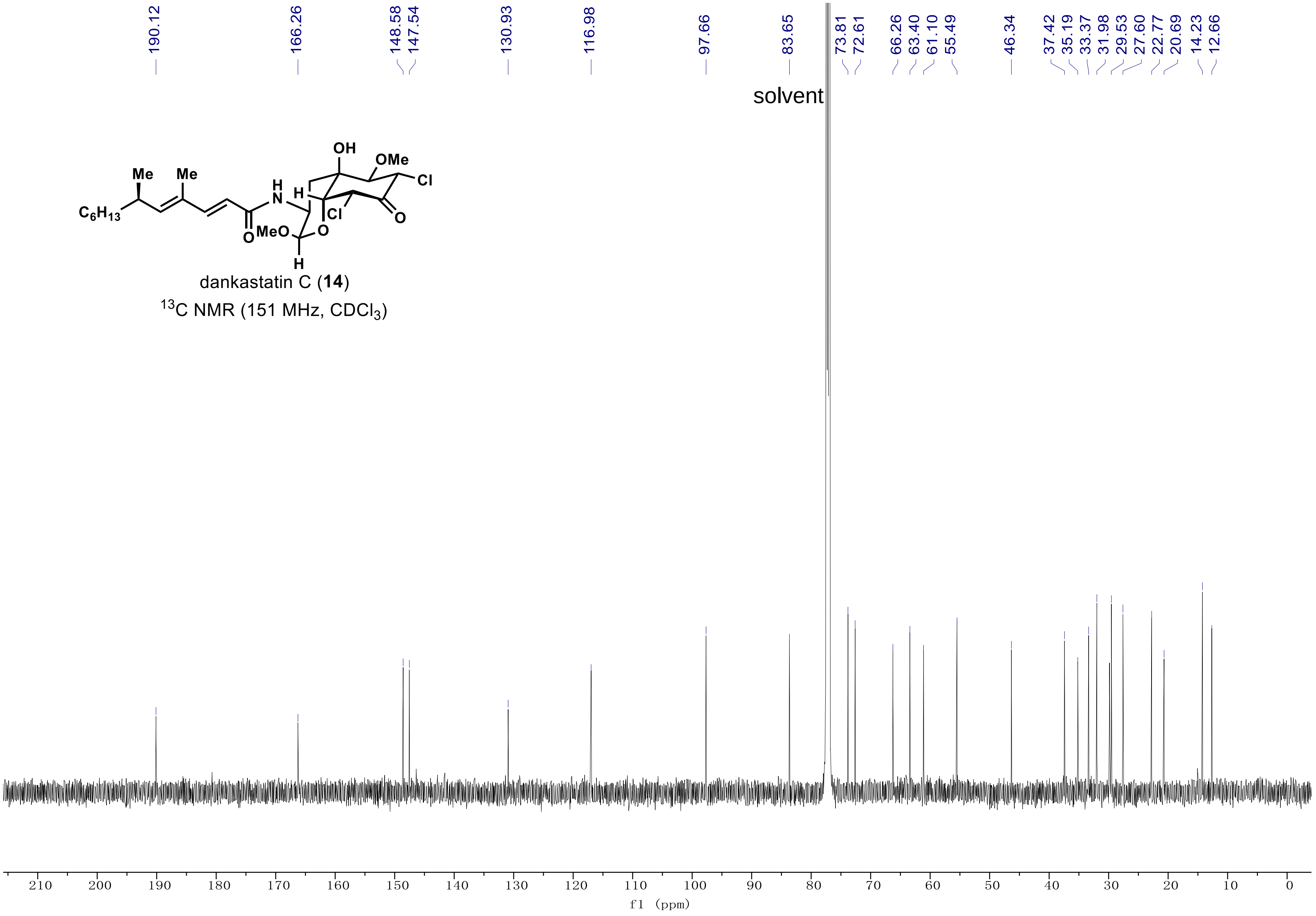

dankastatin C (**14**)

^{13}C NMR (151 MHz, $CDCl_3$)

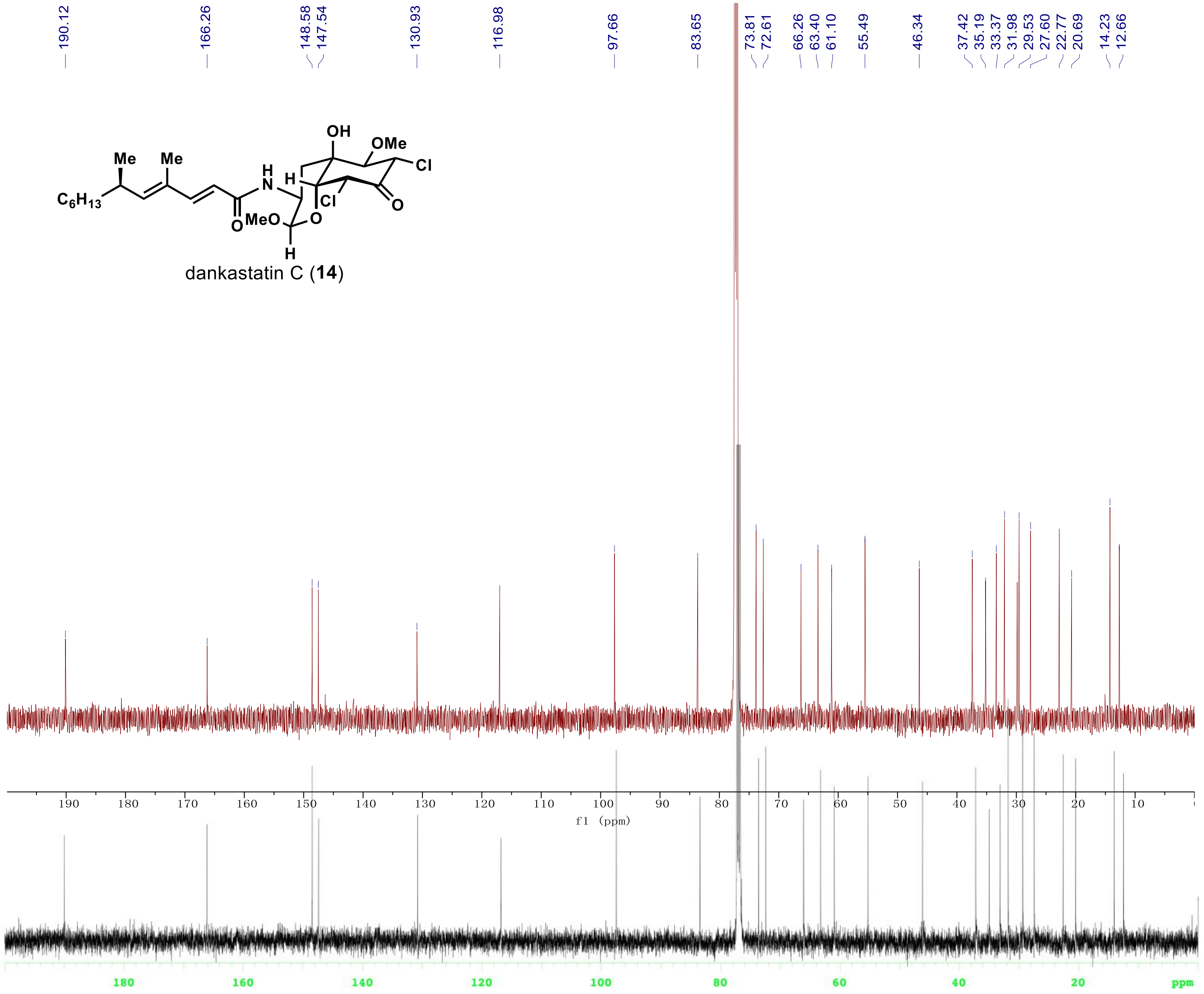

Top: synthetic dankastatin C

Bottom: natural dankastatin C

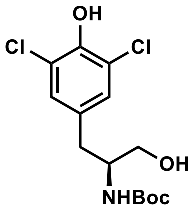

40

^{1}H NMR (600 MHz, $CDCl_3$)

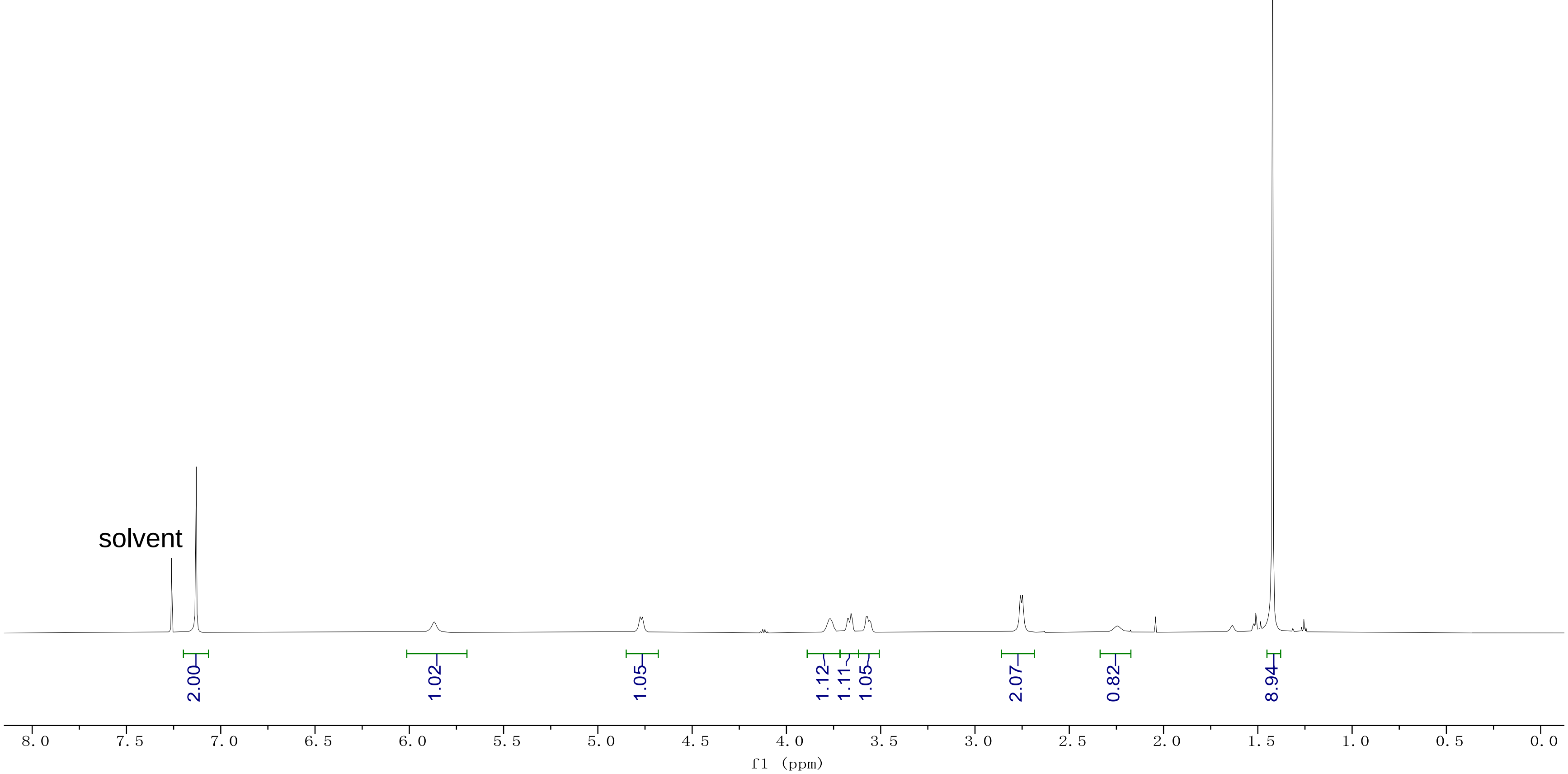

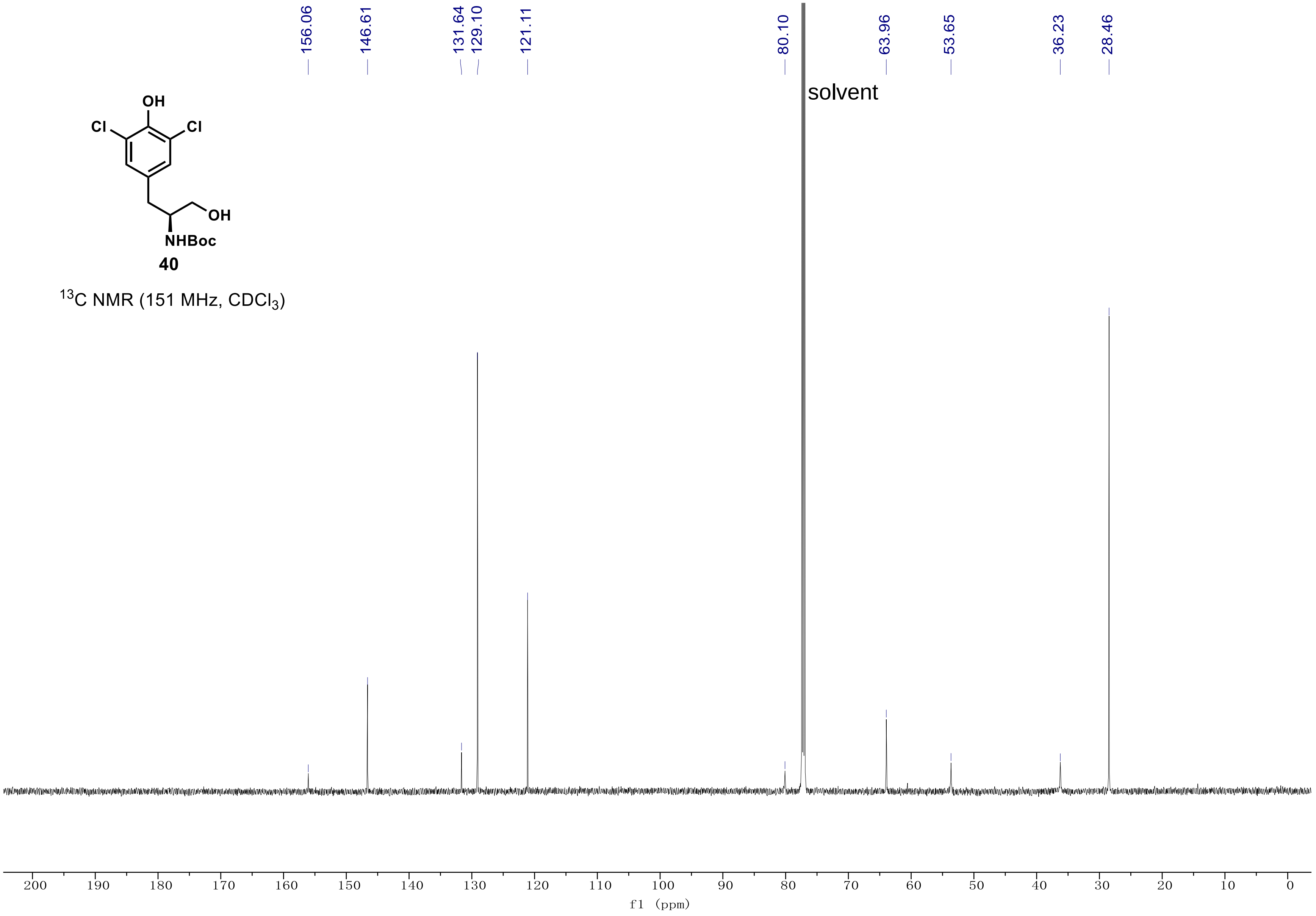

40

^{13}C NMR (151 MHz, $CDCl_3$)

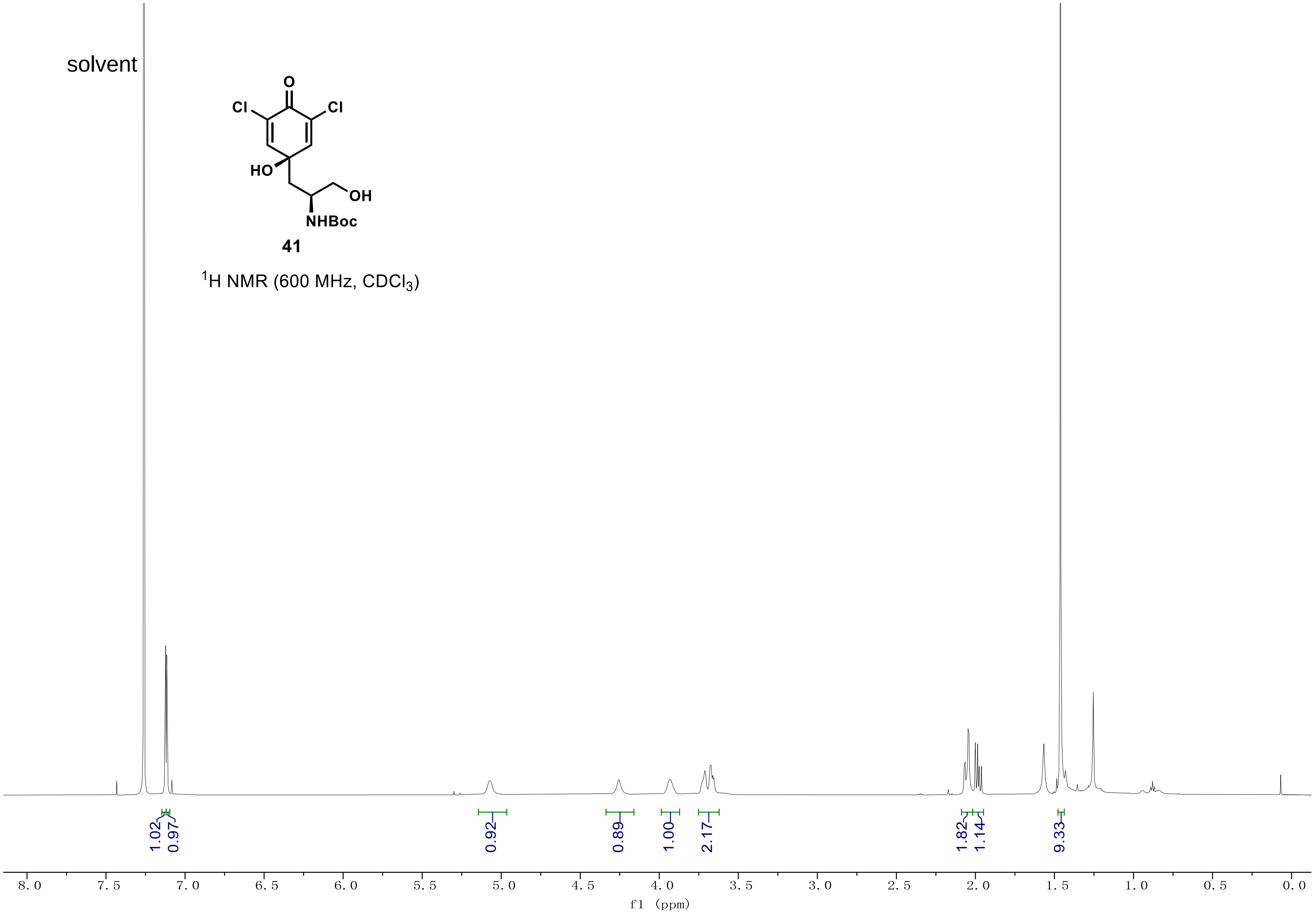
solvent
O
Cl
Cl
HO
OH
NHBoc
41
^{1}H NMR (600 MHz, $CDCl_3$)
1.02
0.97
0.92
0.89
1.00
2.17
1.82
1.14
9.33
8.0
7.5
7.0
6.5
6.0
5.5
5.0
4.5
4.0
3.5
3.0
2.5
2.0
1.5
1.0
0.5
0.0
f1 (ppm)

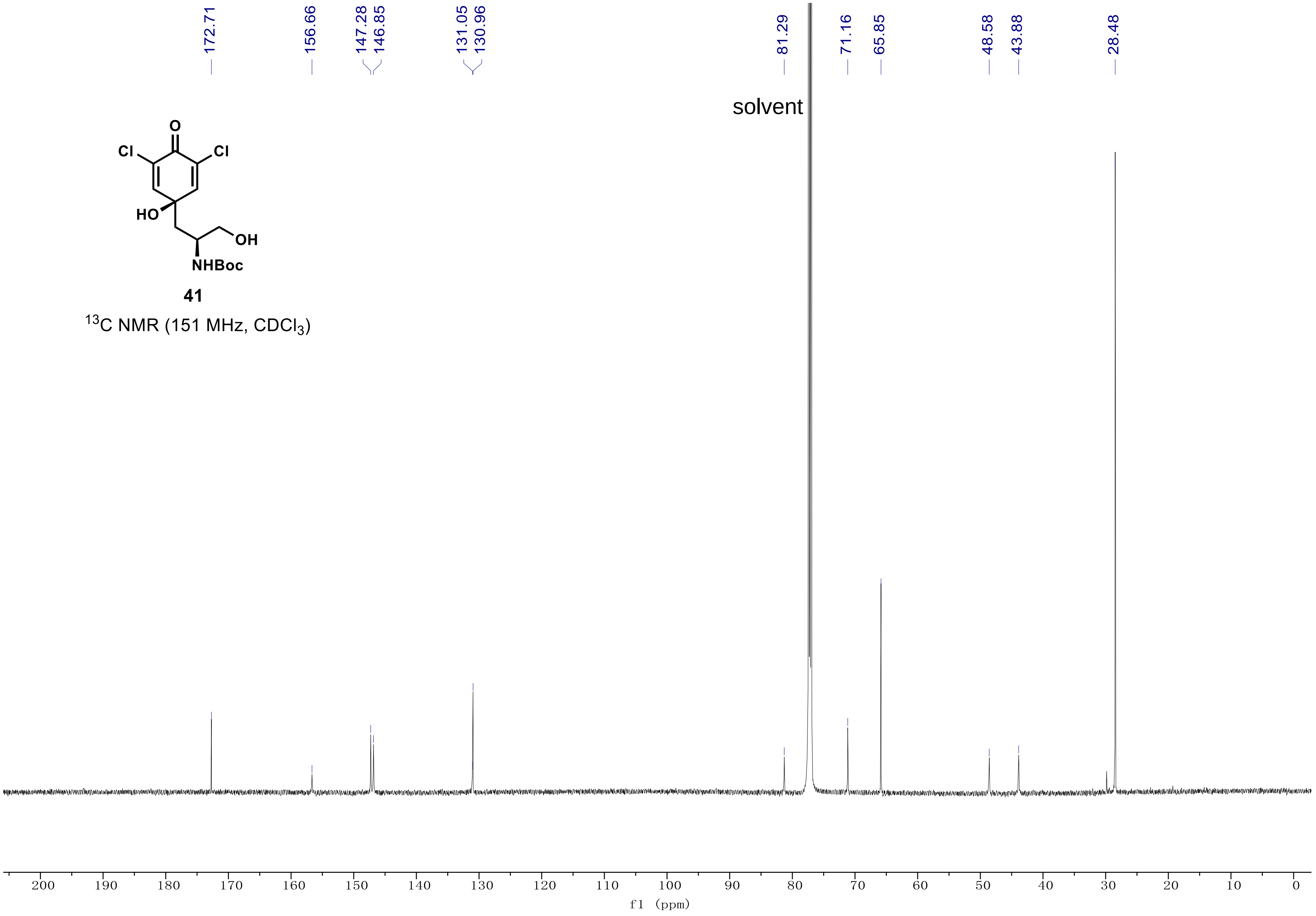
172.71
156.66
147.28
146.85
131.05
130.96
81.29
71.16
65.85
48.58
43.88
28.48
solvent
O
Cl
Cl
HO
OH
NHBoc
41
^{13}C NMR (151 MHz, $CDCl_3$)
200
190
180
170
160
150
140
130
120
110
100
90
80
70
60
50
40
30
20
10
0
f1 (ppm)

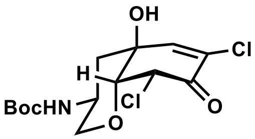

42

^{1}H NMR (700 MHz, $CDCl_3$)

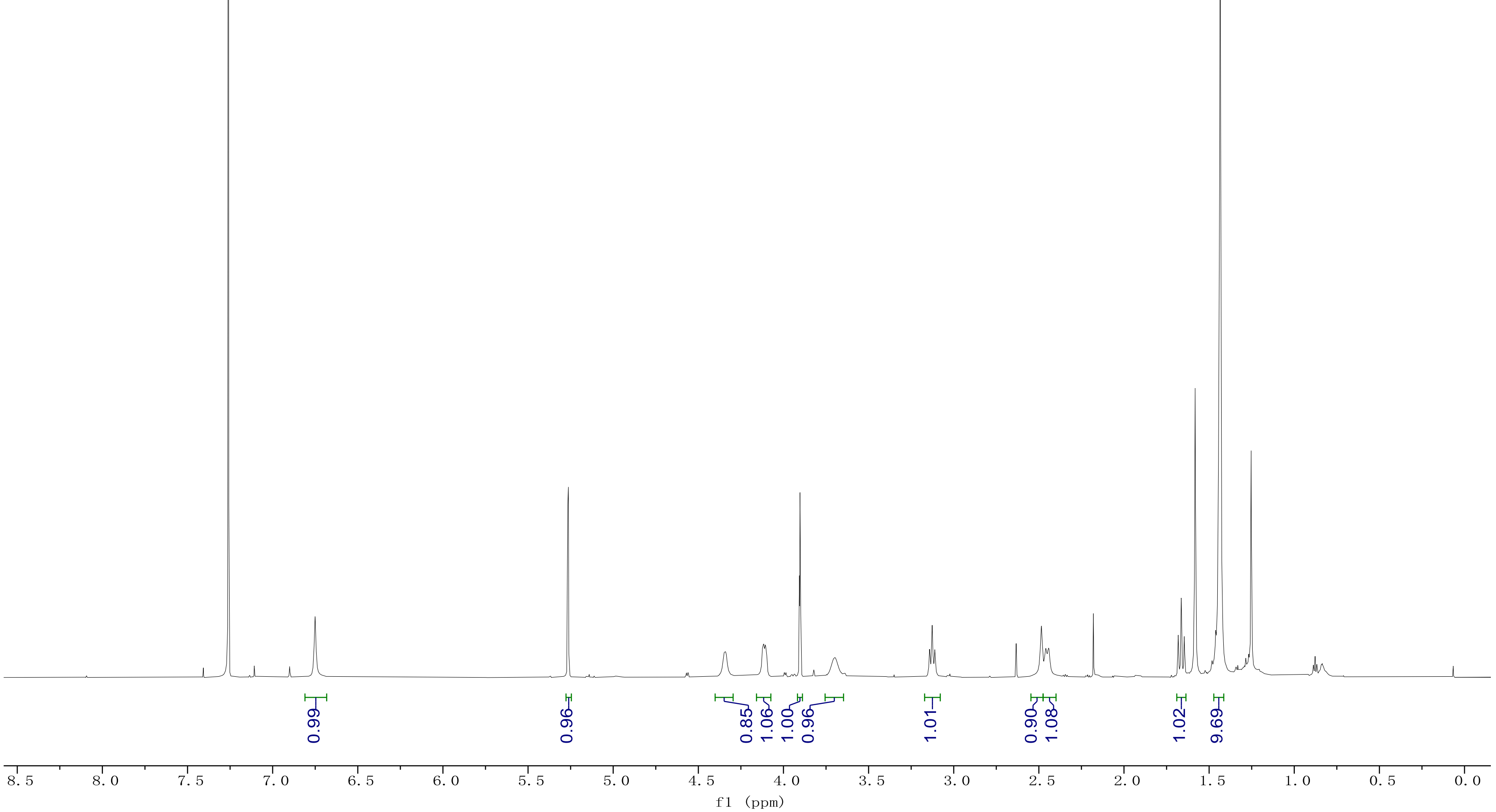

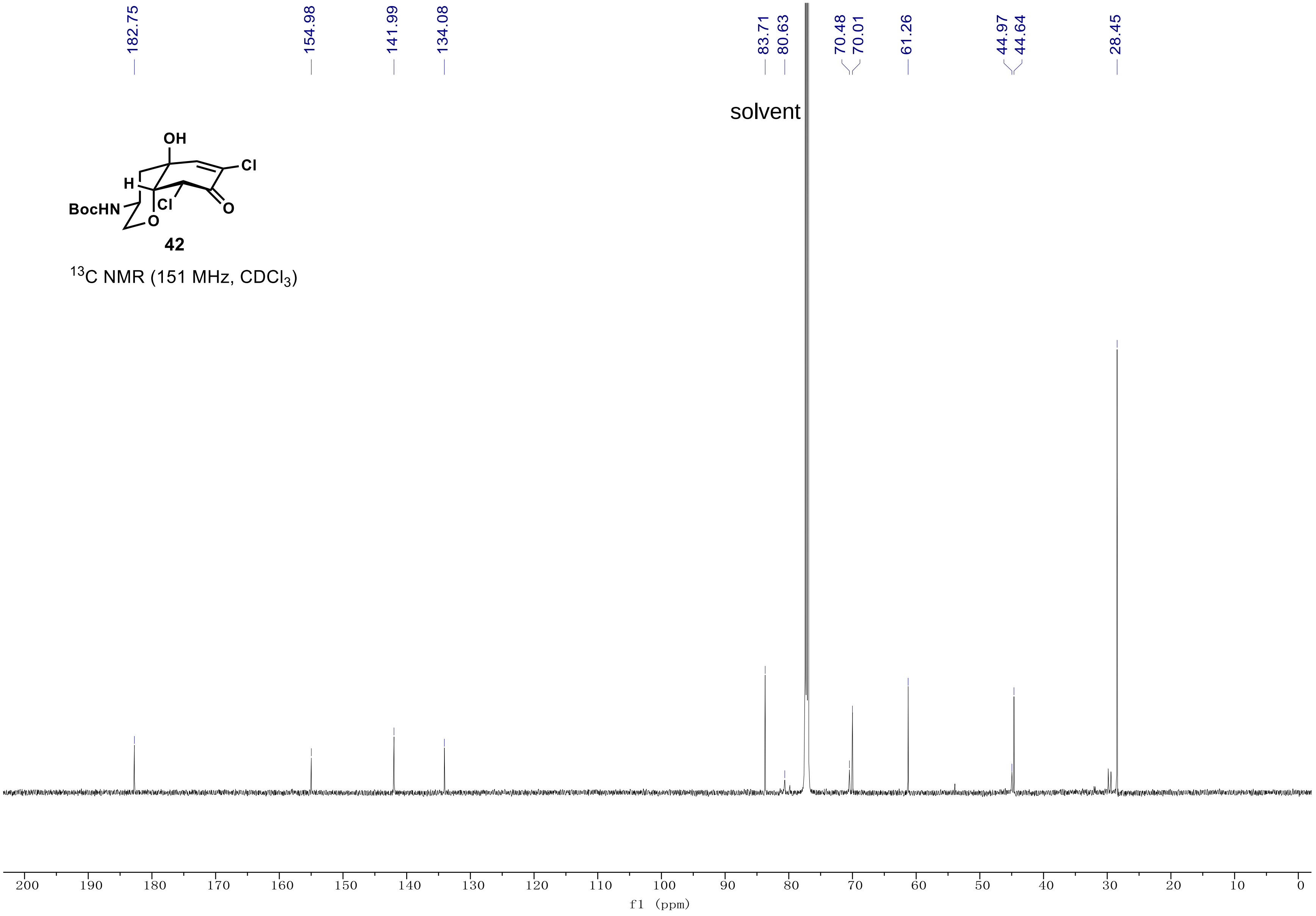

42

^{13}C NMR (151 MHz, $CDCl_3$)

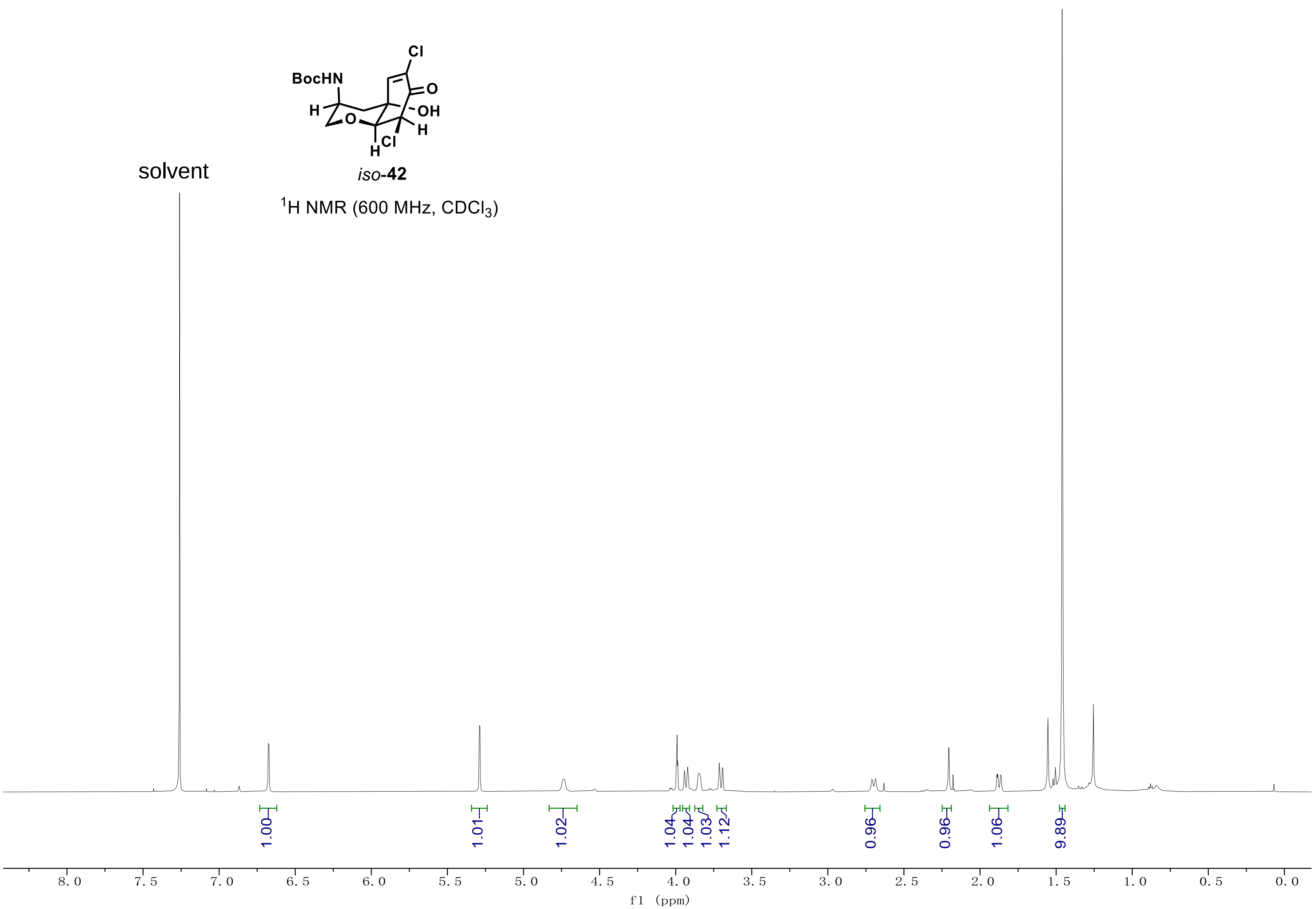

solvent
Cl
BocHN
O
H
OH
Cl
iso-42
1H NMR (600 MHz, CDCl3)
1.00
1.01
1.02
1.04
1.04
1.03
1.12
0.96
0.96
1.06
9.89
8.0 7.5 7.0 6.5 6.0 5.5 5.0 4.5 4.0 3.5 3.0 2.5 2.0 1.5 1.0 0.5 0.0
f1 (ppm)

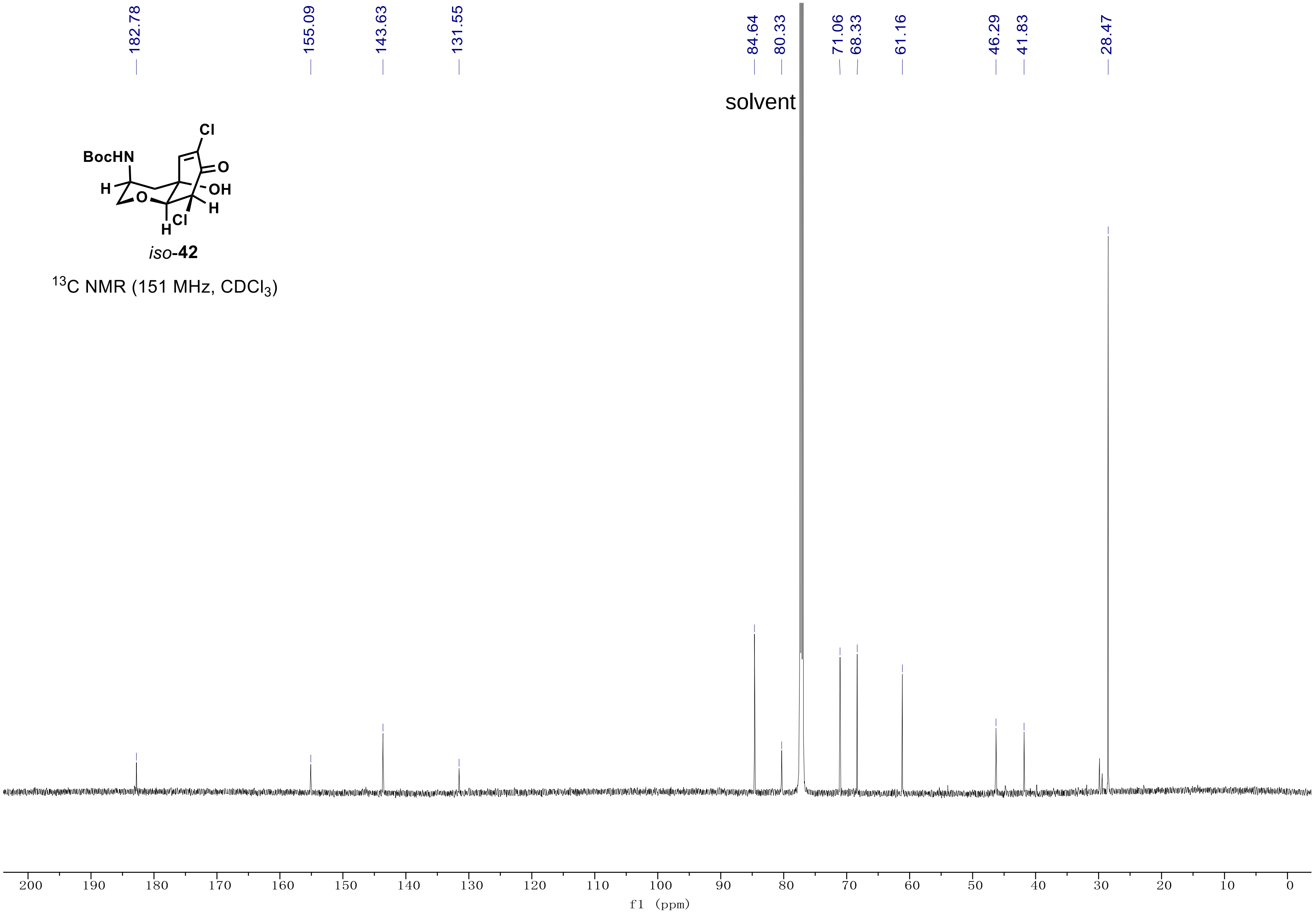

182.78
155.09
143.63
131.55
84.64
80.33
71.06
68.33
61.16
46.29
41.83
28.47
solvent
BocHN
Cl
O
H
OH
H
O
Cl
H
iso-42
13C NMR (151 MHz, CDCl3)
f1 (ppm)

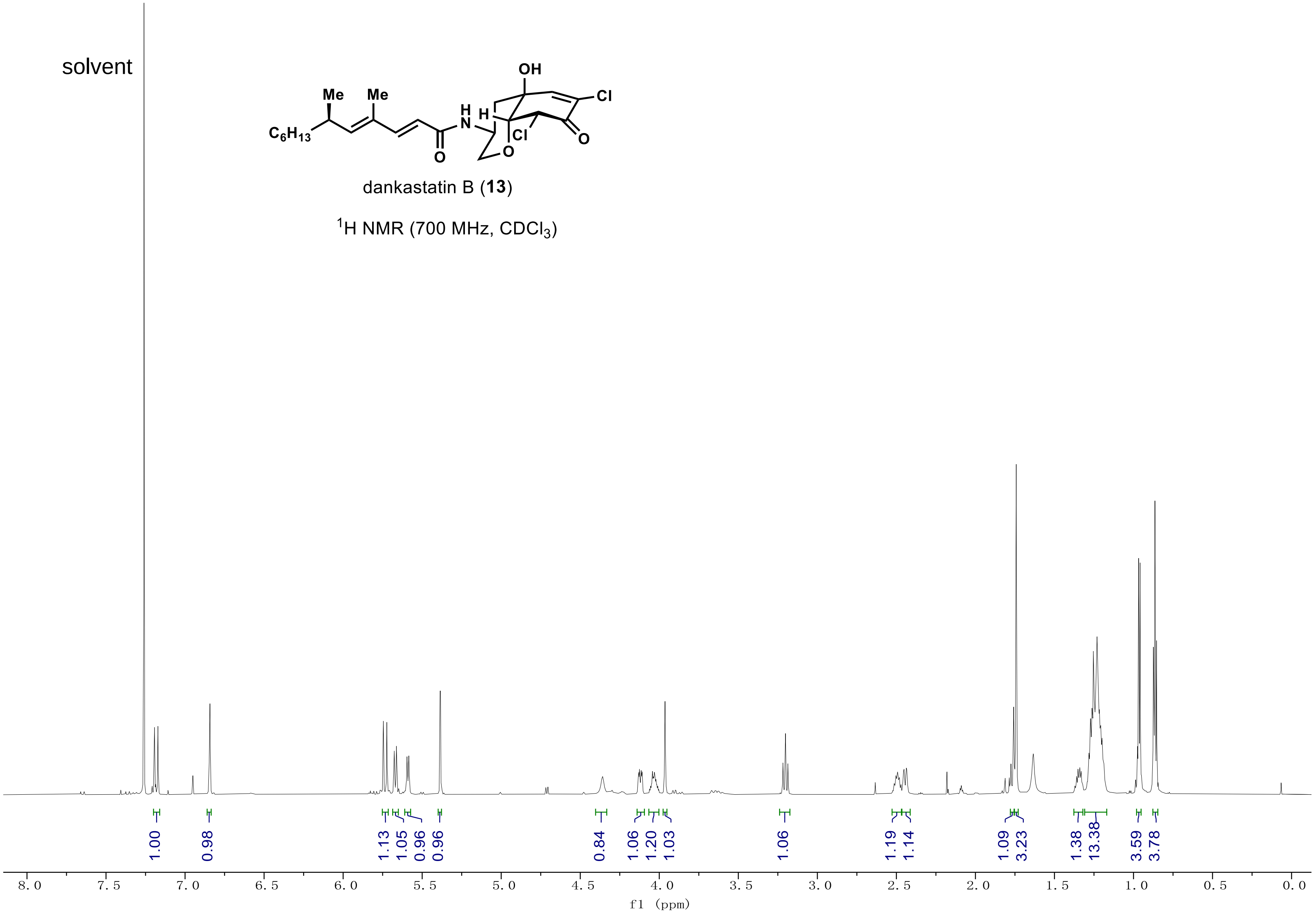

dankastatin B (**13**)

^{1}H NMR (700 MHz, $CDCl_3$)

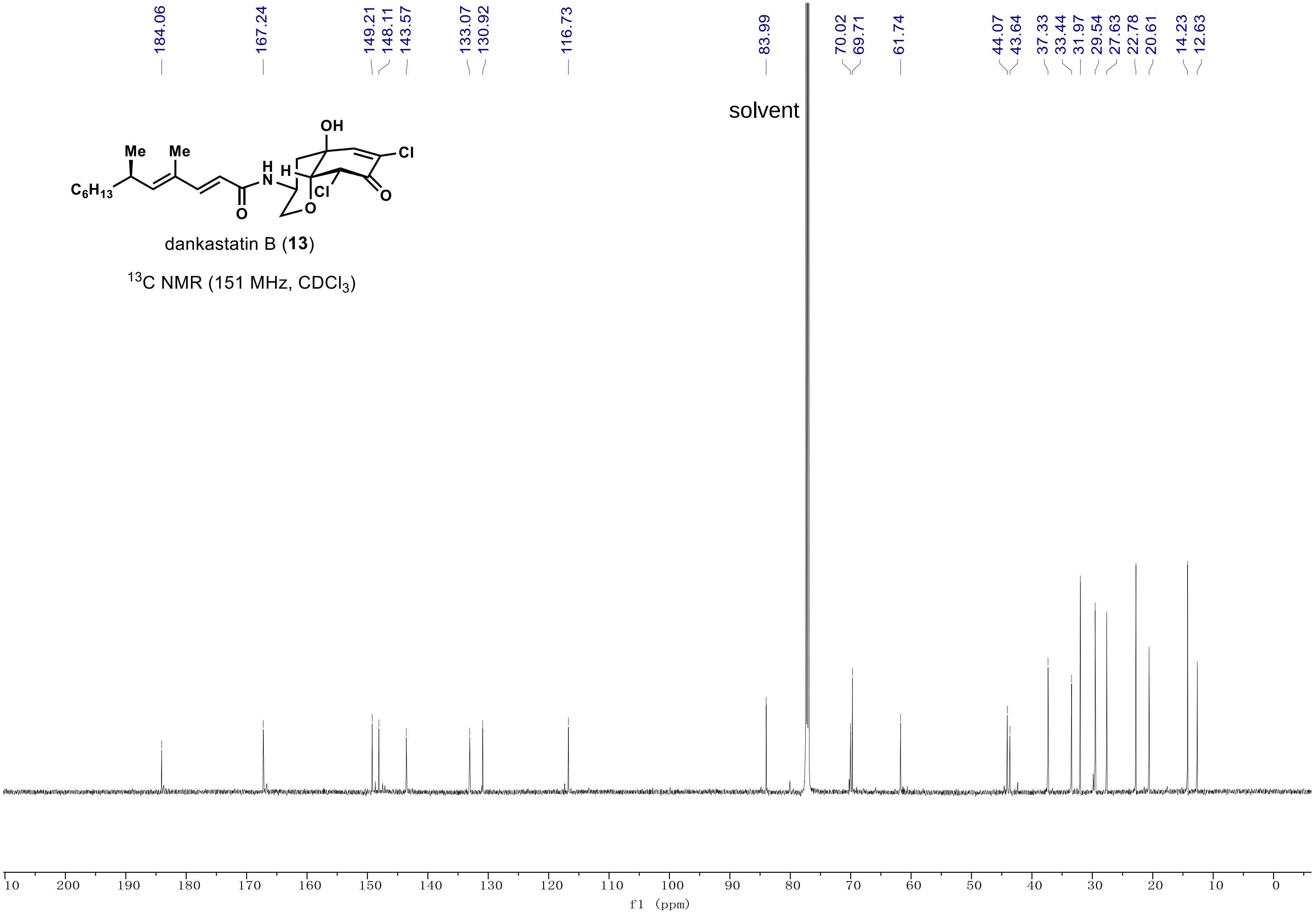

dankastatin B (**13**)

^{13}C NMR (151 MHz, $CDCl_3$)

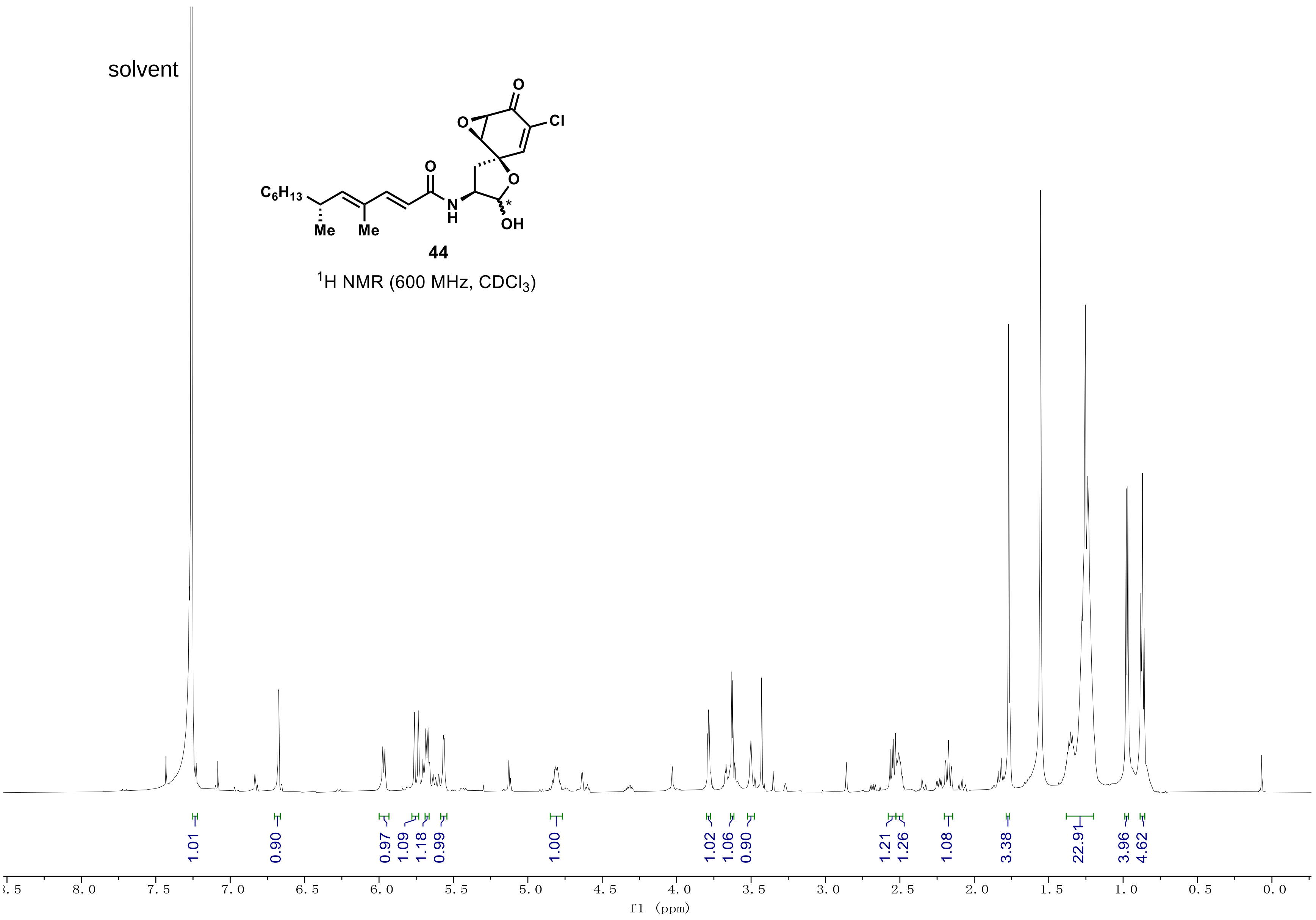
solvent
O
O
Cl
O
C6H13
N
H
O
*
OH
Me
Me
44
1H NMR (600 MHz, CDCl3)
1.01
0.90
0.97
1.09
1.18
0.99
1.00
1.02
1.06
0.90
1.21
1.26
1.08
3.38
22.91
3.96
4.62
8.0
7.5
7.0
6.5
6.0
5.5
5.0
4.5
4.0
3.5
3.0
2.5
2.0
1.5
1.0
0.5
0.0
f1 (ppm)

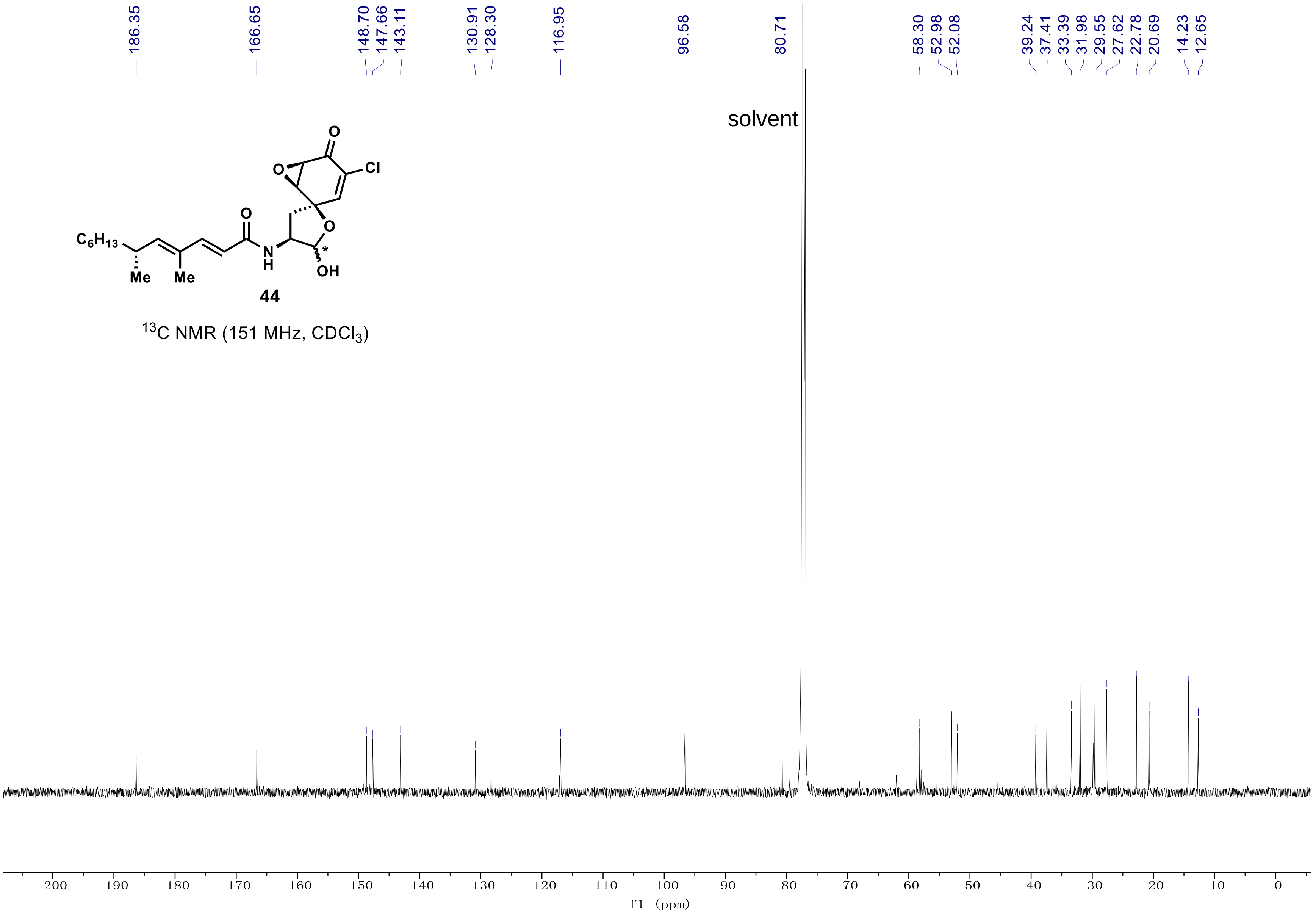

^{13}C NMR (151 MHz, $CDCl_3$)

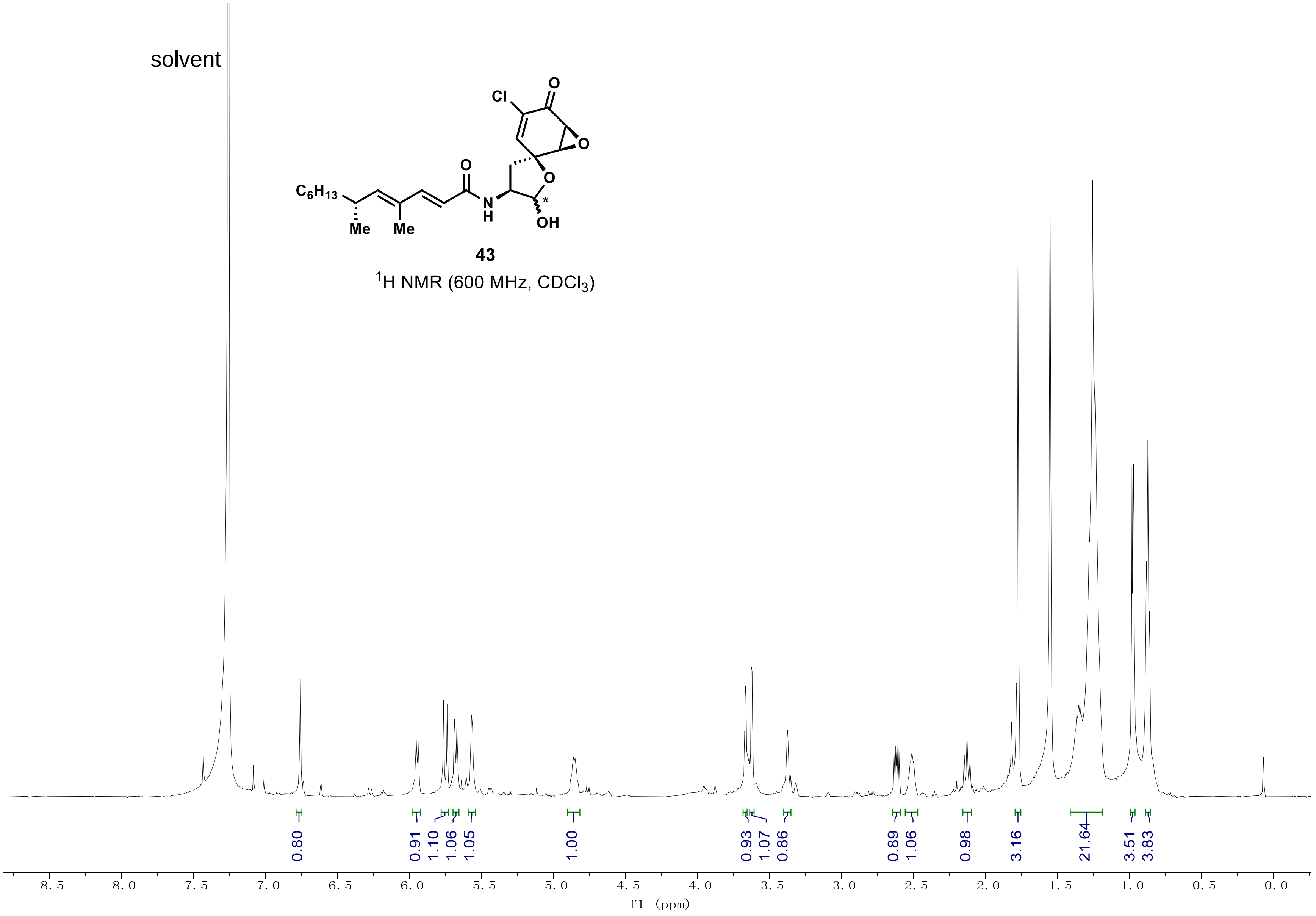

solvent
Cl
O
O
O
O
C6H13
N
H
Me
Me
OH
43
1H NMR (600 MHz, CDCl3)
0.80
0.91
1.10
1.06
1.05
1.00
0.93
1.07
0.86
0.89
1.06
0.98
3.16
21.64
3.51
3.83
8.5
8.0
7.5
7.0
6.5
6.0
5.5
5.0
4.5
4.0
3.5
3.0
2.5
2.0
1.5
1.0
0.5
0.0
f1 (ppm)

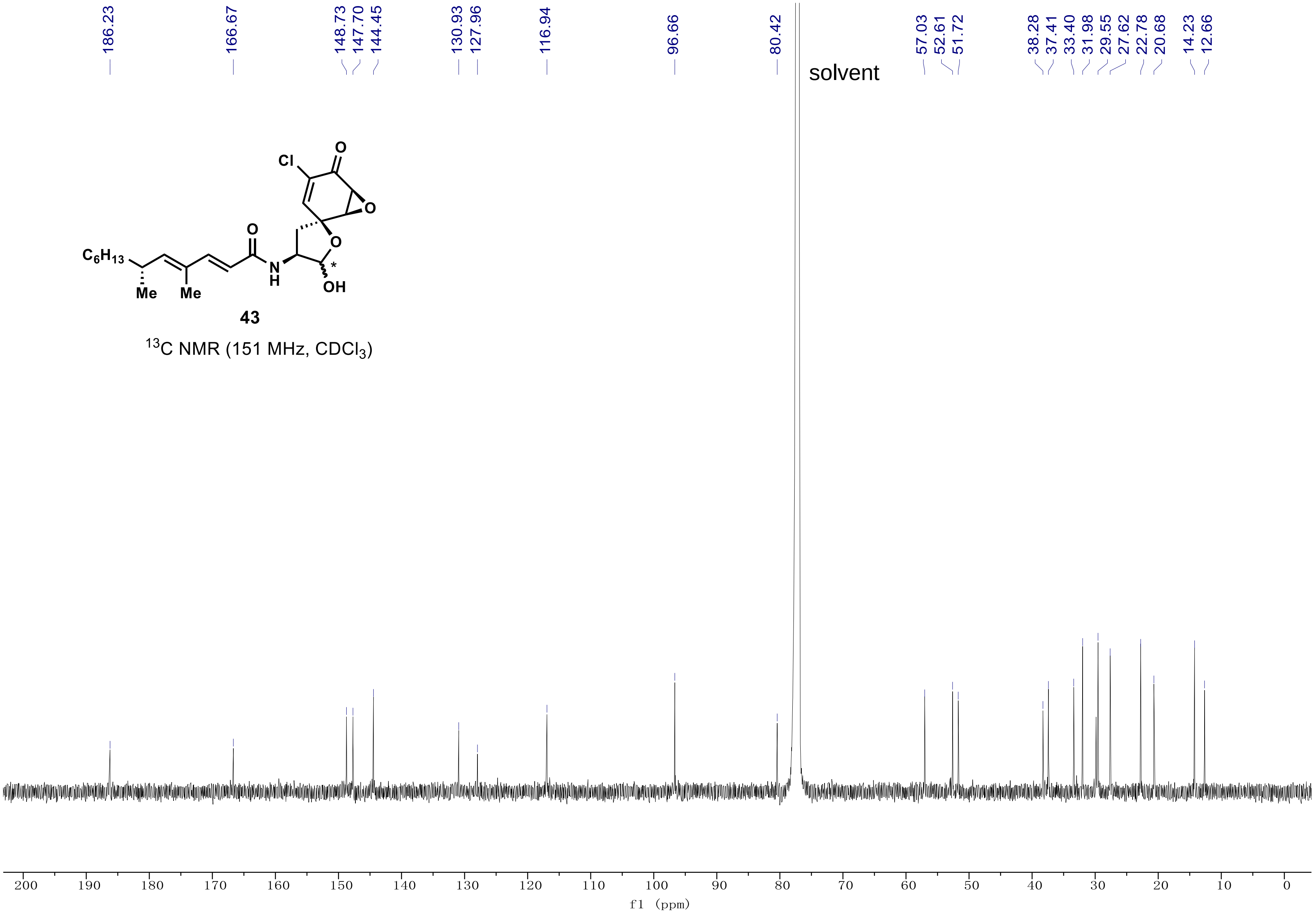

43

^{13}C NMR (151 MHz, $CDCl_3$)

2D-NOESY of selected compounds

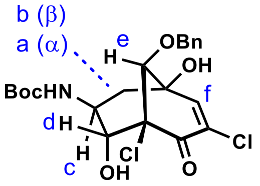

SI-1

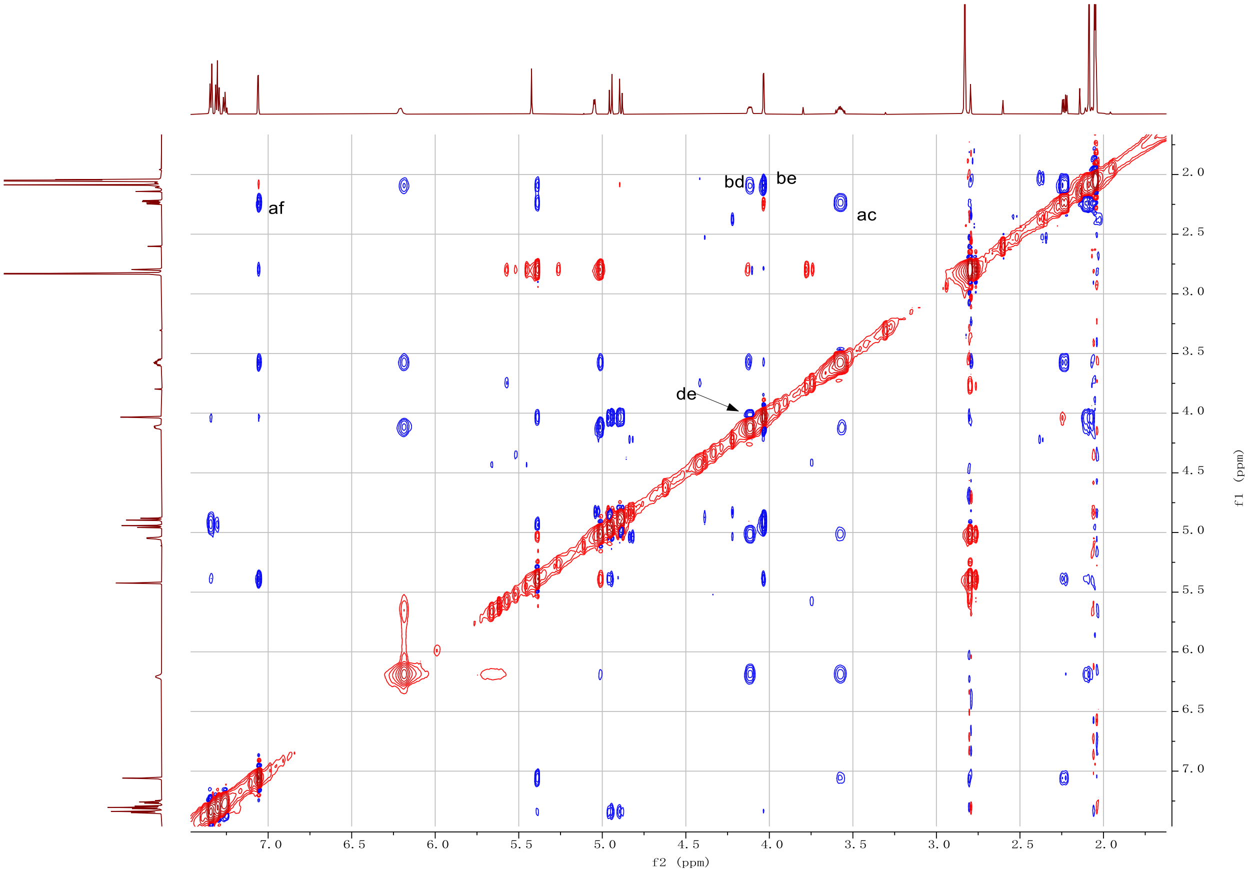

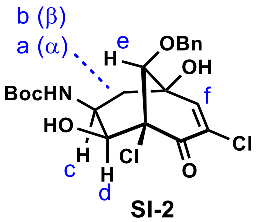
b (β)
a (α)
e
OBn
H
OH
f
BocHN
HO
Cl
Cl
H
H
O
c
d

SI-2

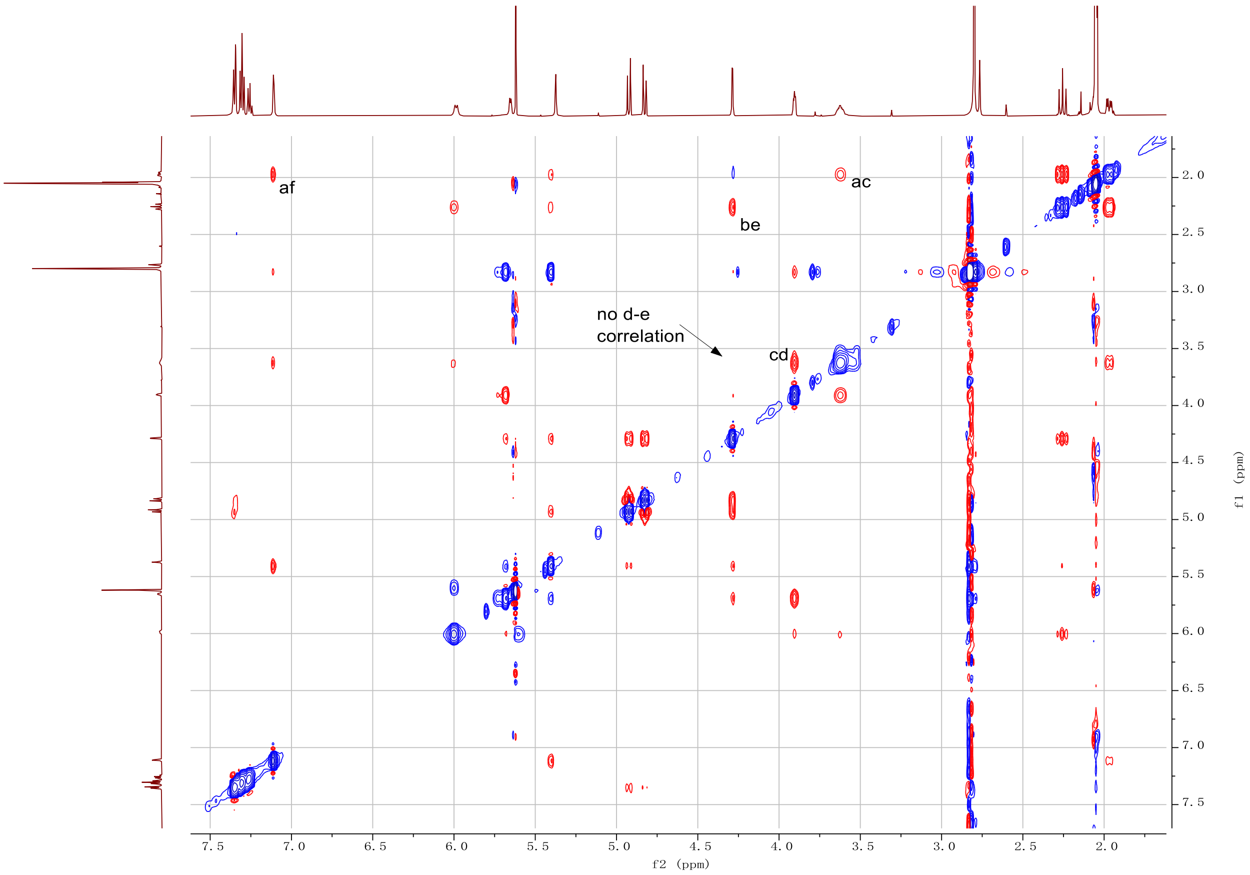
af
ac
be
no d-e correlation
cd
f2 (ppm)
f1 (ppm)

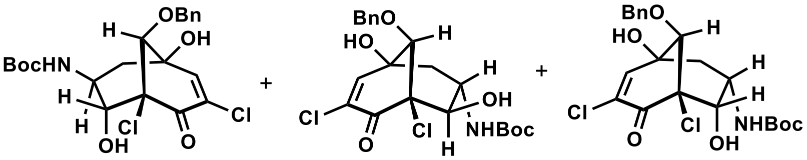

SI-1 SI-3

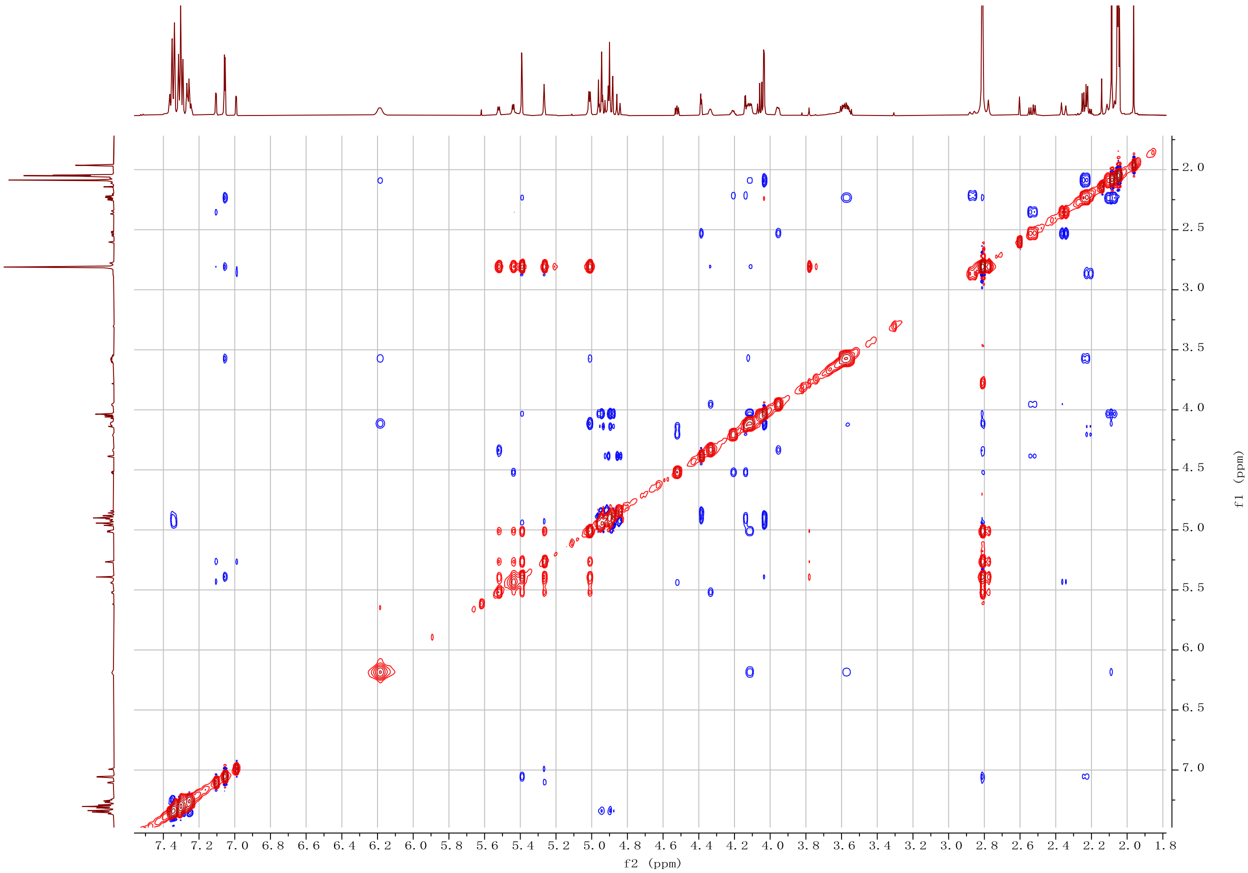

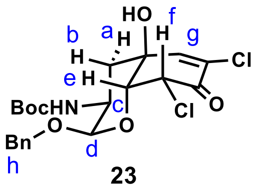

23

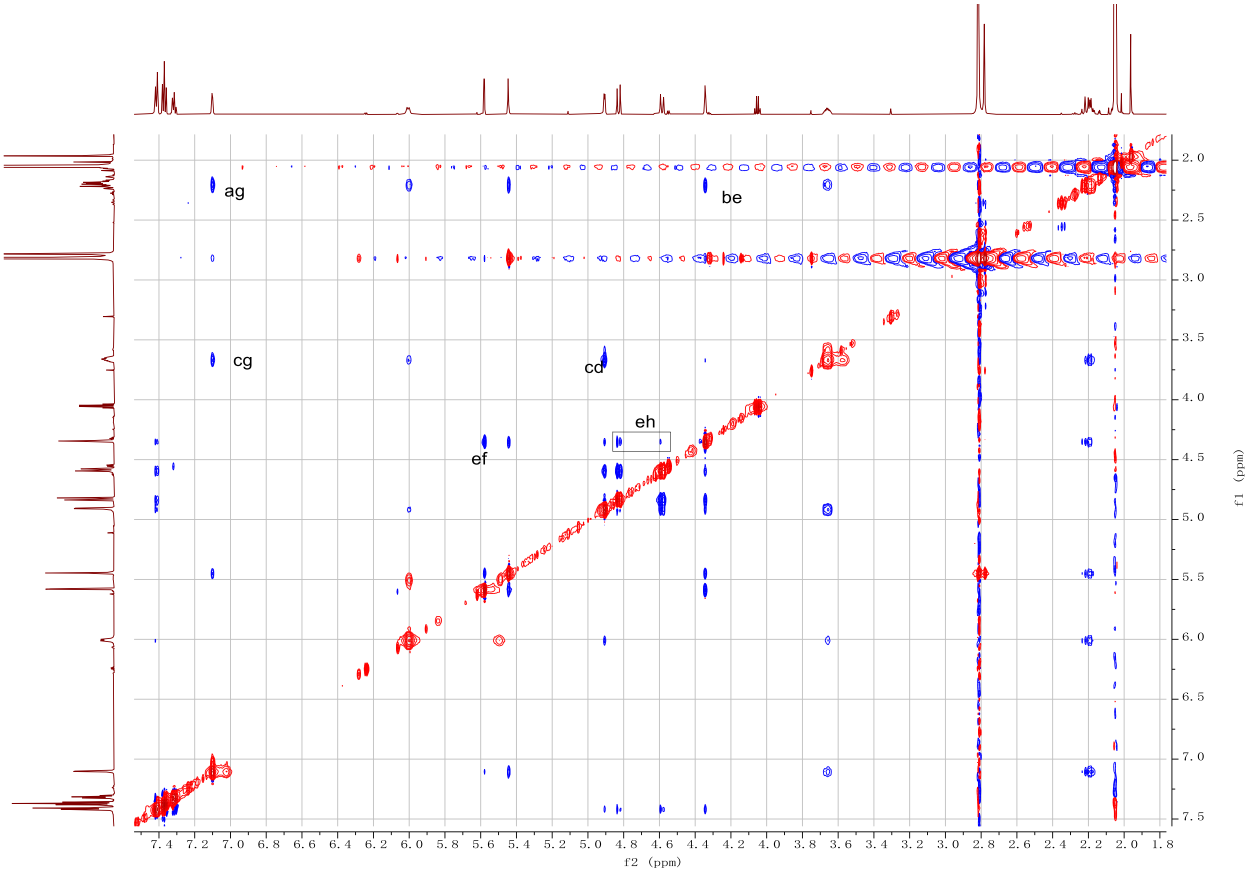

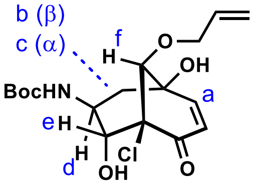

28a

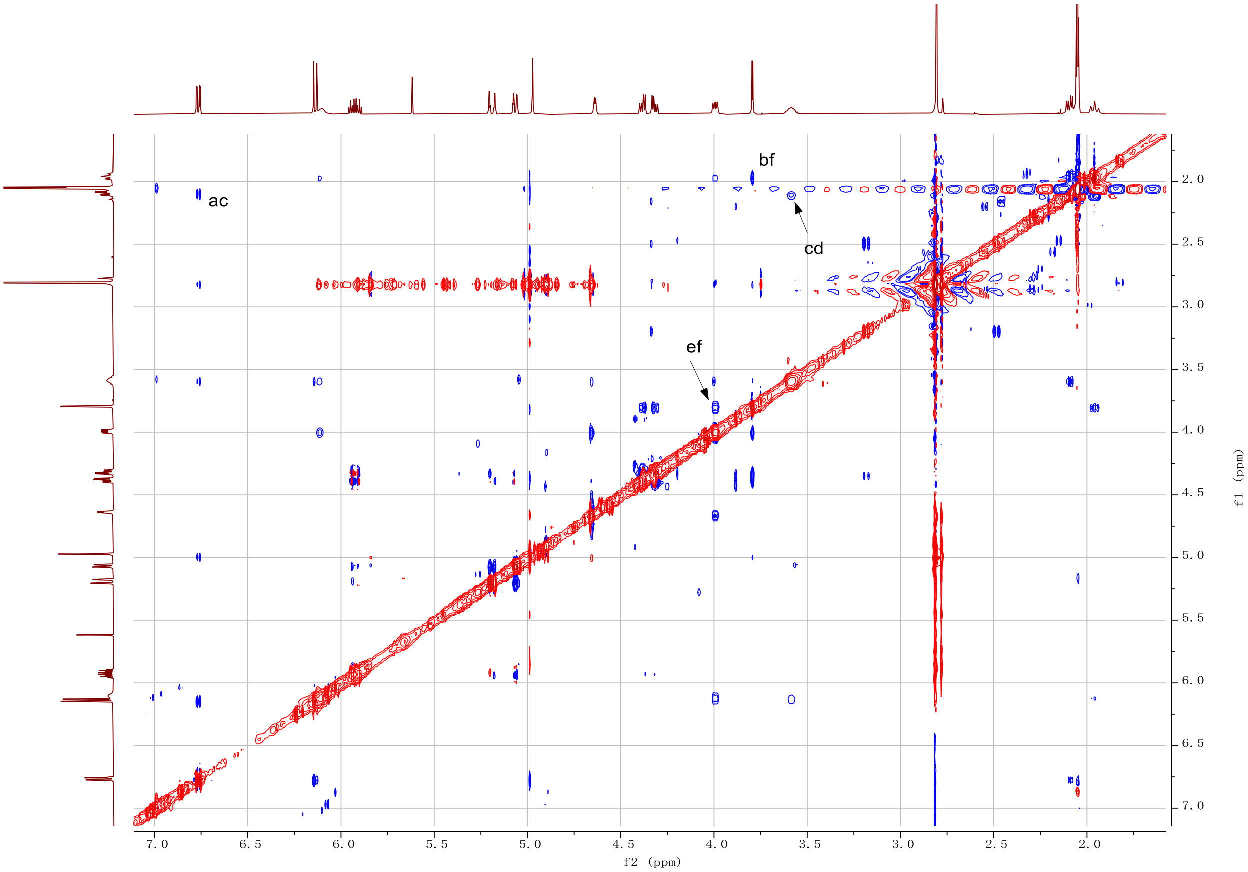

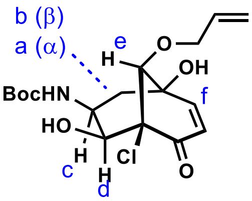

28b

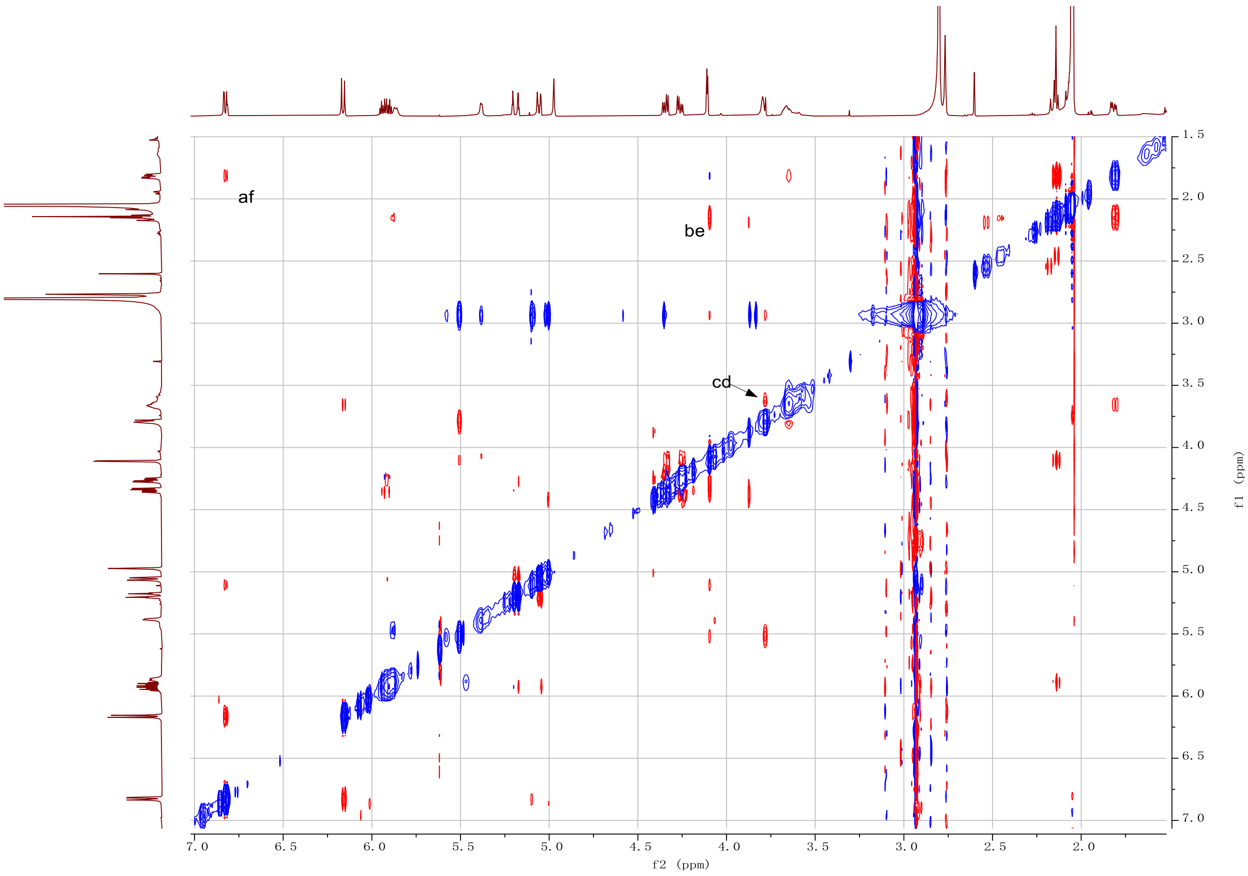

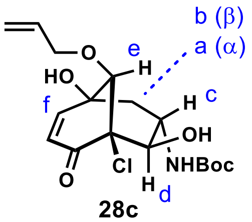

28c

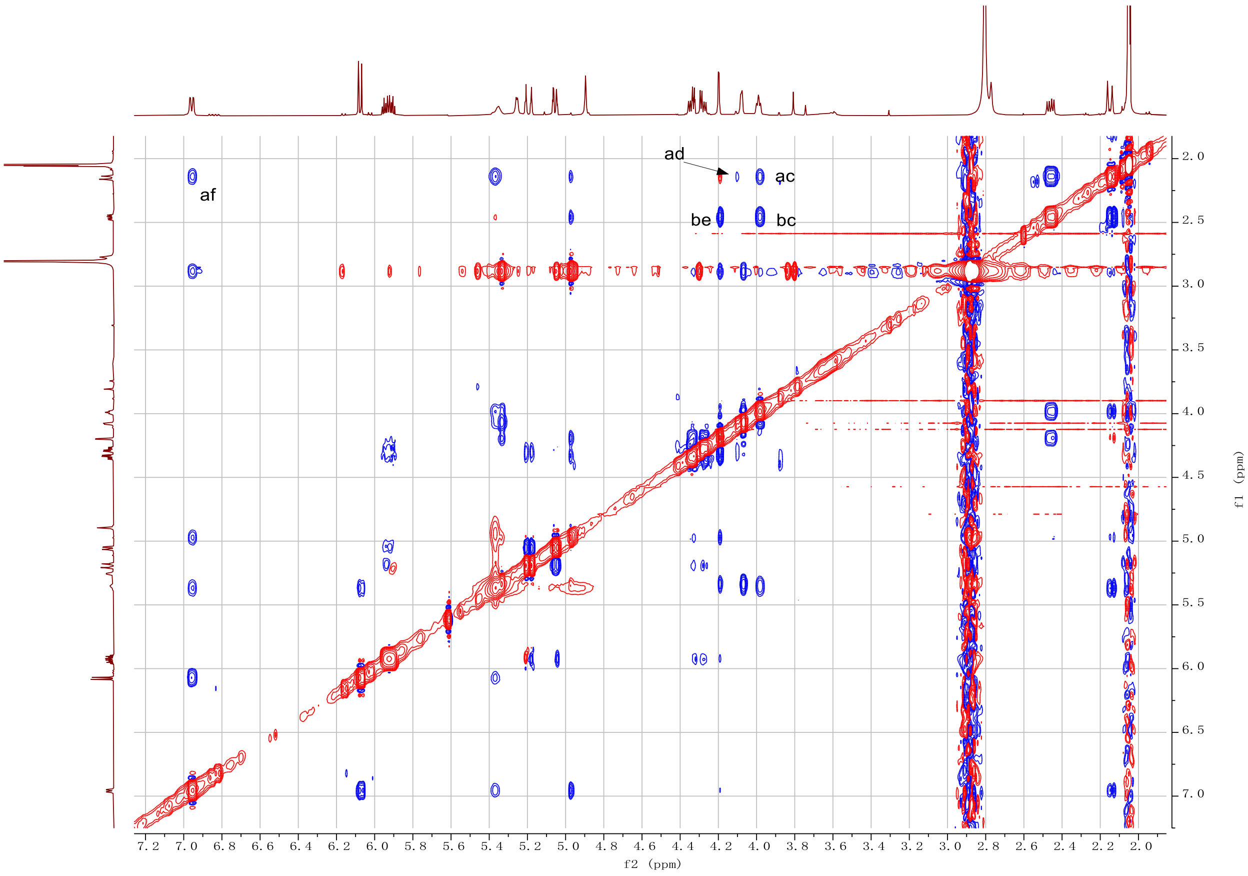

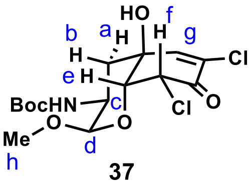
HO
f
H
g
a
H
b
H
e
H
Cl
BocHN
c
Cl
O
Me
O
O
d
h

37

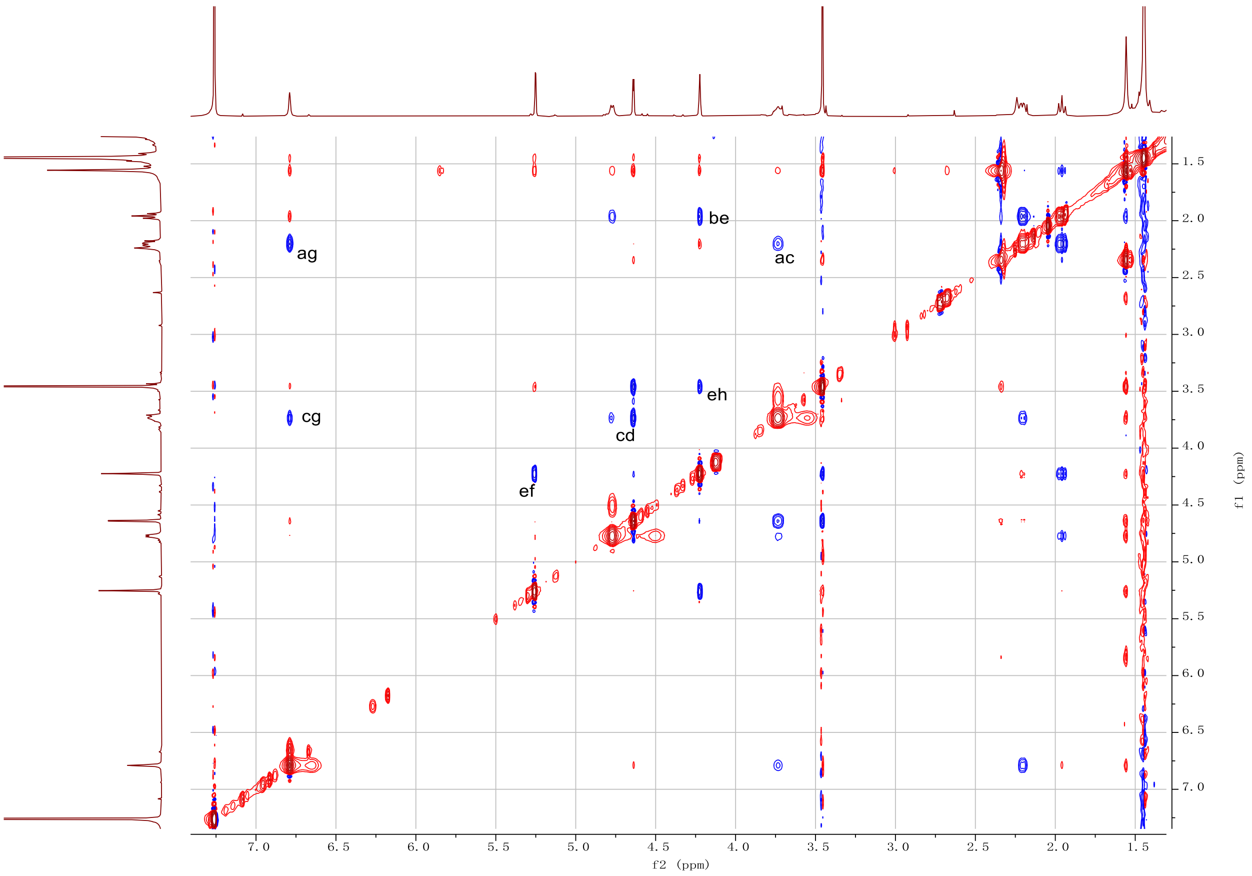
ag
be
ac
cg
eh
cd
ef
f2 (ppm)
f1 (ppm)

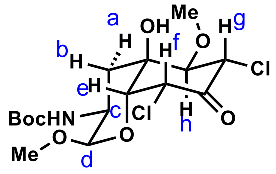

38

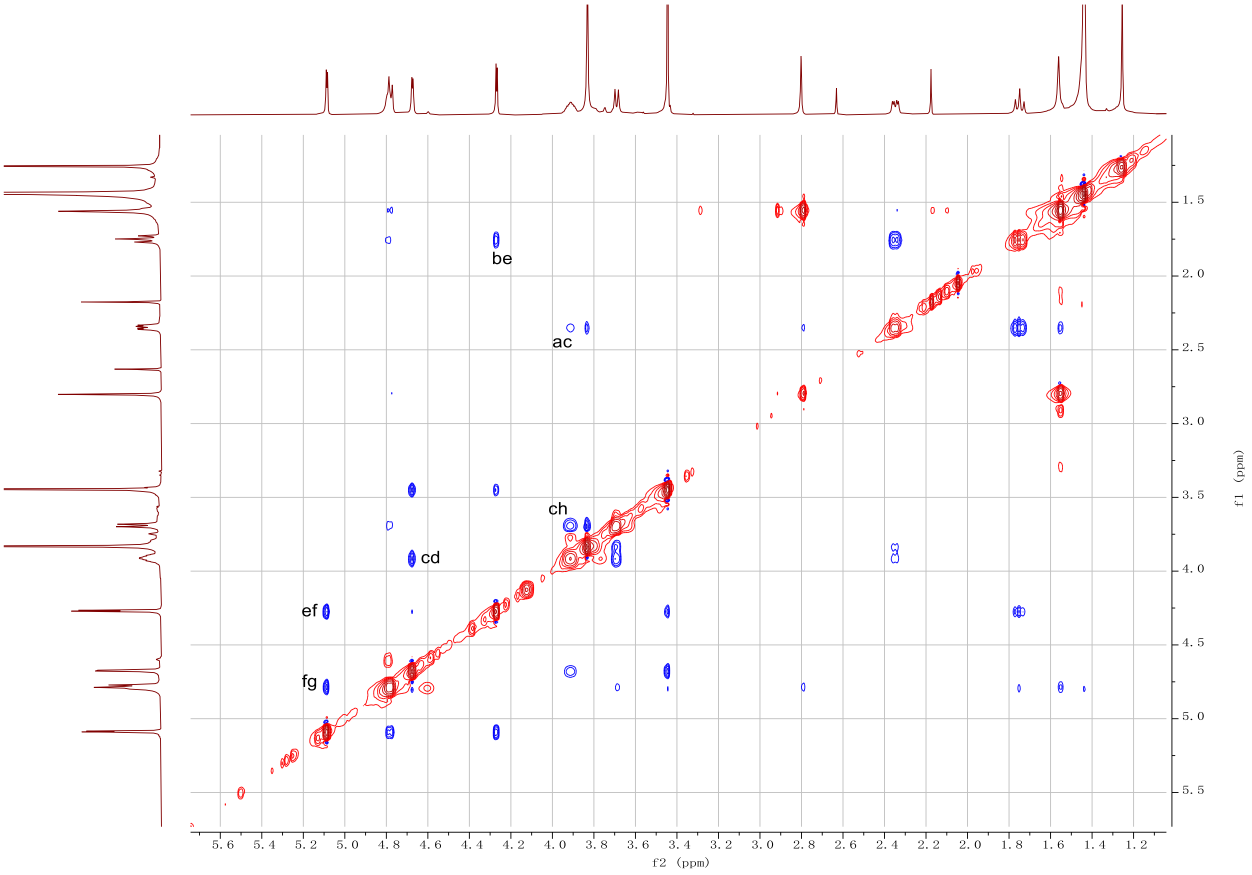

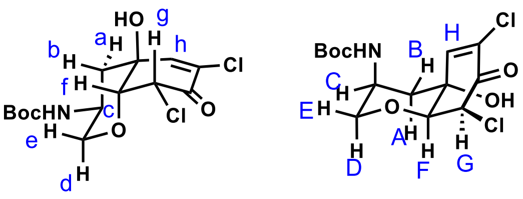

42
iso-42

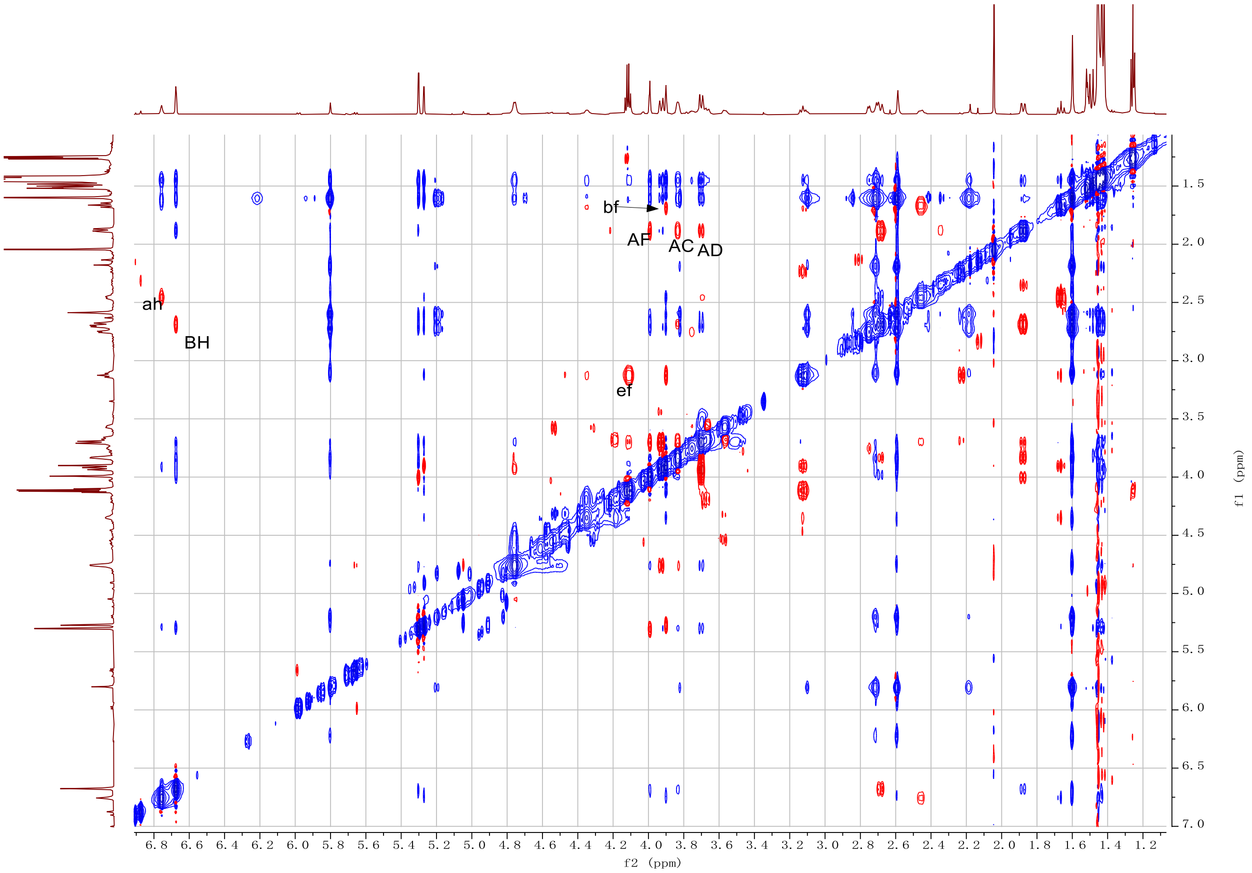

bf
AF
AC
AD
ah
BH
ef
f2 (ppm)
f1 (ppm)
